# Supplementary figures and images for: Identification of Individuals of Two Takin Subspecies Using Biological and Ecological Criteria in Eastern Himalayas of China
Source: Animals (Basel). 2024 Aug 21;14(16):2426. doi: 10.3390/ani14162426 (PMC11350688; doi:10.3390/ani14162426)

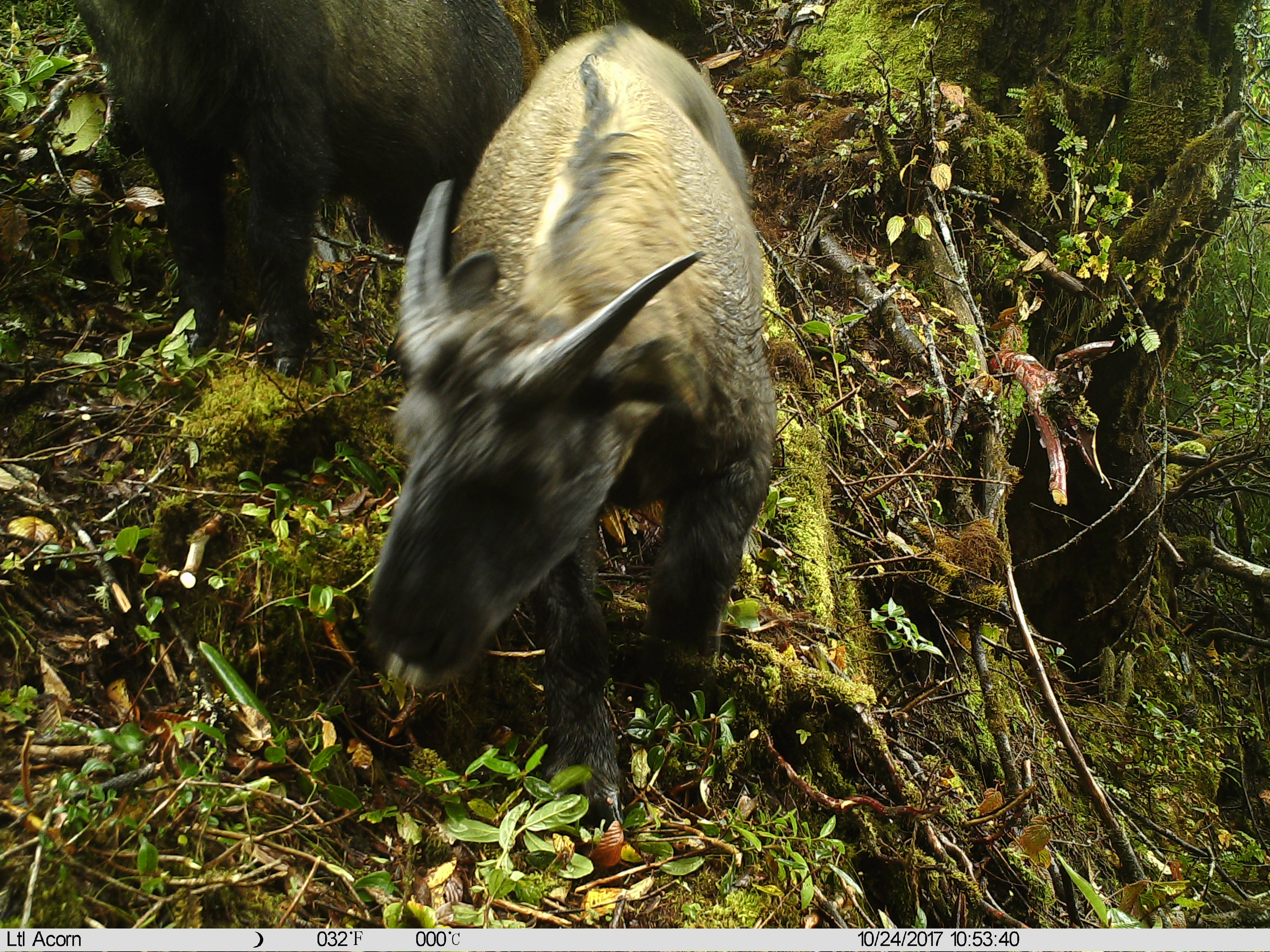

Supplement: Supplementary file 1 [file animals-14-02426-s001.zip › Budorcas taxicolor taxicolor-Part of the photos/IMAG0009.JPG]

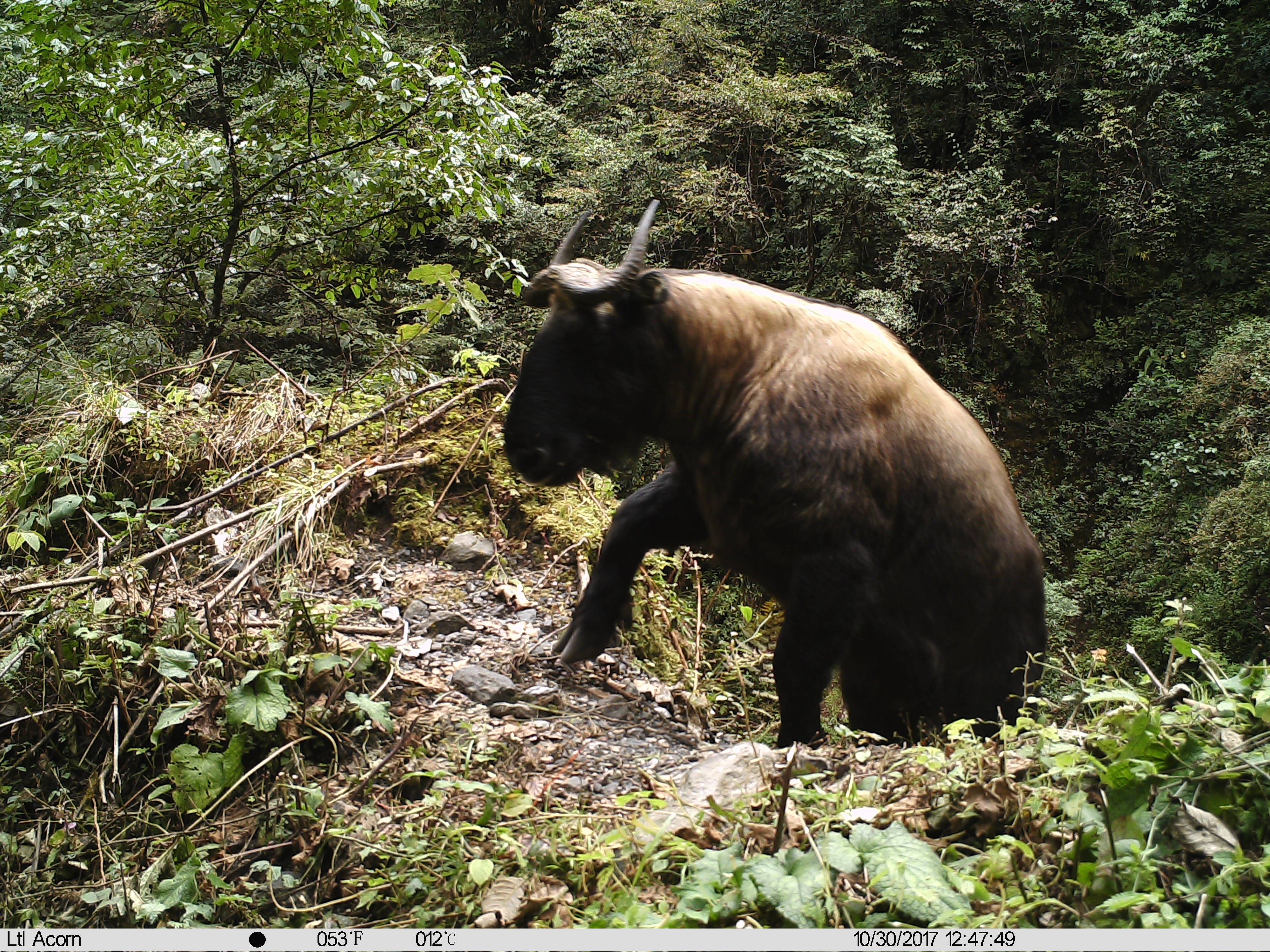

Supplement: Supplementary file 1 [file animals-14-02426-s001.zip › Budorcas taxicolor taxicolor-Part of the photos/IMAG0014 (2).JPG]

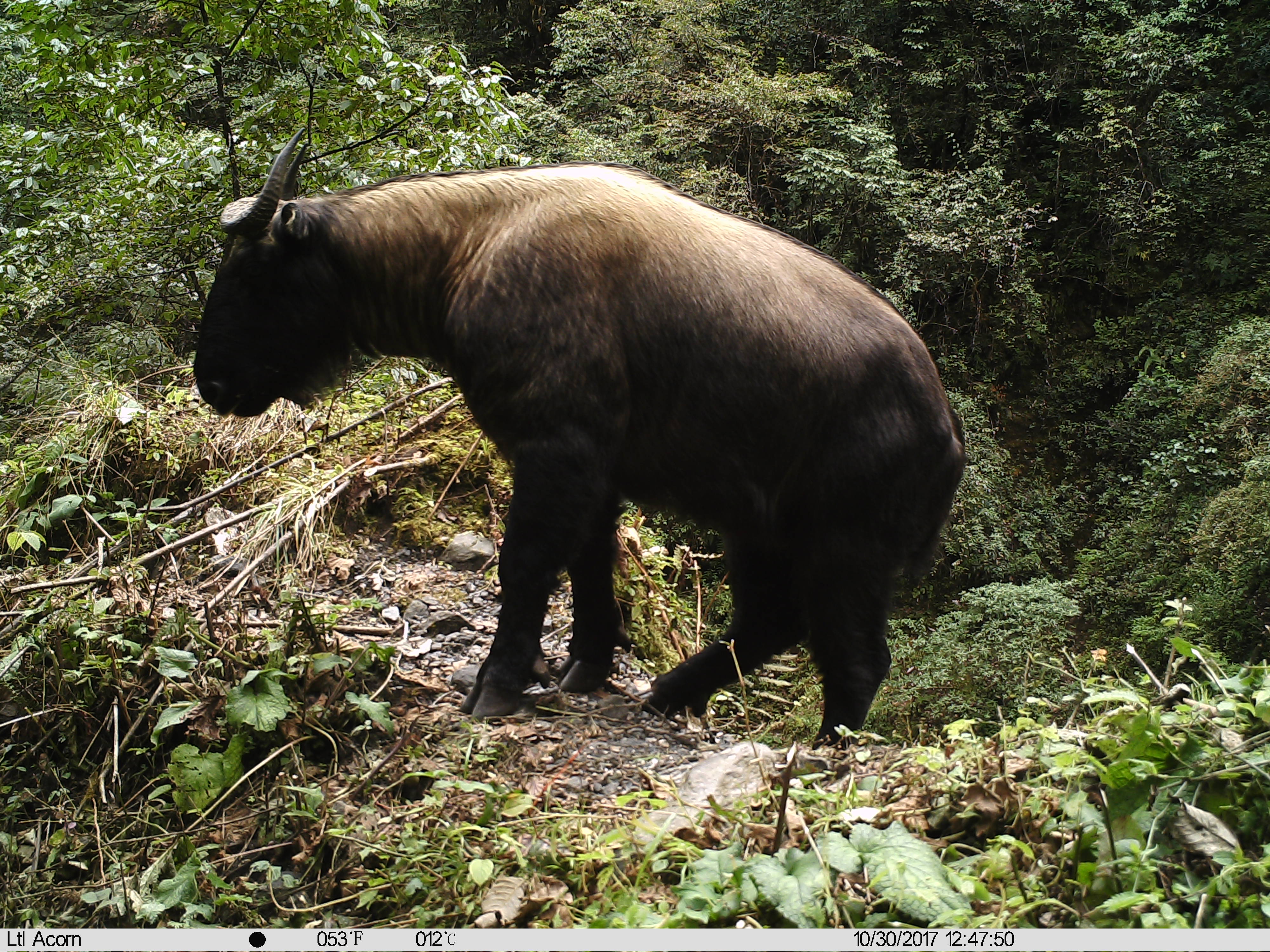

Supplement: Supplementary file 1 [file animals-14-02426-s001.zip › Budorcas taxicolor taxicolor-Part of the photos/IMAG0015 (2).JPG]

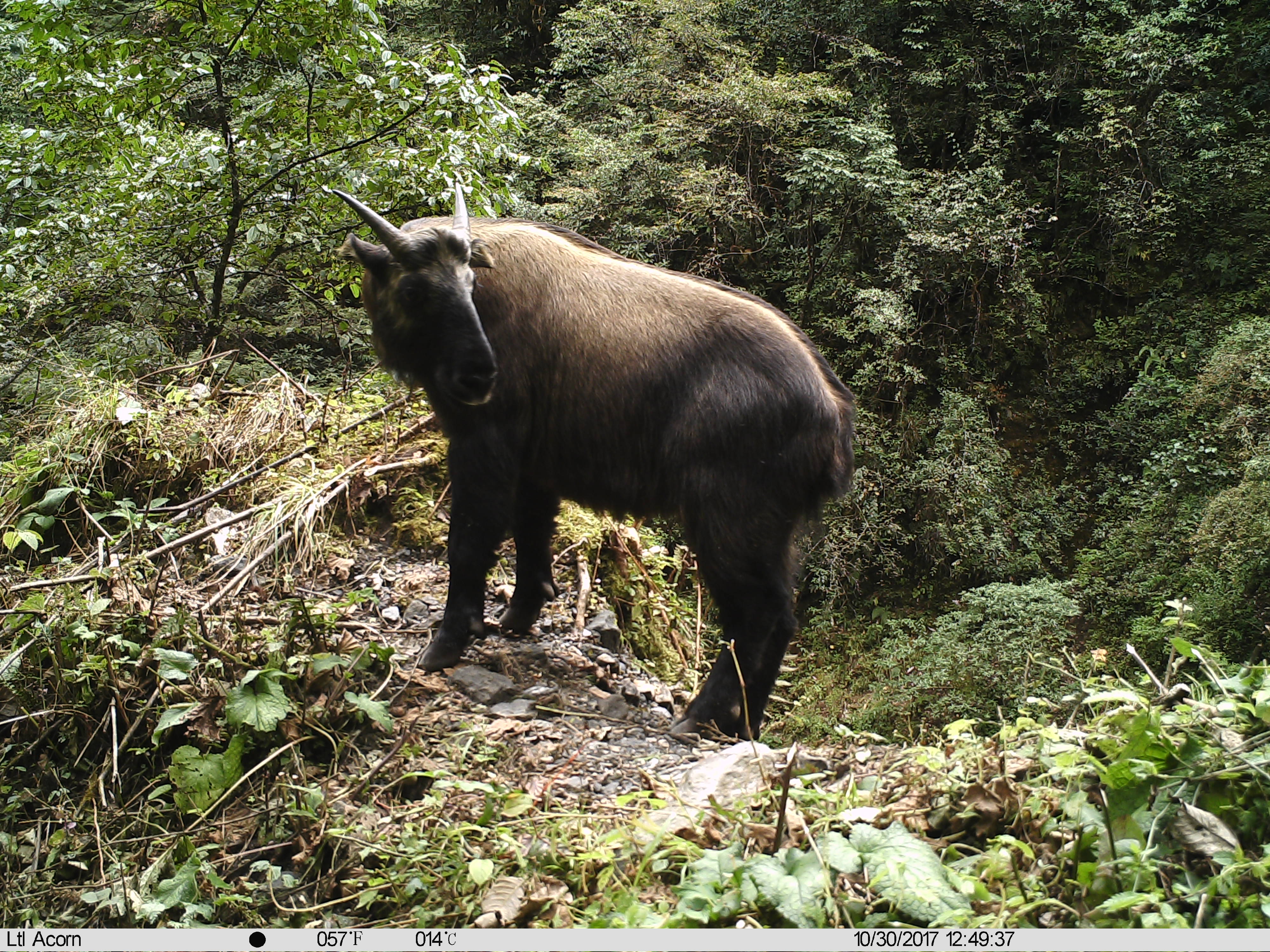

Supplement: Supplementary file 1 [file animals-14-02426-s001.zip › Budorcas taxicolor taxicolor-Part of the photos/IMAG0022.JPG]

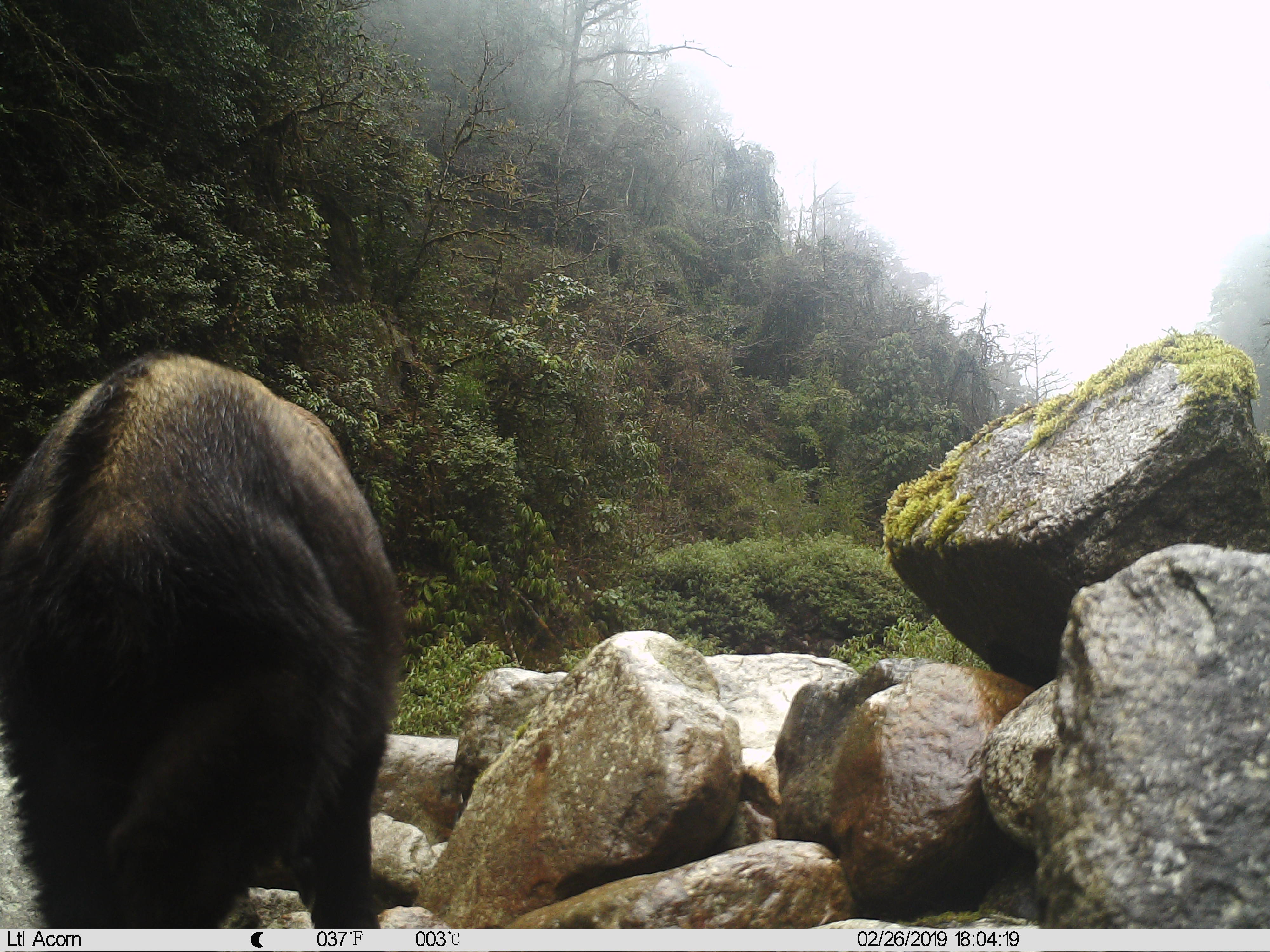

Supplement: Supplementary file 1 [file animals-14-02426-s001.zip › Budorcas taxicolor taxicolor-Part of the photos/IMAG0087 (2).JPG]

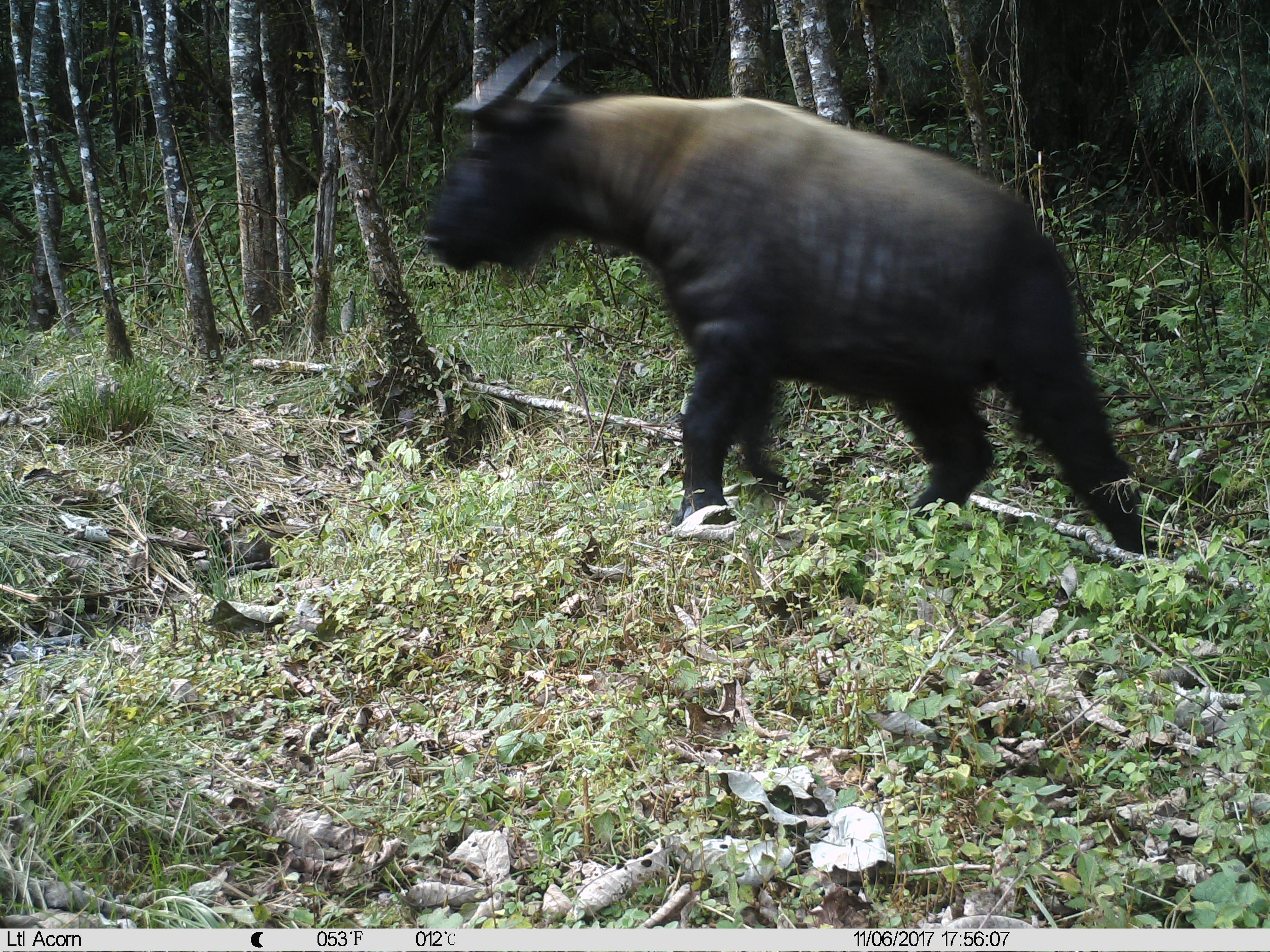

Supplement: Supplementary file 1 [file animals-14-02426-s001.zip › Budorcas taxicolor taxicolor-Part of the photos/IMAG0118 (4).JPG]

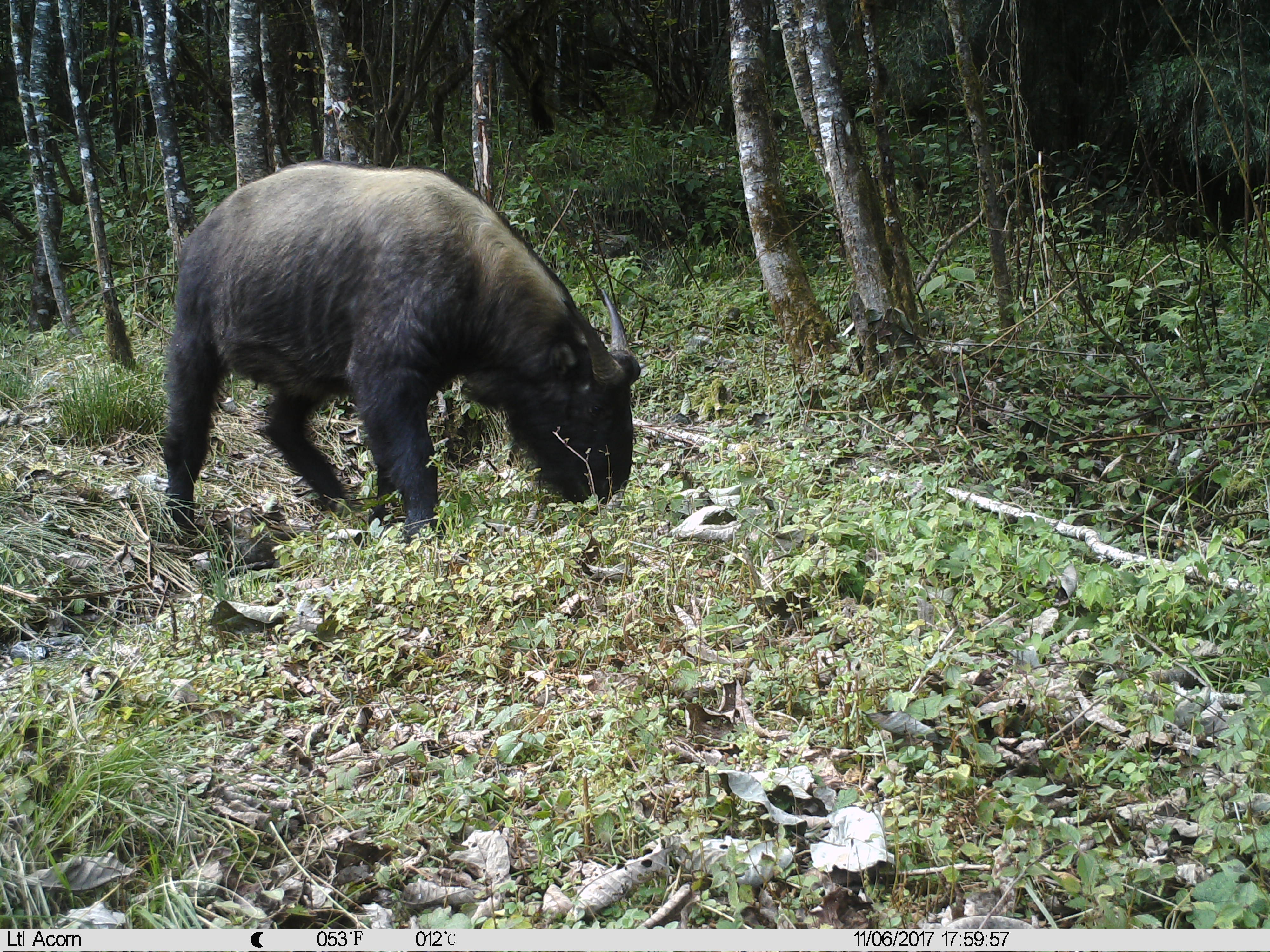

Supplement: Supplementary file 1 [file animals-14-02426-s001.zip › Budorcas taxicolor taxicolor-Part of the photos/IMAG0121 (3).JPG]

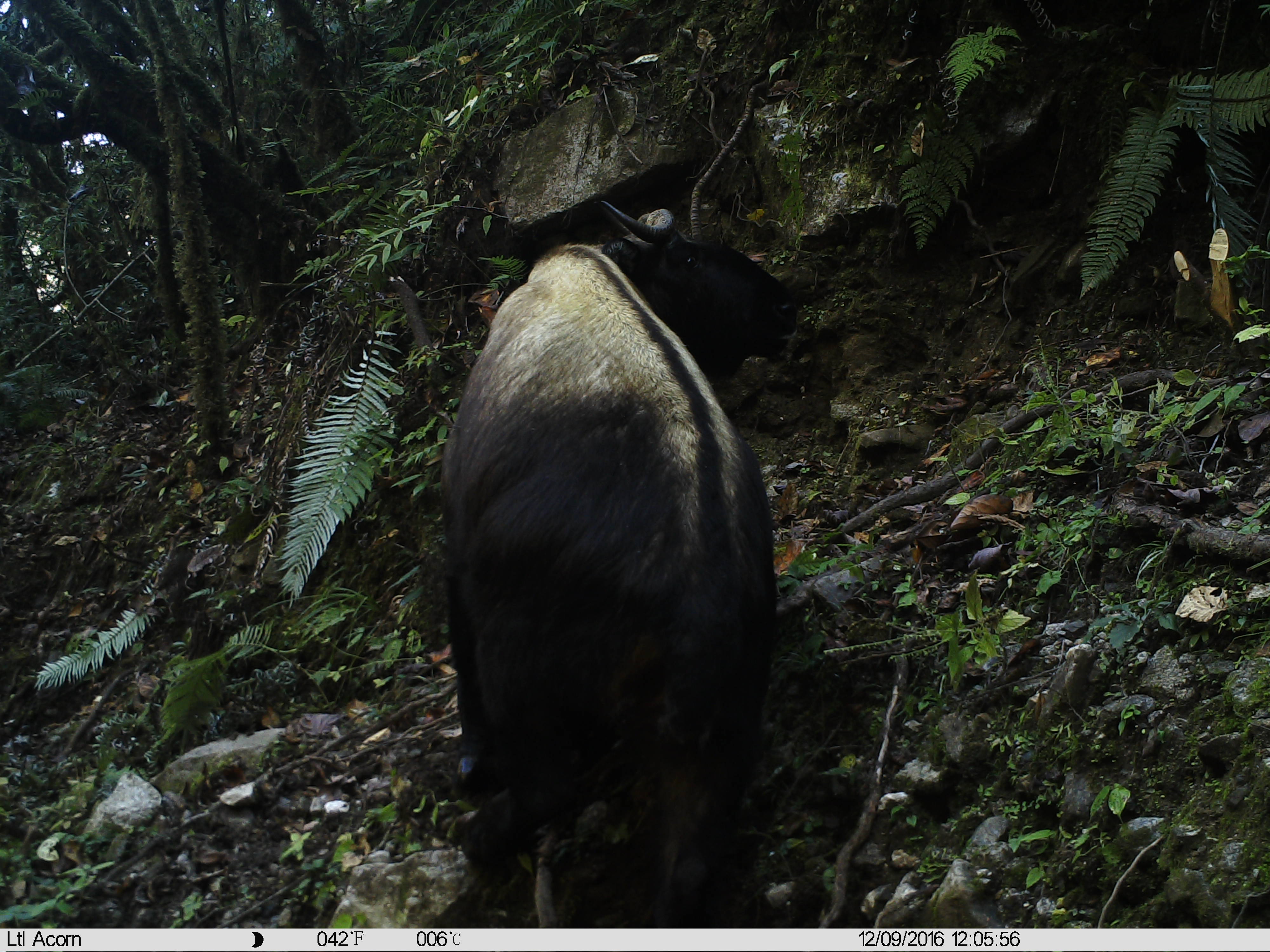

Supplement: Supplementary file 1 [file animals-14-02426-s001.zip › Budorcas taxicolor taxicolor-Part of the photos/IMAG0143.JPG]

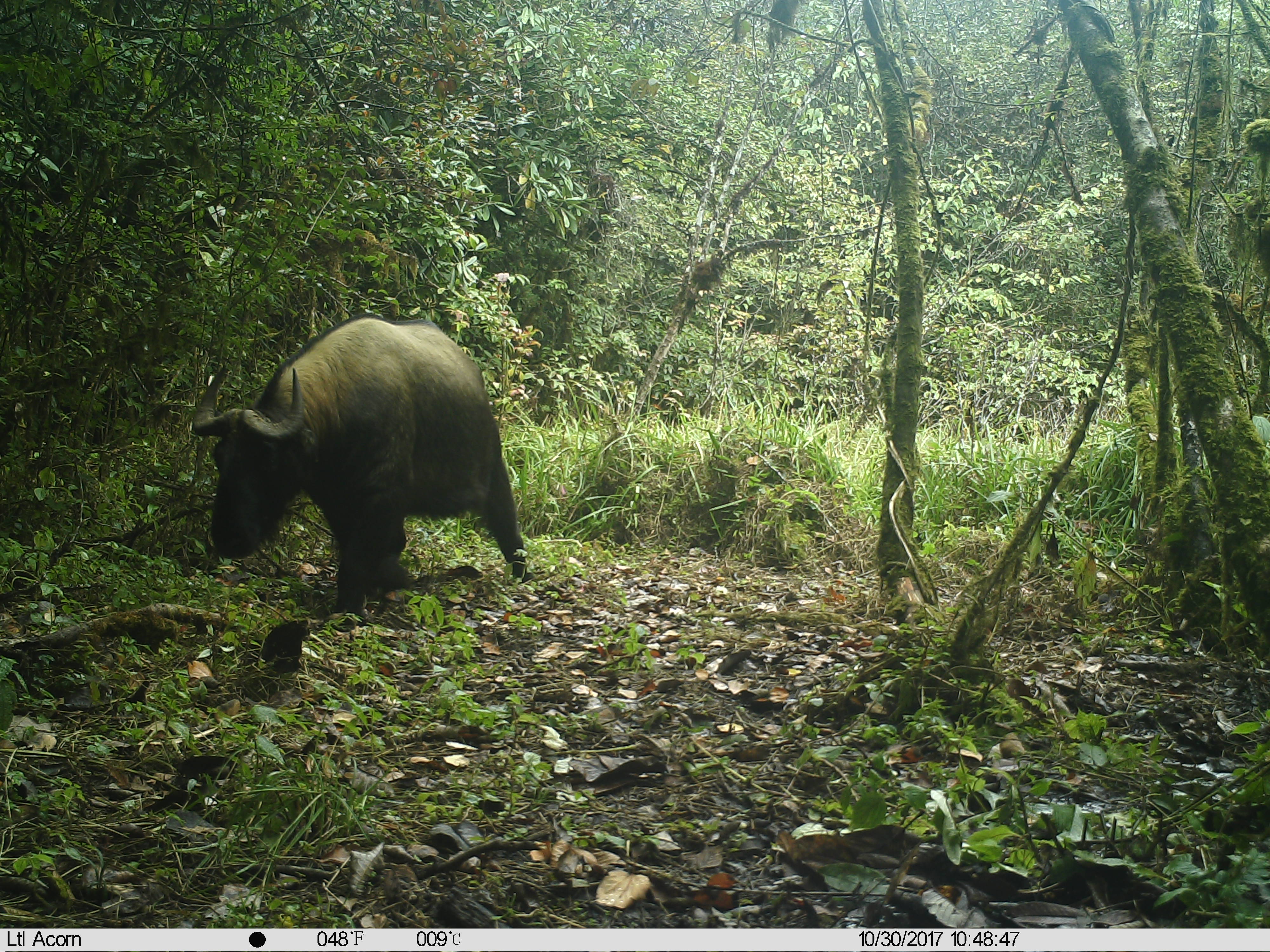

Supplement: Supplementary file 1 [file animals-14-02426-s001.zip › Budorcas taxicolor taxicolor-Part of the photos/IMAG0162 (2).JPG]

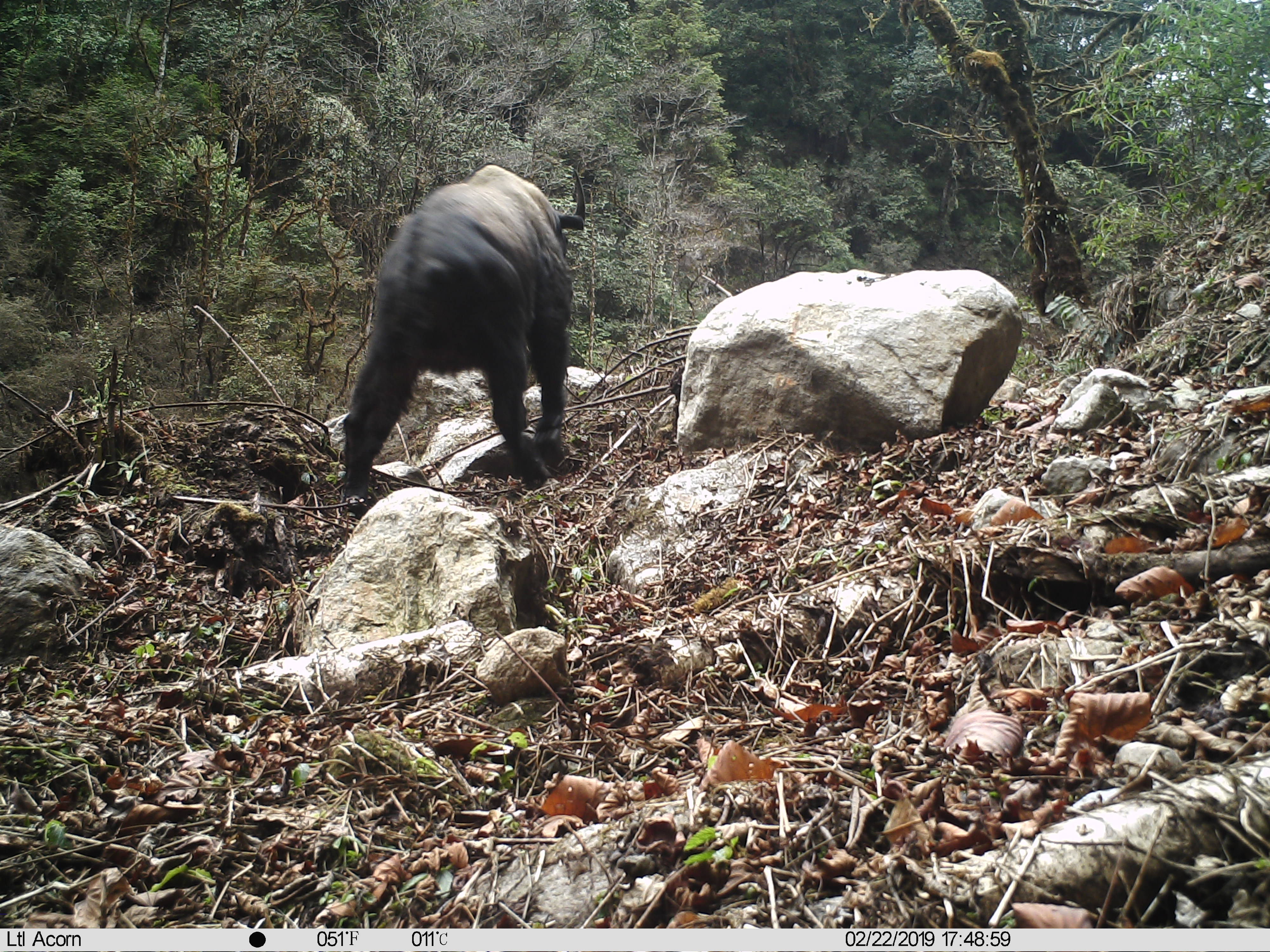

Supplement: Supplementary file 1 [file animals-14-02426-s001.zip › Budorcas taxicolor taxicolor-Part of the photos/IMAG0214 (2).JPG]

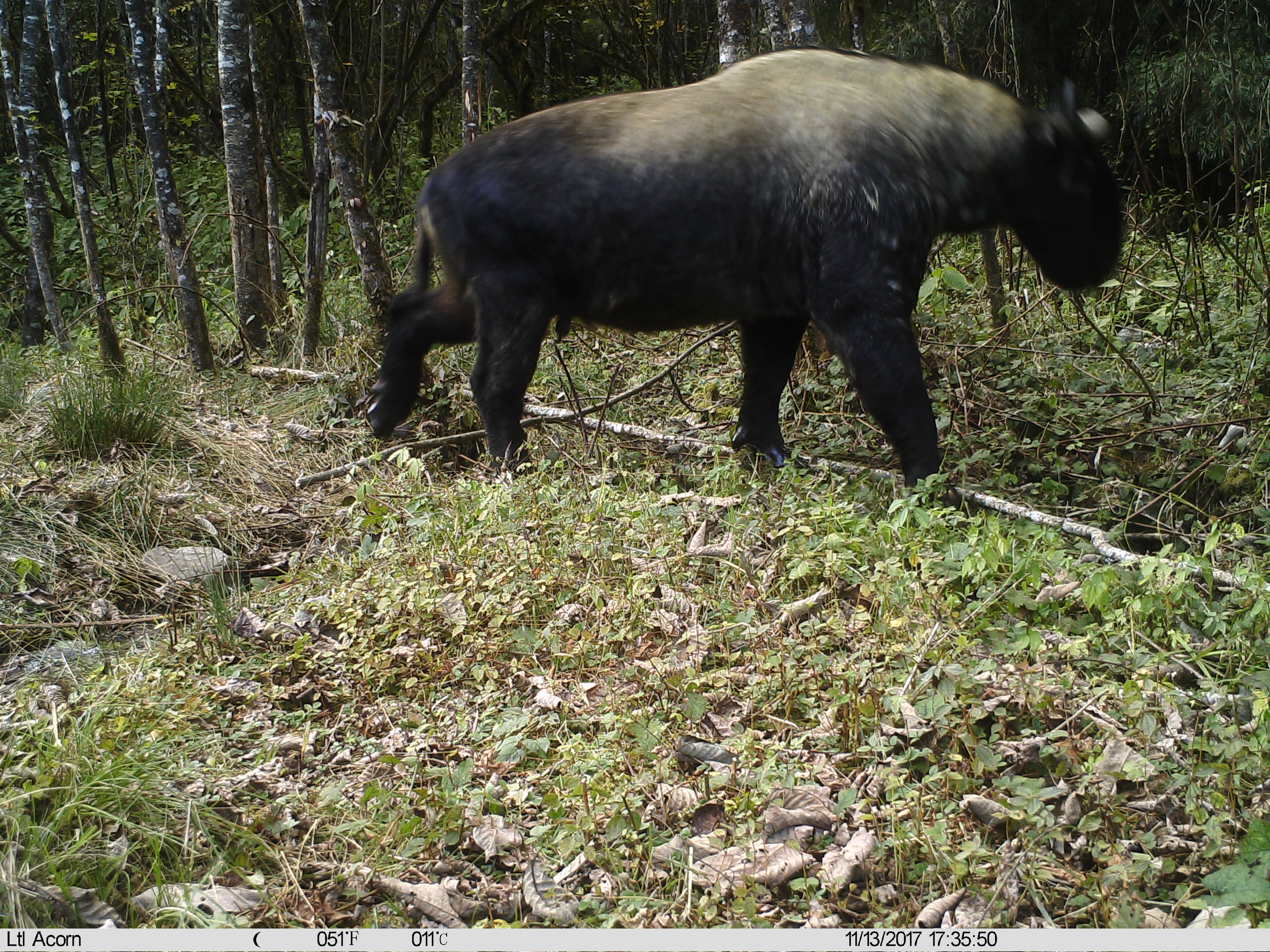

Supplement: Supplementary file 1 [file animals-14-02426-s001.zip › Budorcas taxicolor taxicolor-Part of the photos/IMAG0246 (3).JPG]

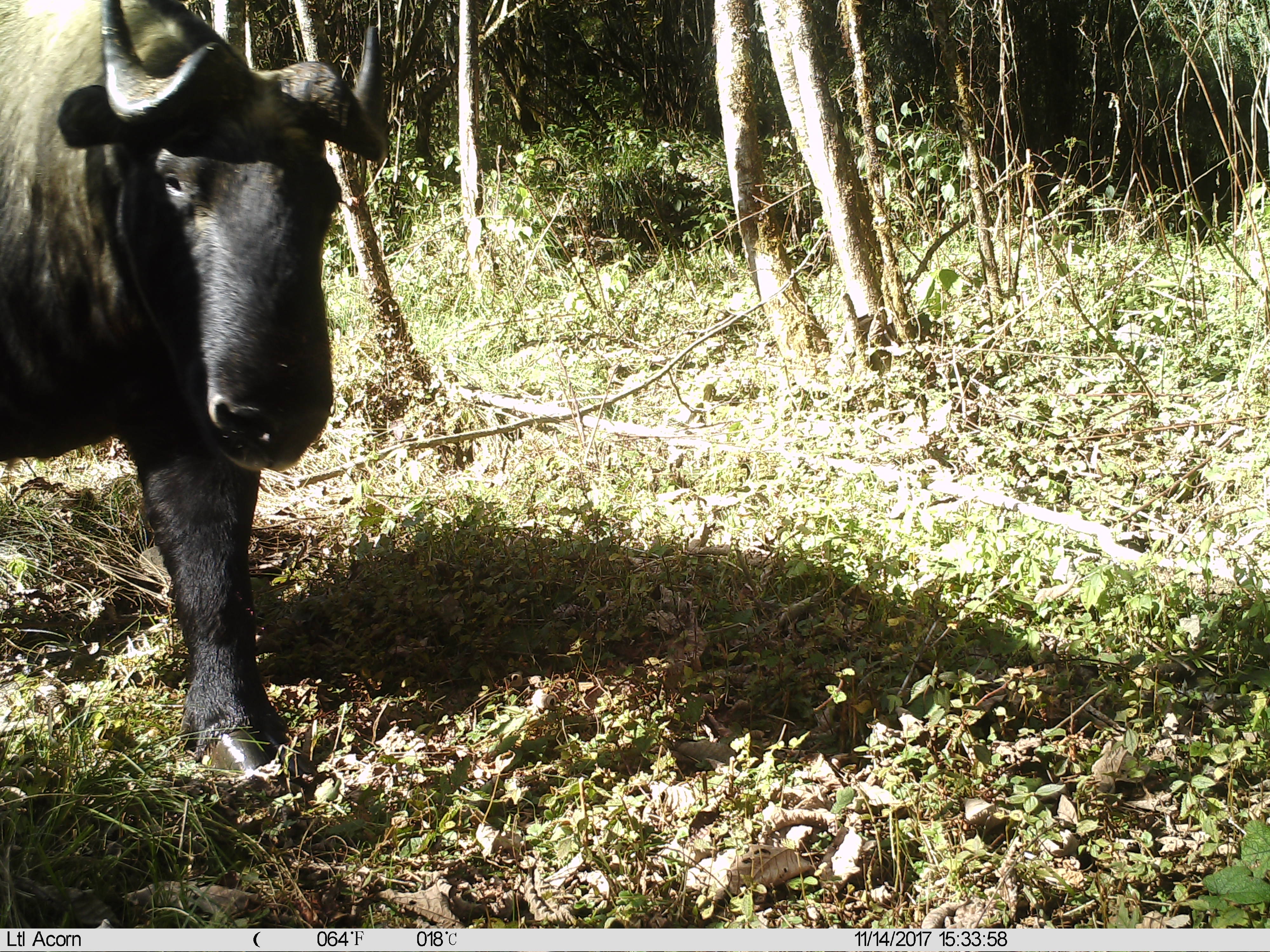

Supplement: Supplementary file 1 [file animals-14-02426-s001.zip › Budorcas taxicolor taxicolor-Part of the photos/IMAG0249 (2).JPG]

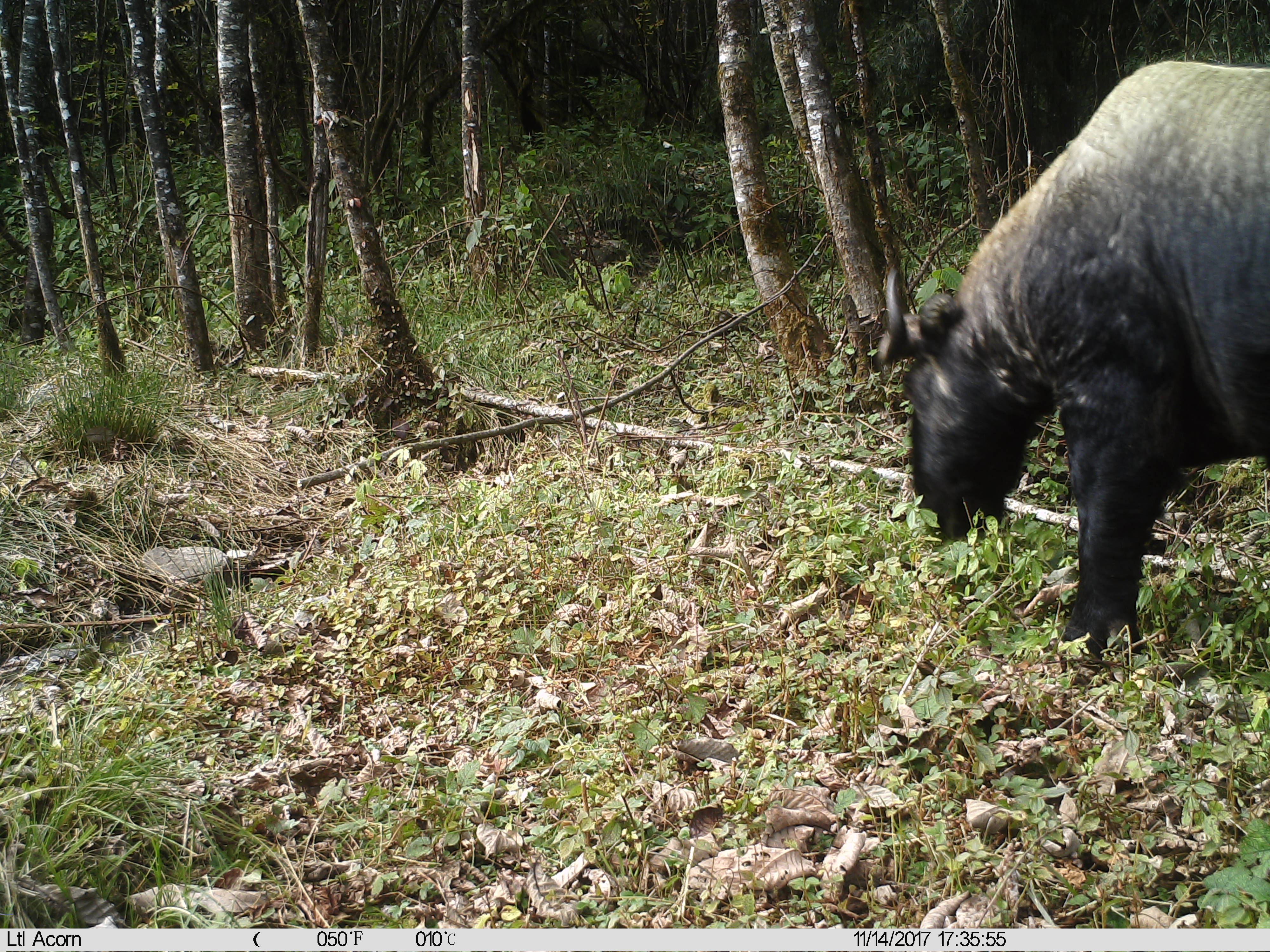

Supplement: Supplementary file 1 [file animals-14-02426-s001.zip › Budorcas taxicolor taxicolor-Part of the photos/IMAG0255 (2).JPG]

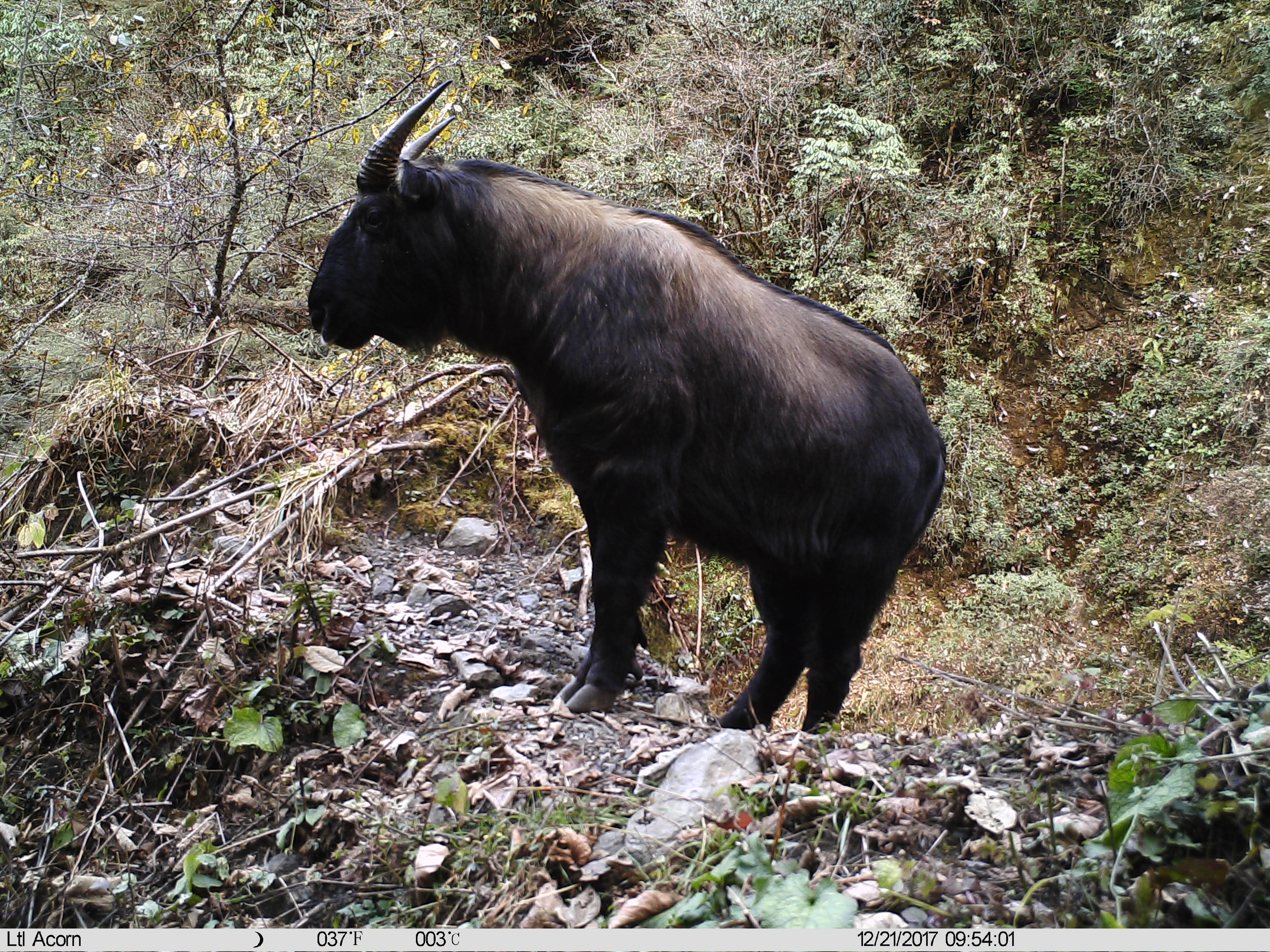

Supplement: Supplementary file 1 [file animals-14-02426-s001.zip › Budorcas taxicolor taxicolor-Part of the photos/IMAG0266 (2).JPG]

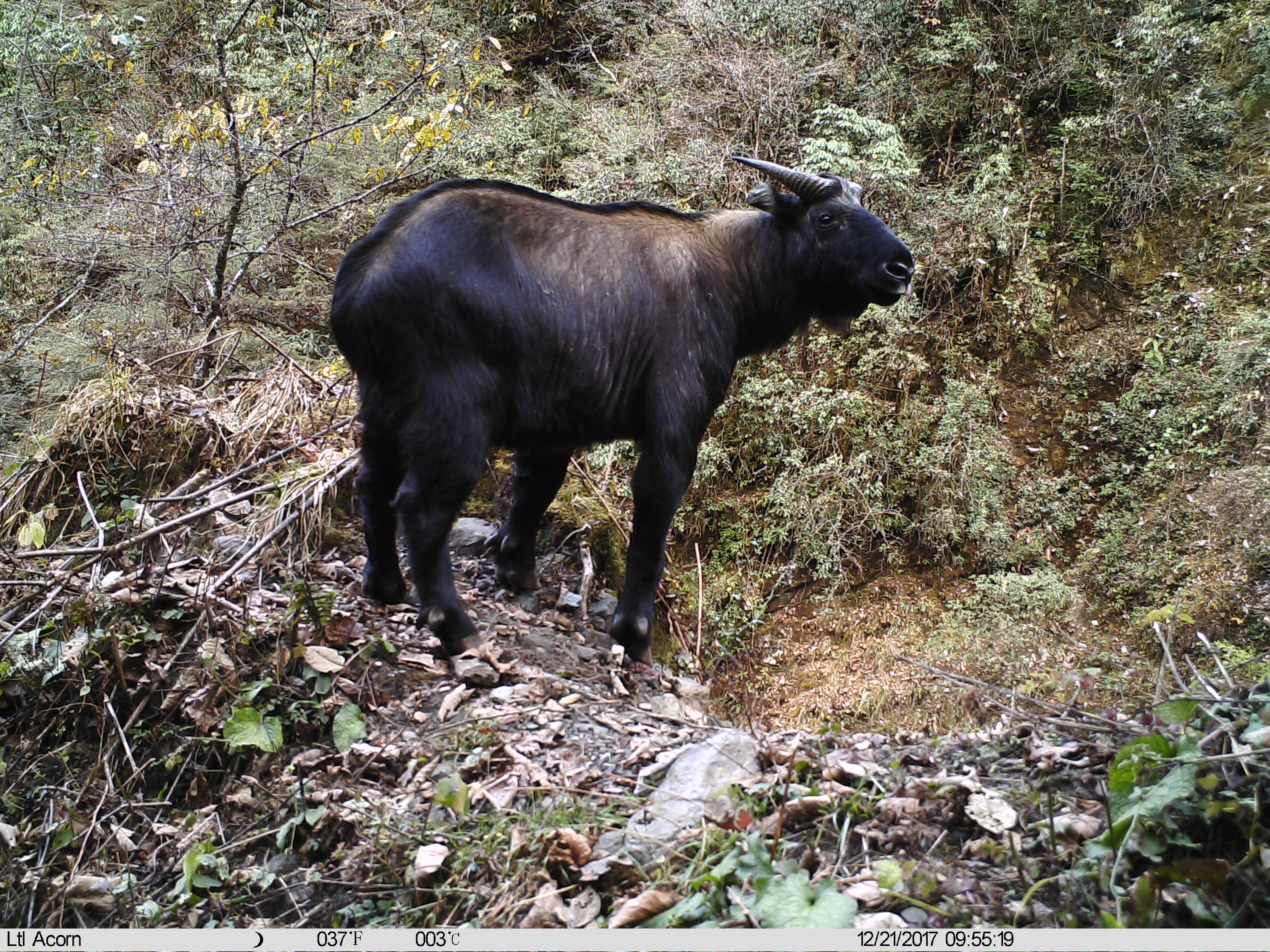

Supplement: Supplementary file 1 [file animals-14-02426-s001.zip › Budorcas taxicolor taxicolor-Part of the photos/IMAG0270 (2).JPG]

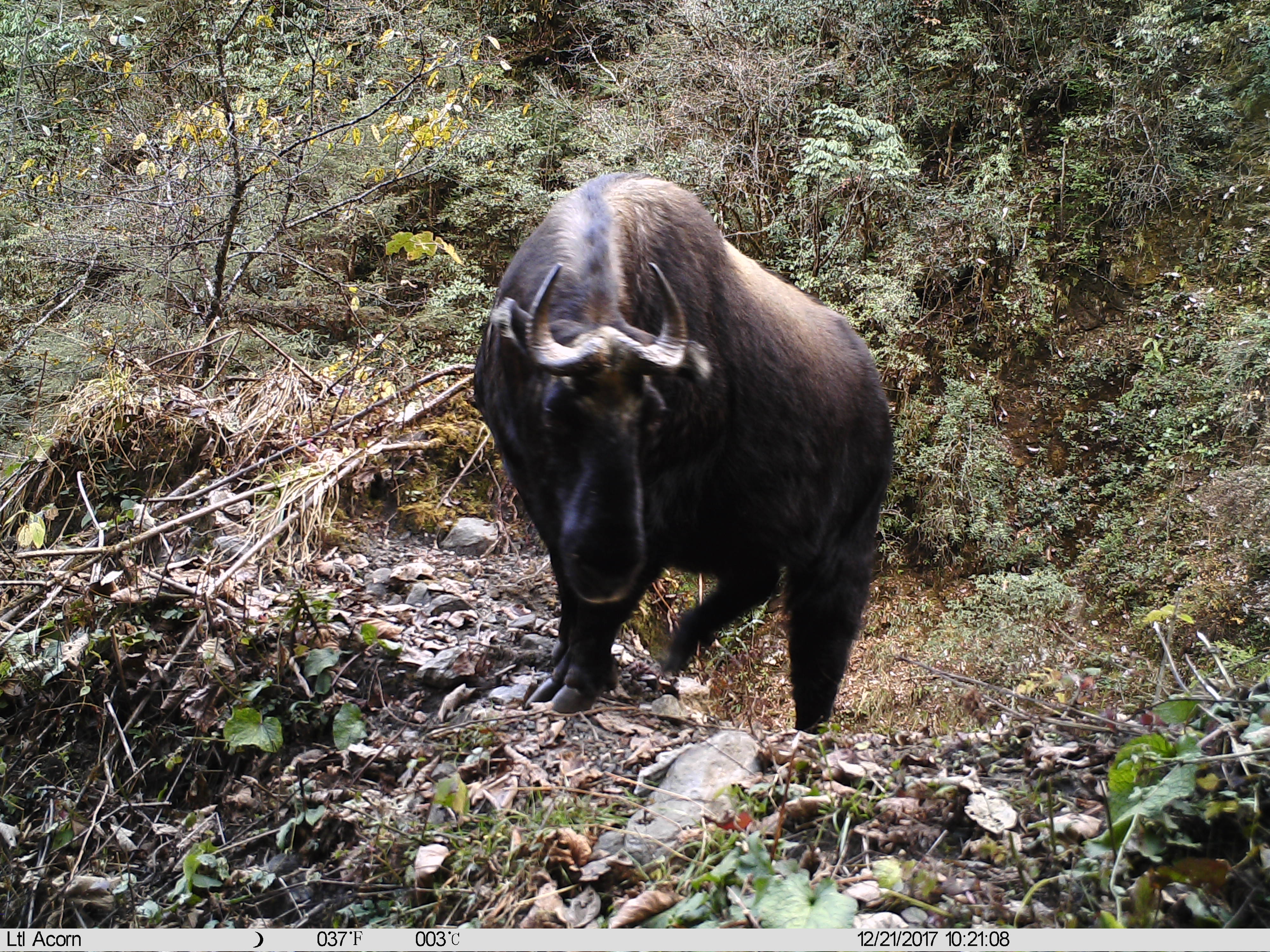

Supplement: Supplementary file 1 [file animals-14-02426-s001.zip › Budorcas taxicolor taxicolor-Part of the photos/IMAG0285 (2).JPG]

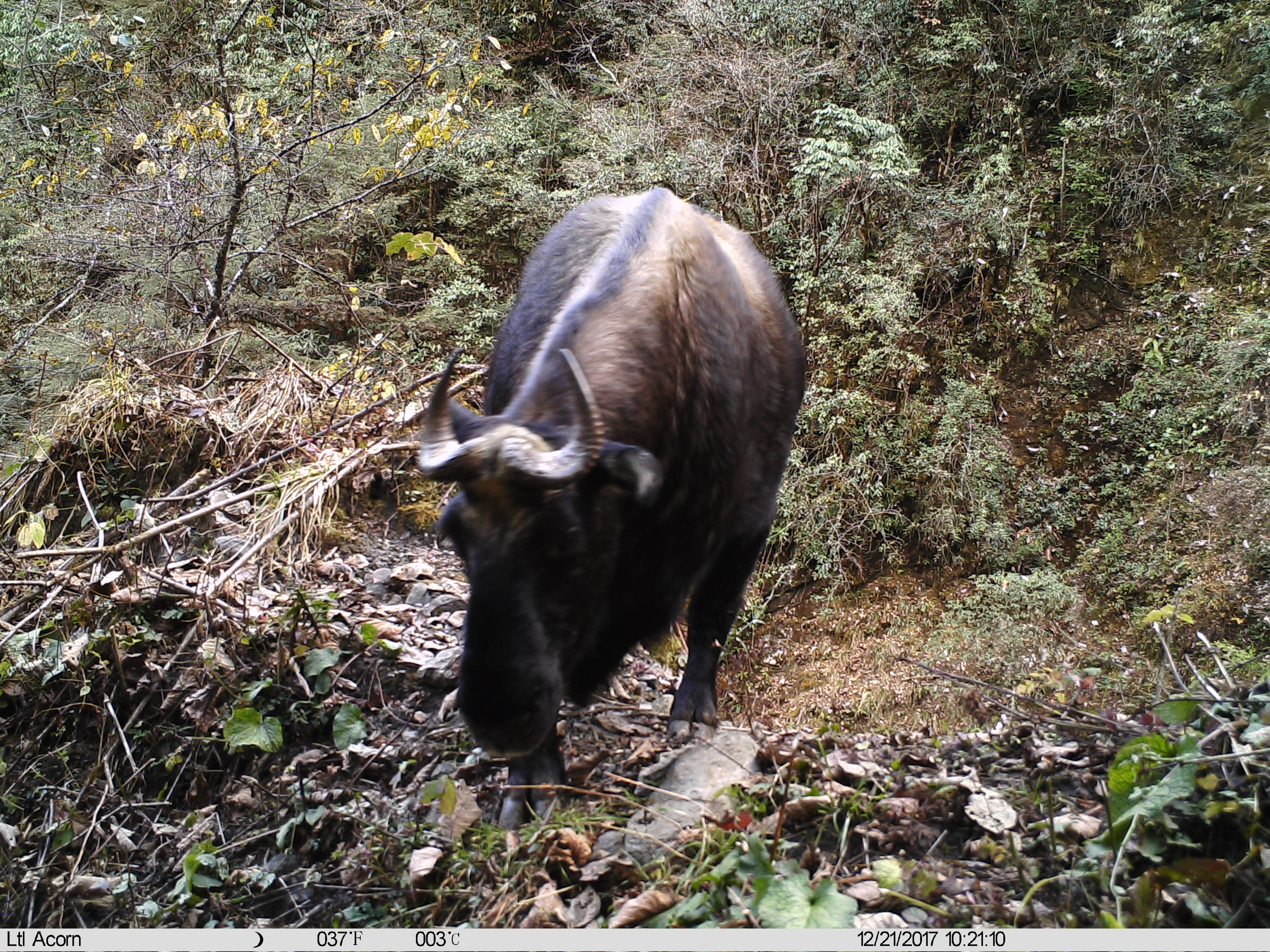

Supplement: Supplementary file 1 [file animals-14-02426-s001.zip › Budorcas taxicolor taxicolor-Part of the photos/IMAG0286 (2).JPG]

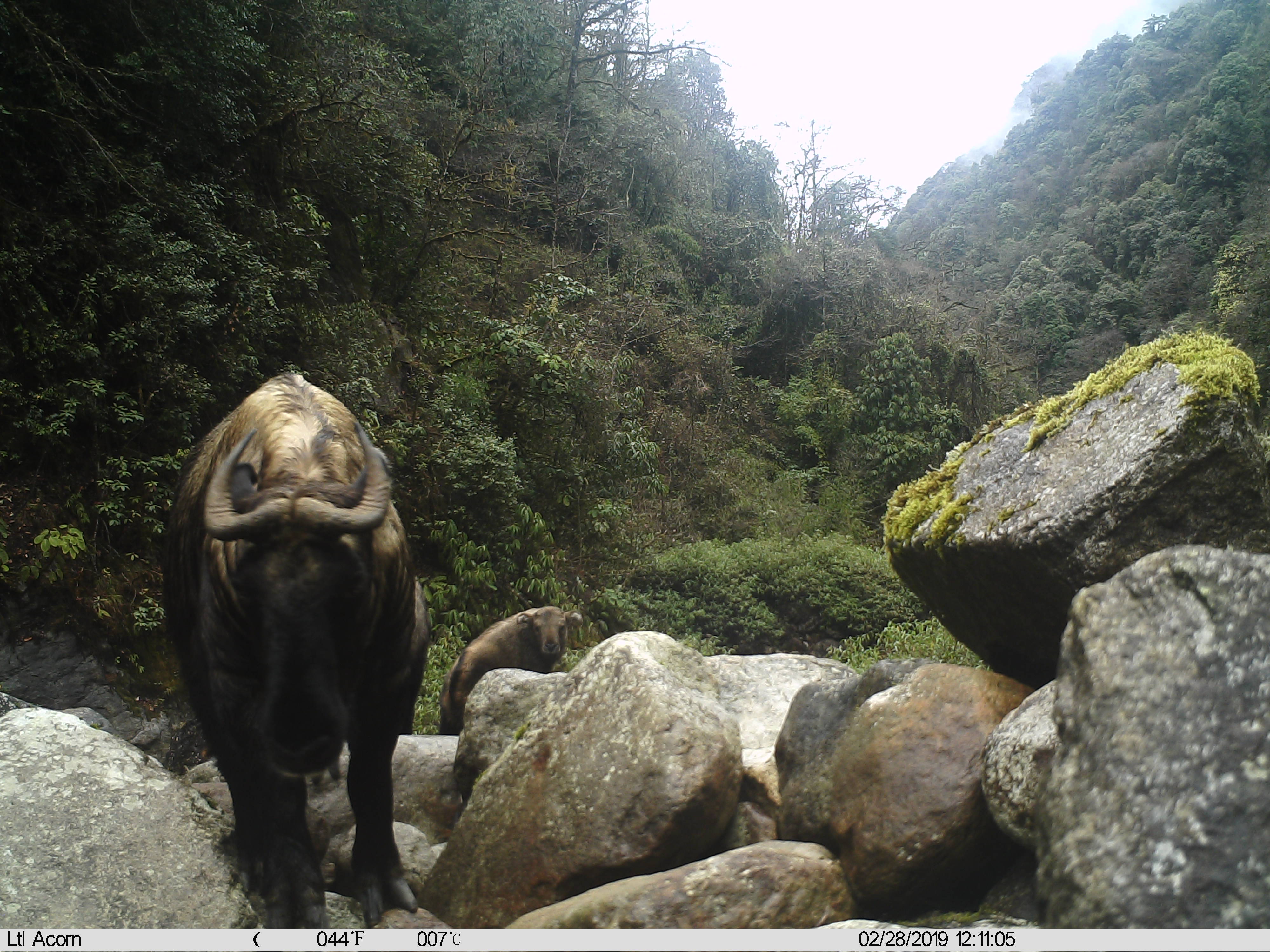

Supplement: Supplementary file 1 [file animals-14-02426-s001.zip › Budorcas taxicolor taxicolor-Part of the photos/IMAG0291.JPG]

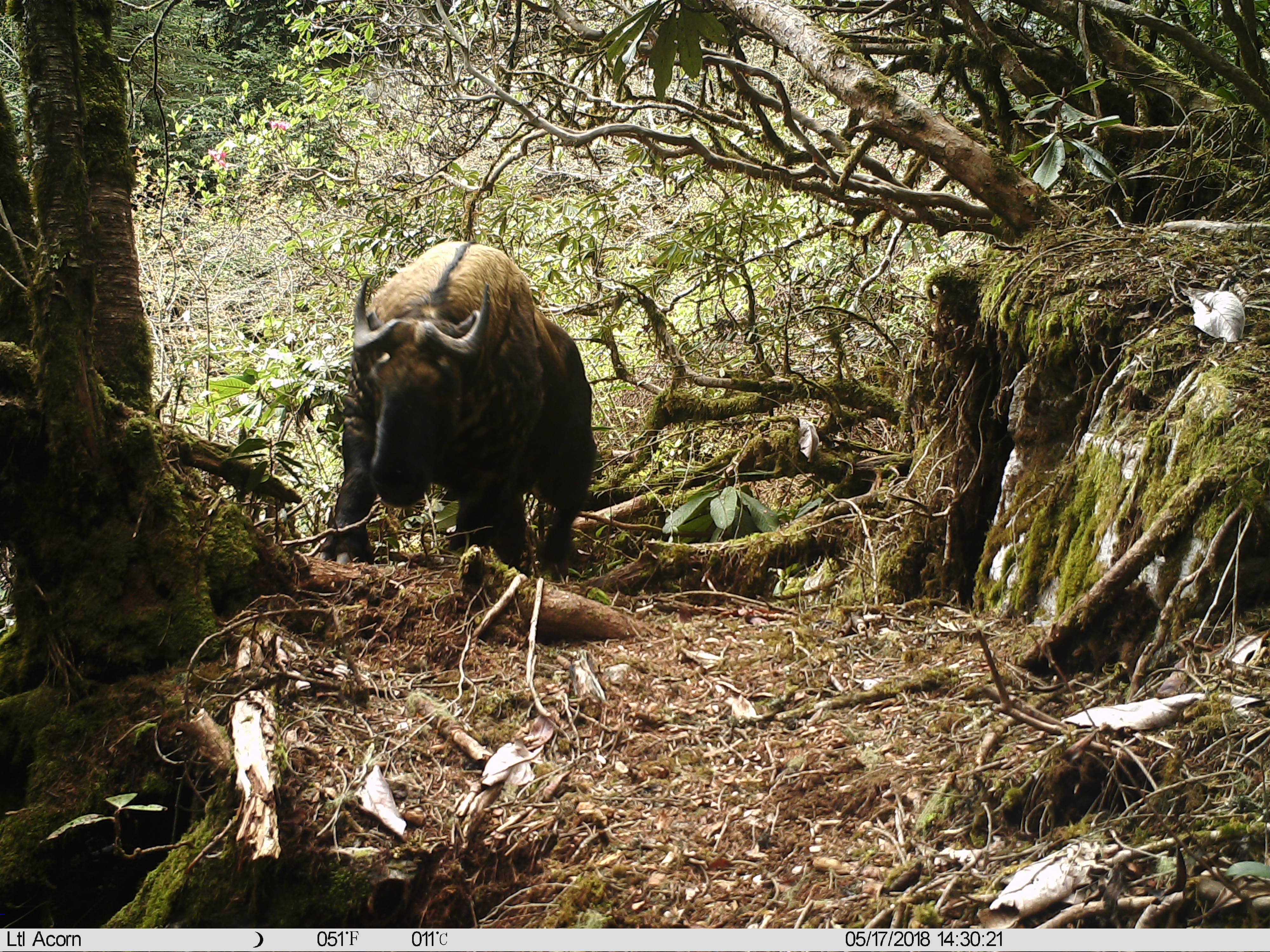

Supplement: Supplementary file 1 [file animals-14-02426-s001.zip › Budorcas taxicolor taxicolor-Part of the photos/IMAG0294 (2).JPG]

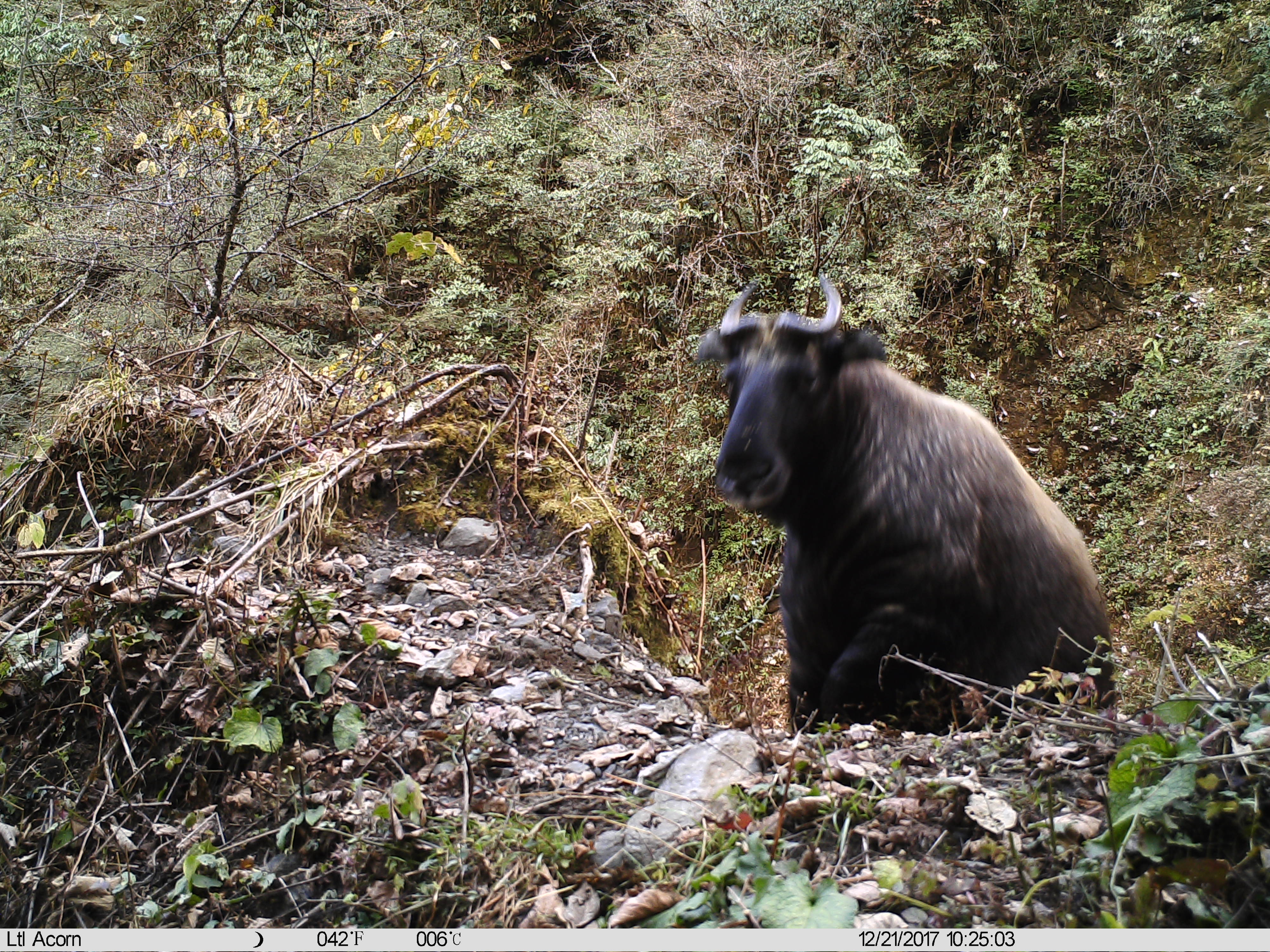

Supplement: Supplementary file 1 [file animals-14-02426-s001.zip › Budorcas taxicolor taxicolor-Part of the photos/IMAG0301 (2).JPG]

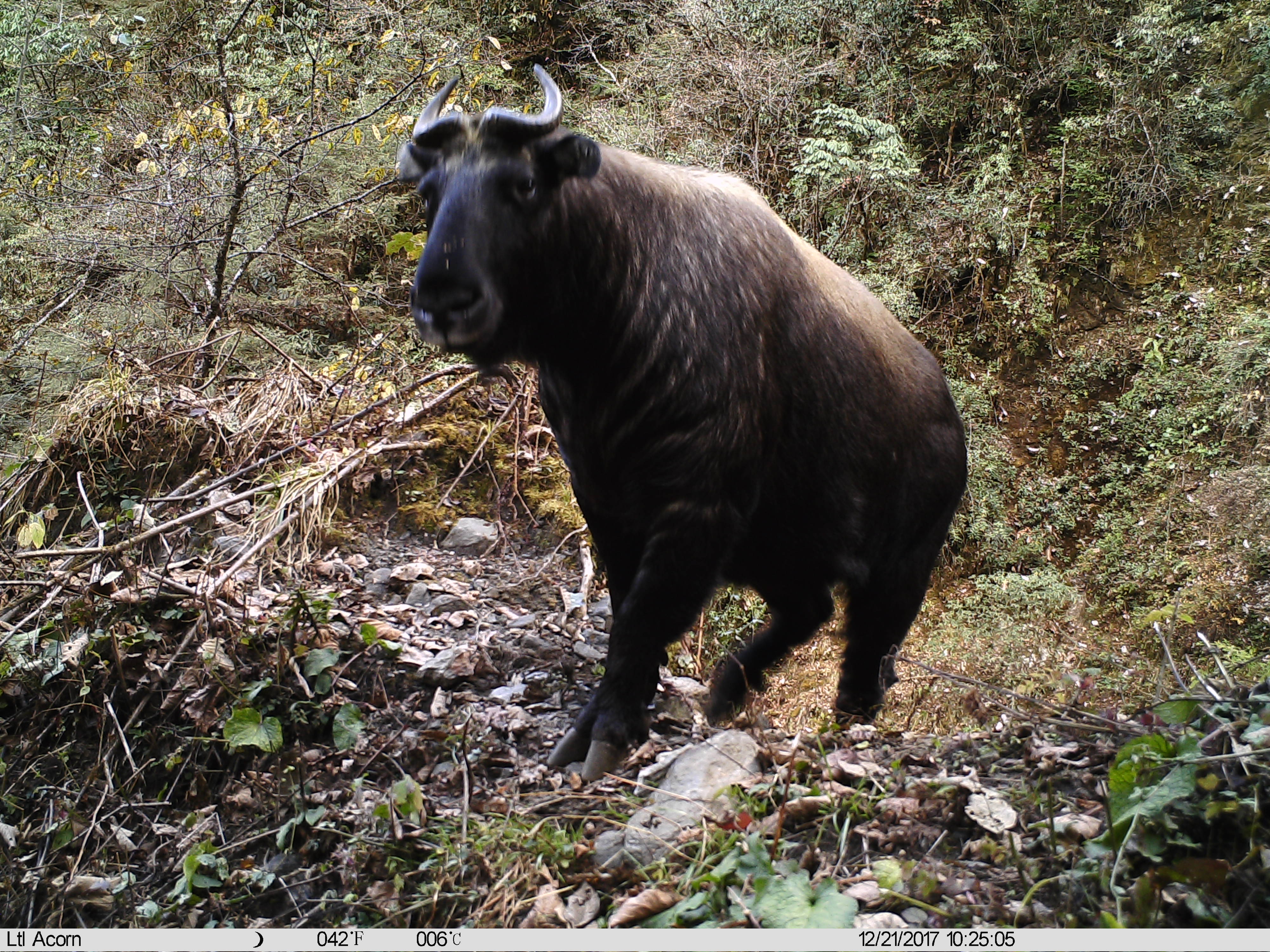

Supplement: Supplementary file 1 [file animals-14-02426-s001.zip › Budorcas taxicolor taxicolor-Part of the photos/IMAG0302 (2).JPG]

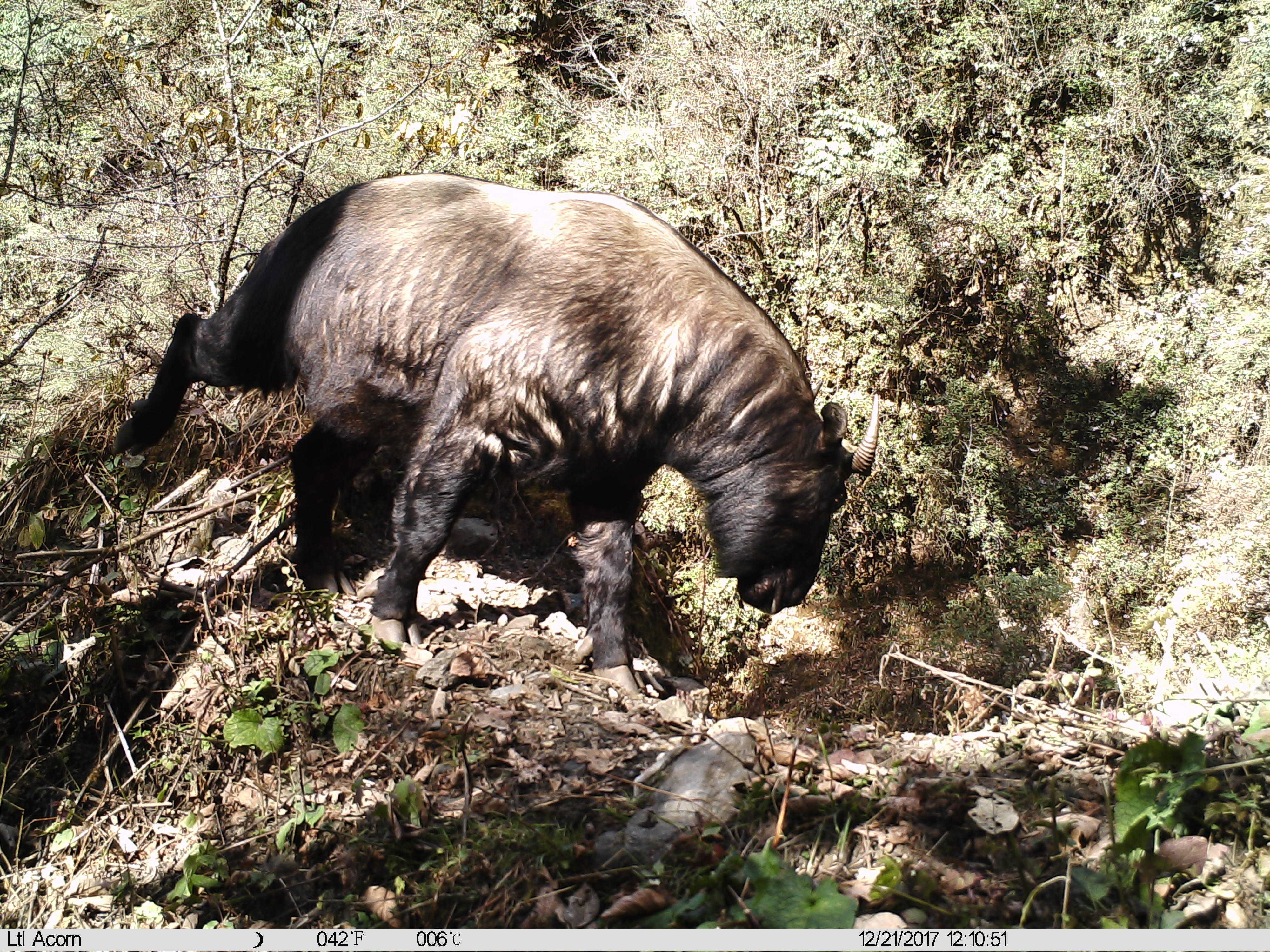

Supplement: Supplementary file 1 [file animals-14-02426-s001.zip › Budorcas taxicolor taxicolor-Part of the photos/IMAG0307 (2).JPG]

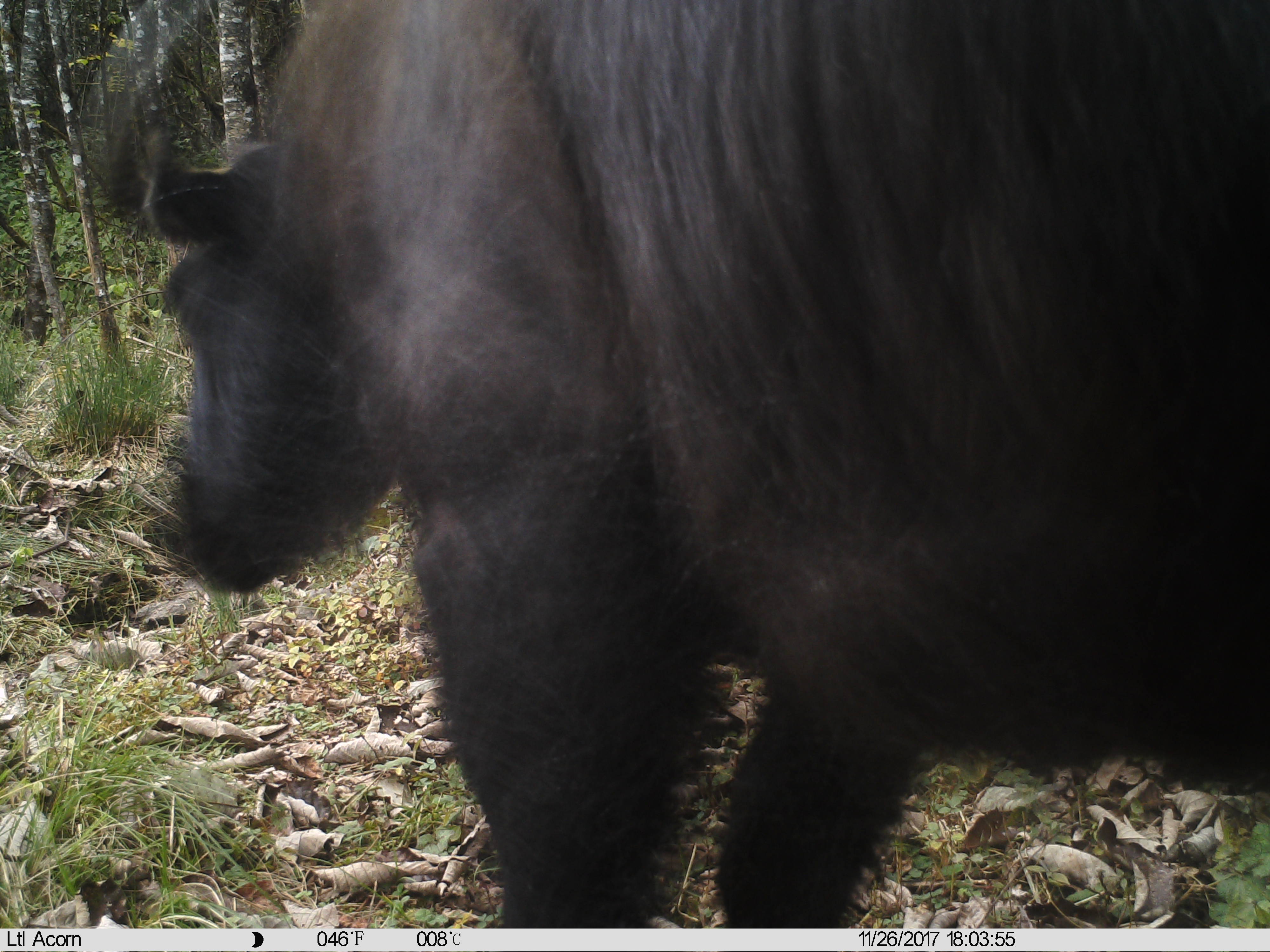

Supplement: Supplementary file 1 [file animals-14-02426-s001.zip › Budorcas taxicolor taxicolor-Part of the photos/IMAG0374.JPG]

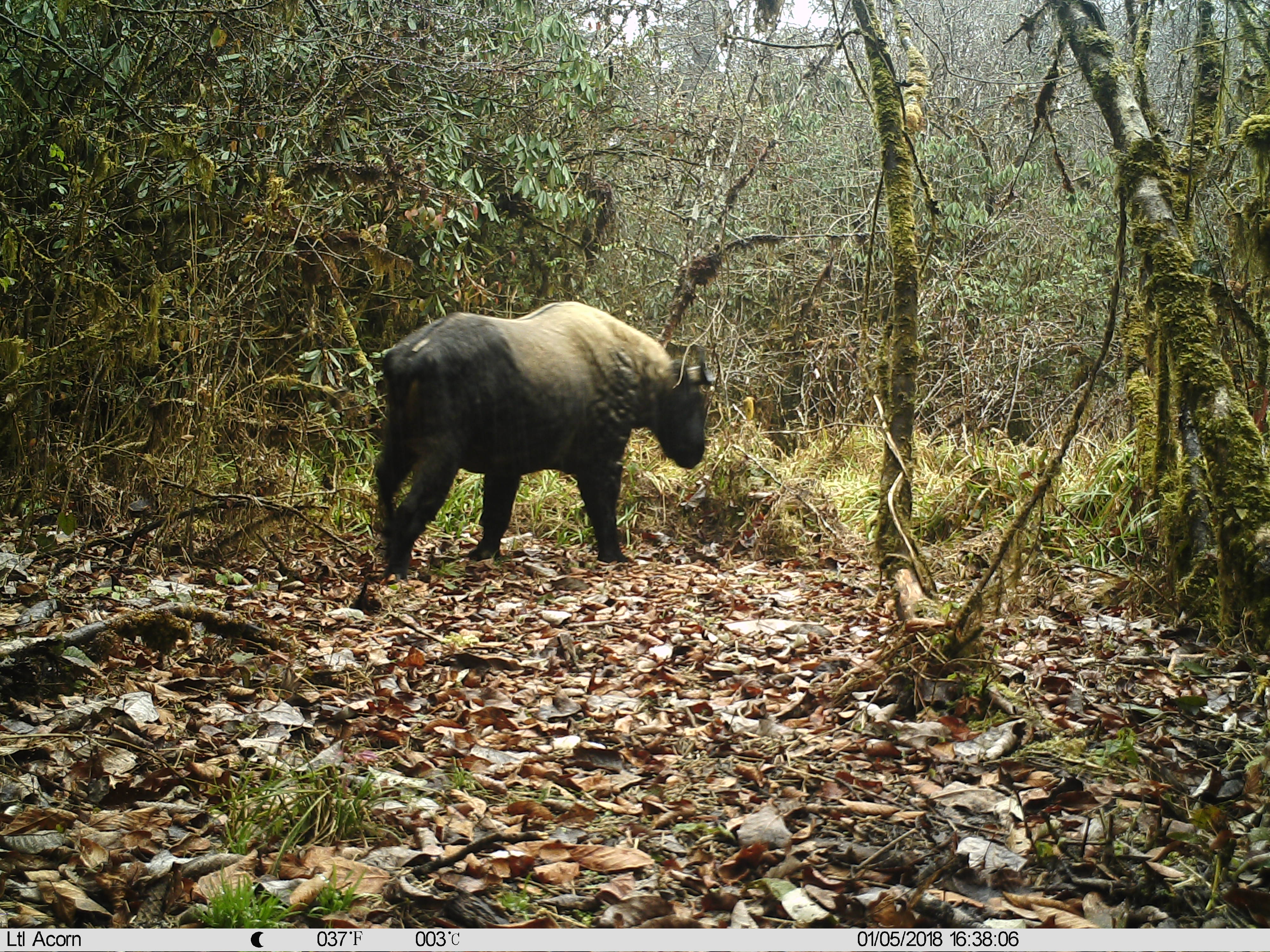

Supplement: Supplementary file 1 [file animals-14-02426-s001.zip › Budorcas taxicolor taxicolor-Part of the photos/IMAG0385 (2).JPG]

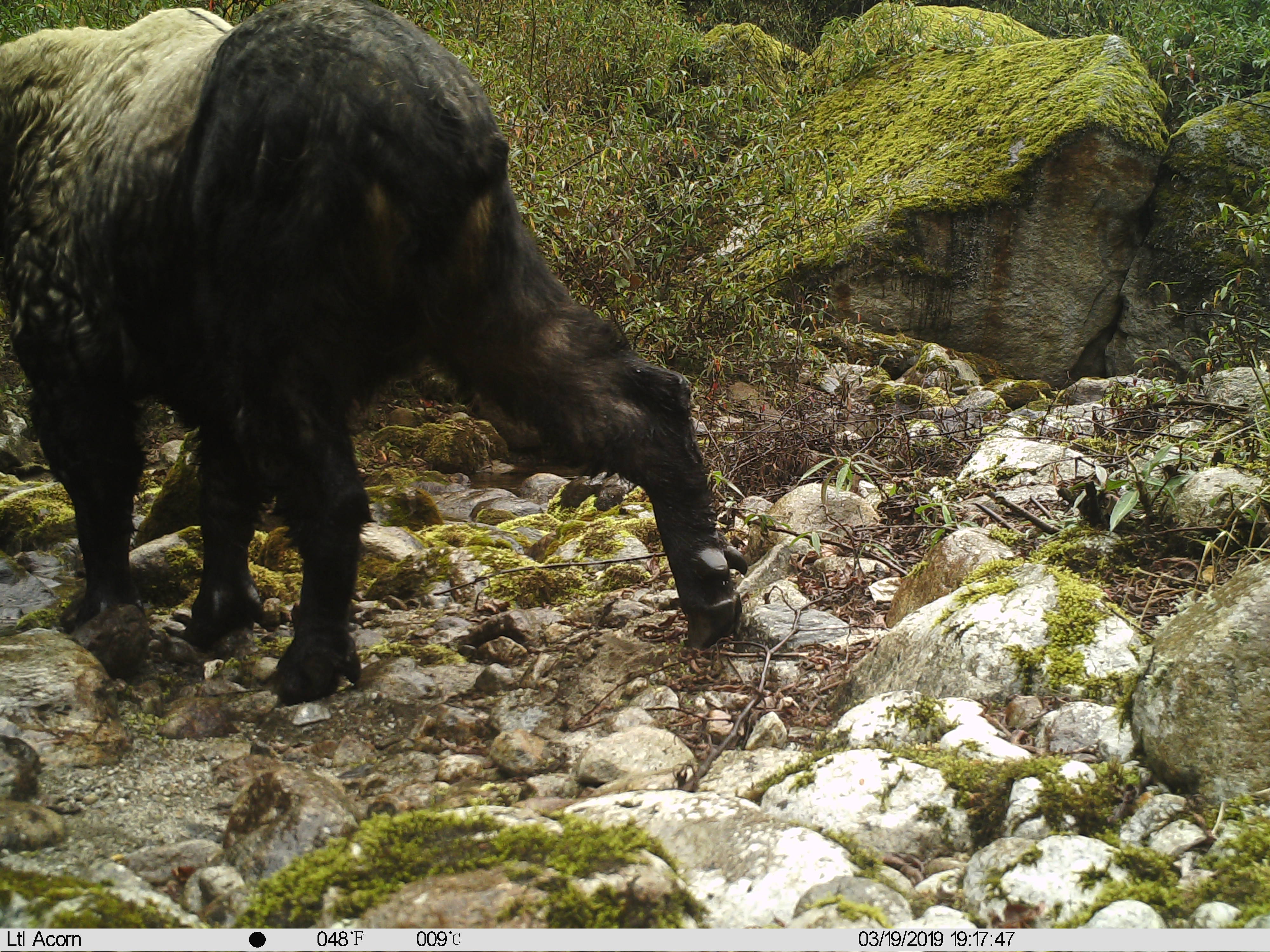

Supplement: Supplementary file 1 [file animals-14-02426-s001.zip › Budorcas taxicolor taxicolor-Part of the photos/IMAG0438.JPG]

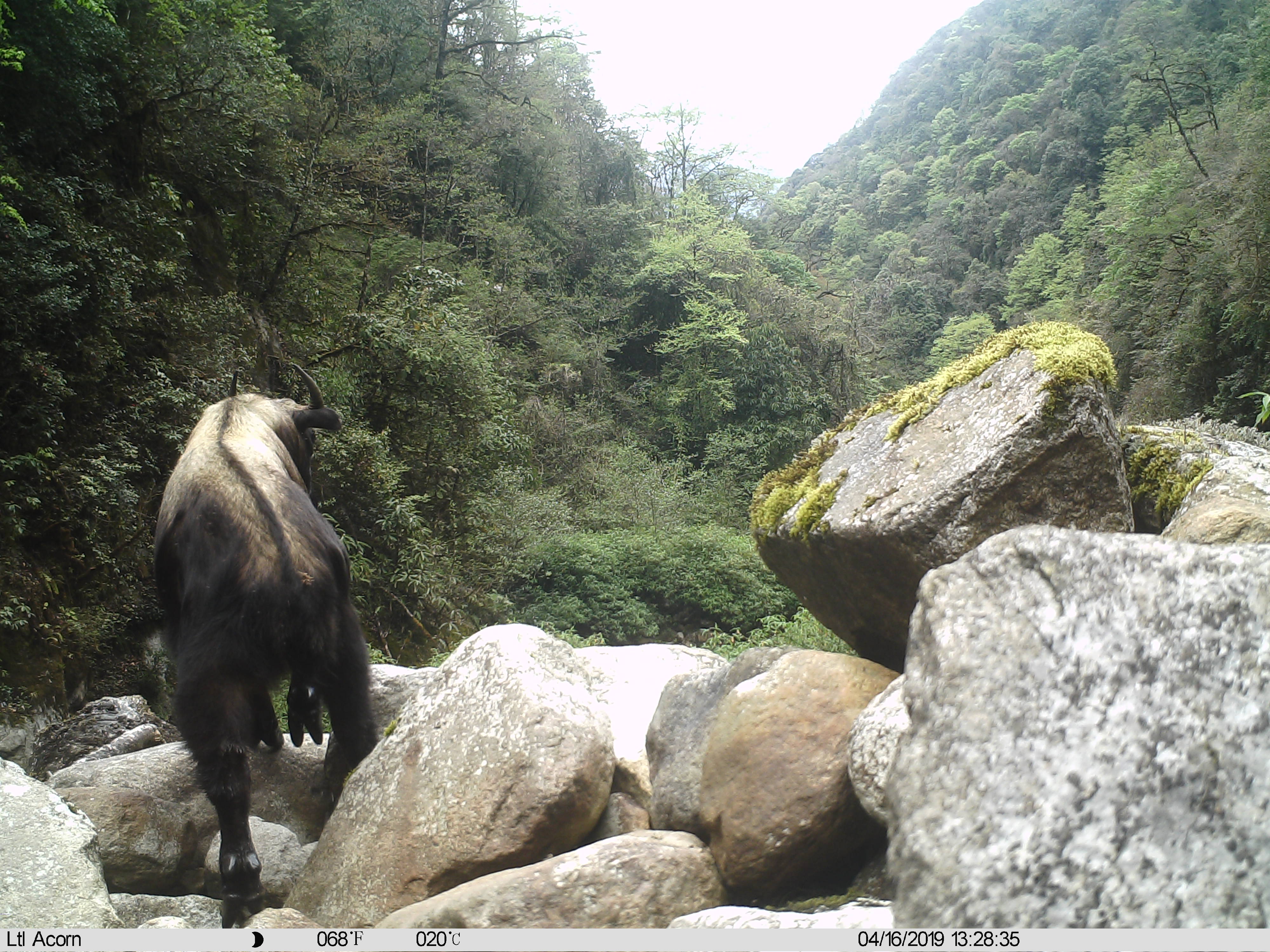

Supplement: Supplementary file 1 [file animals-14-02426-s001.zip › Budorcas taxicolor taxicolor-Part of the photos/IMAG0449.JPG]

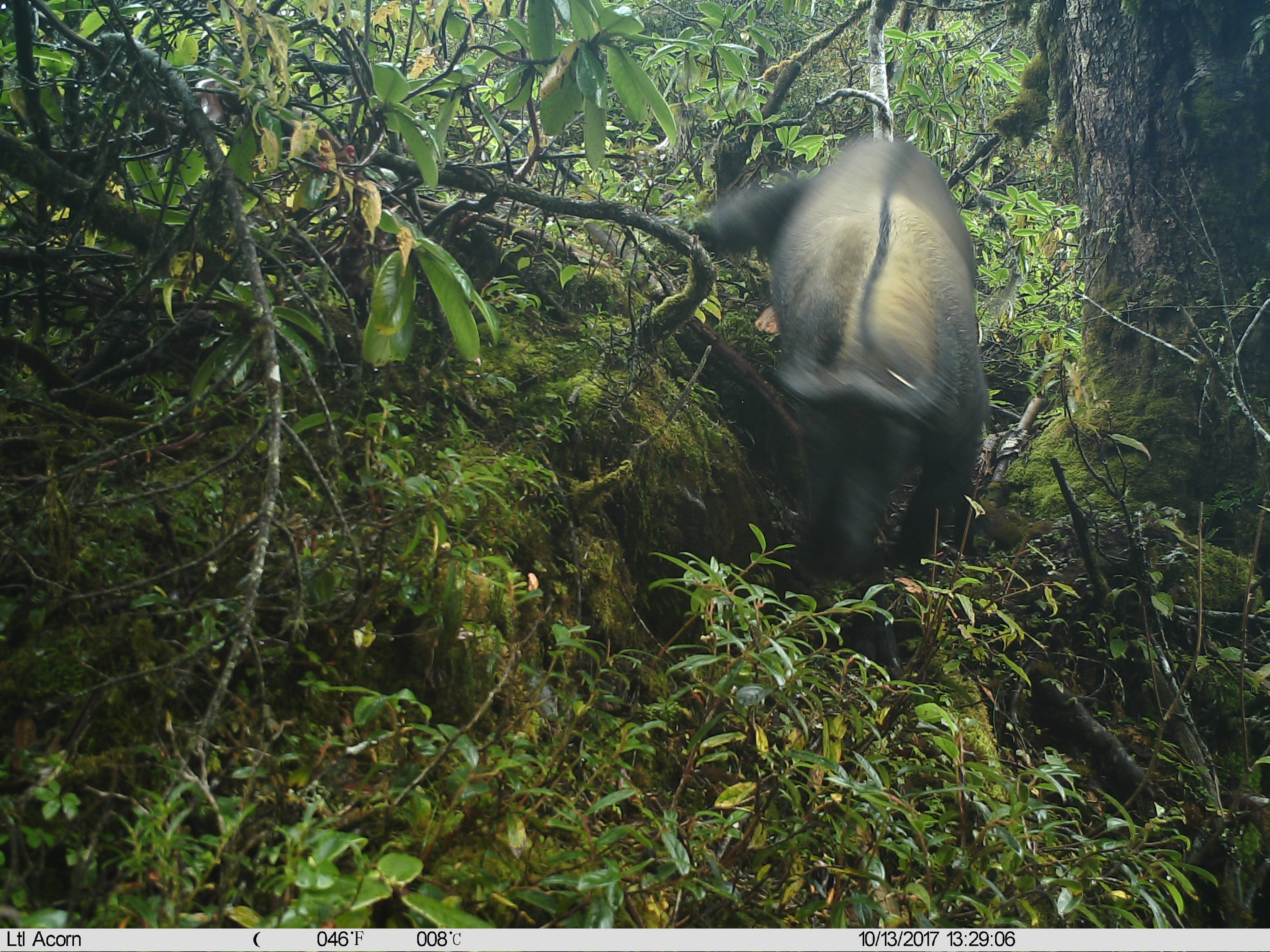

Supplement: Supplementary file 1 [file animals-14-02426-s001.zip › Budorcas taxicolor taxicolor-Part of the photos/IMAG0497.JPG]

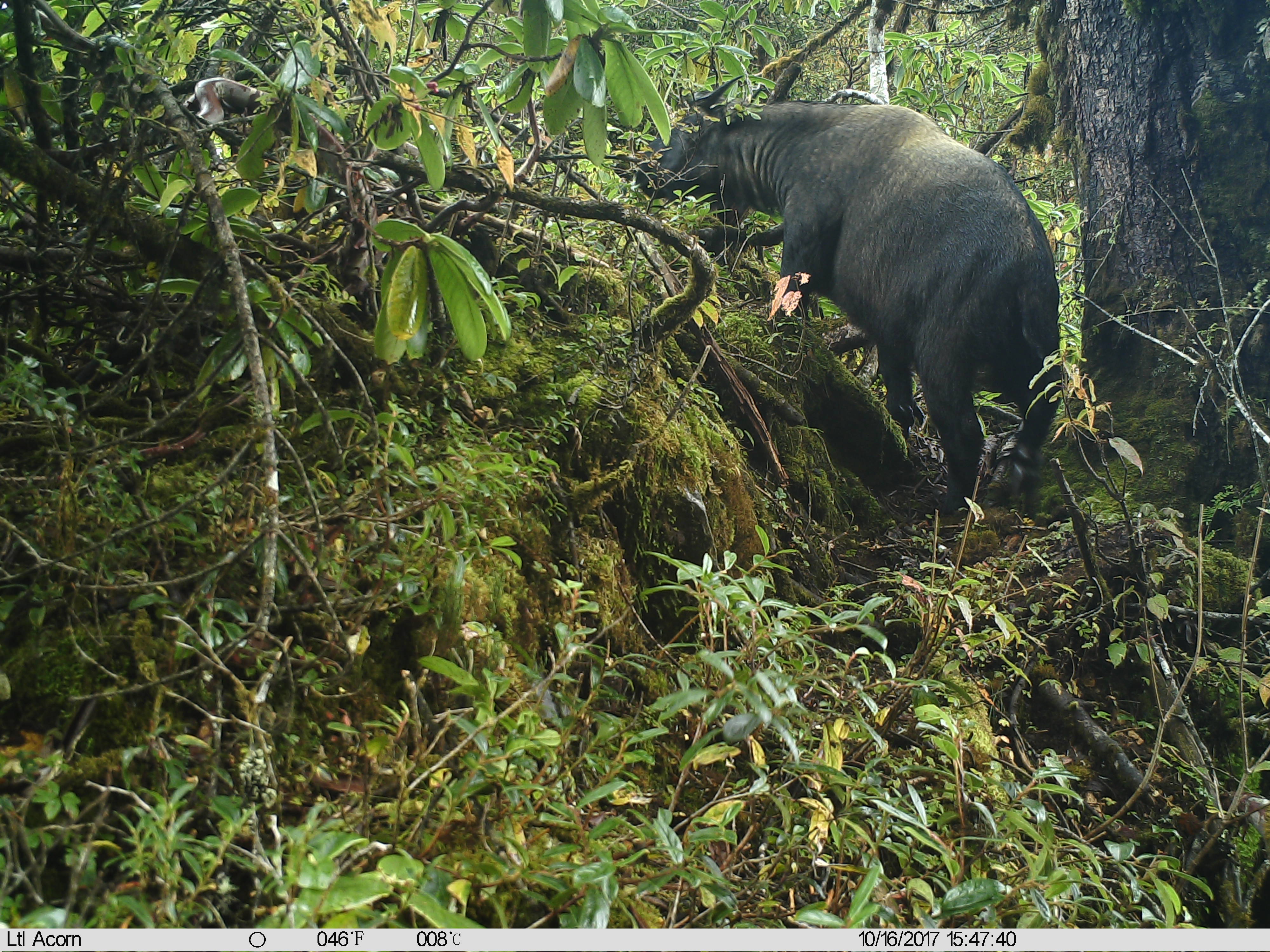

Supplement: Supplementary file 1 [file animals-14-02426-s001.zip › Budorcas taxicolor taxicolor-Part of the photos/IMAG0513.JPG]

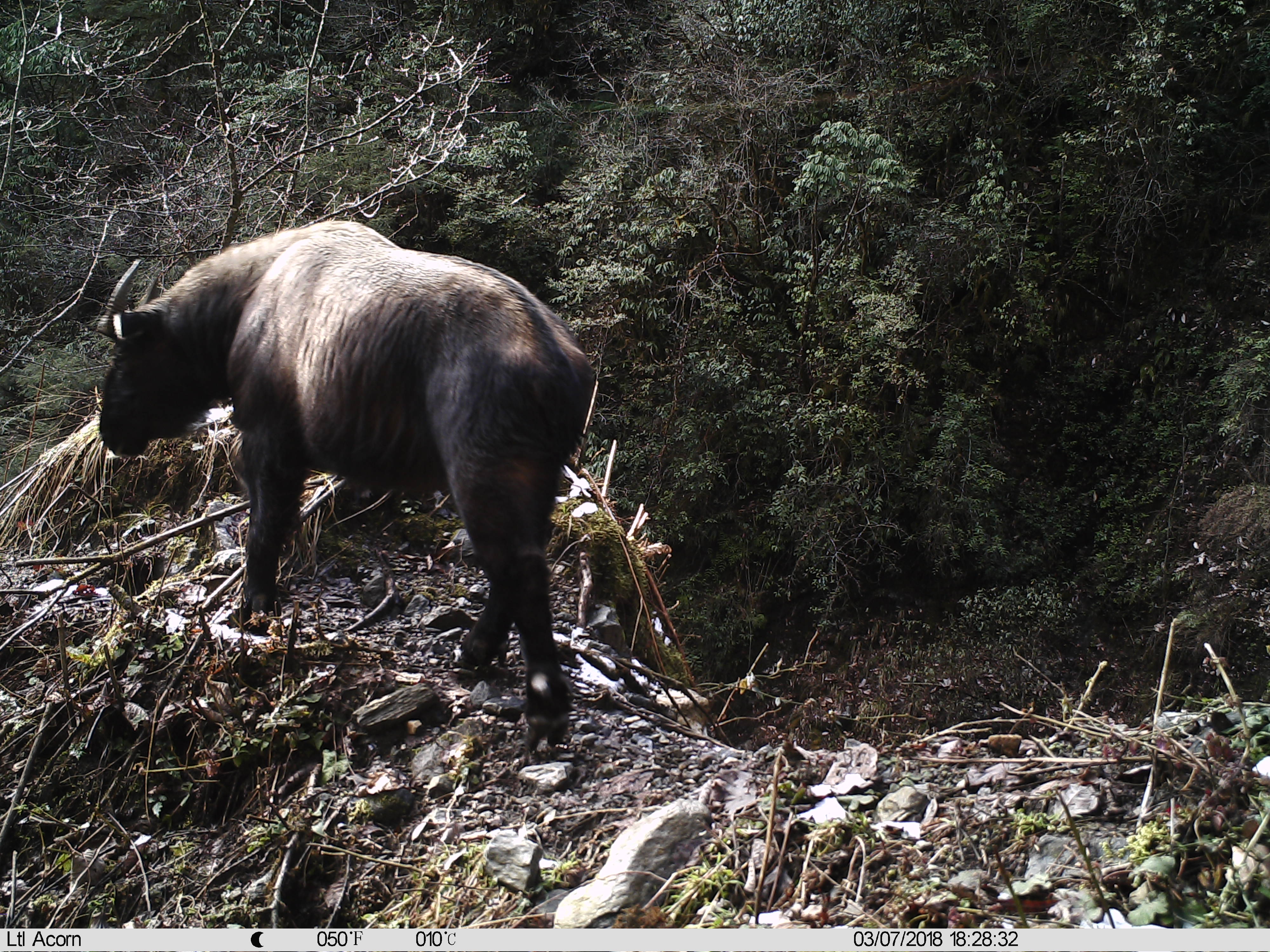

Supplement: Supplementary file 1 [file animals-14-02426-s001.zip › Budorcas taxicolor taxicolor-Part of the photos/IMAG0579 (2).JPG]

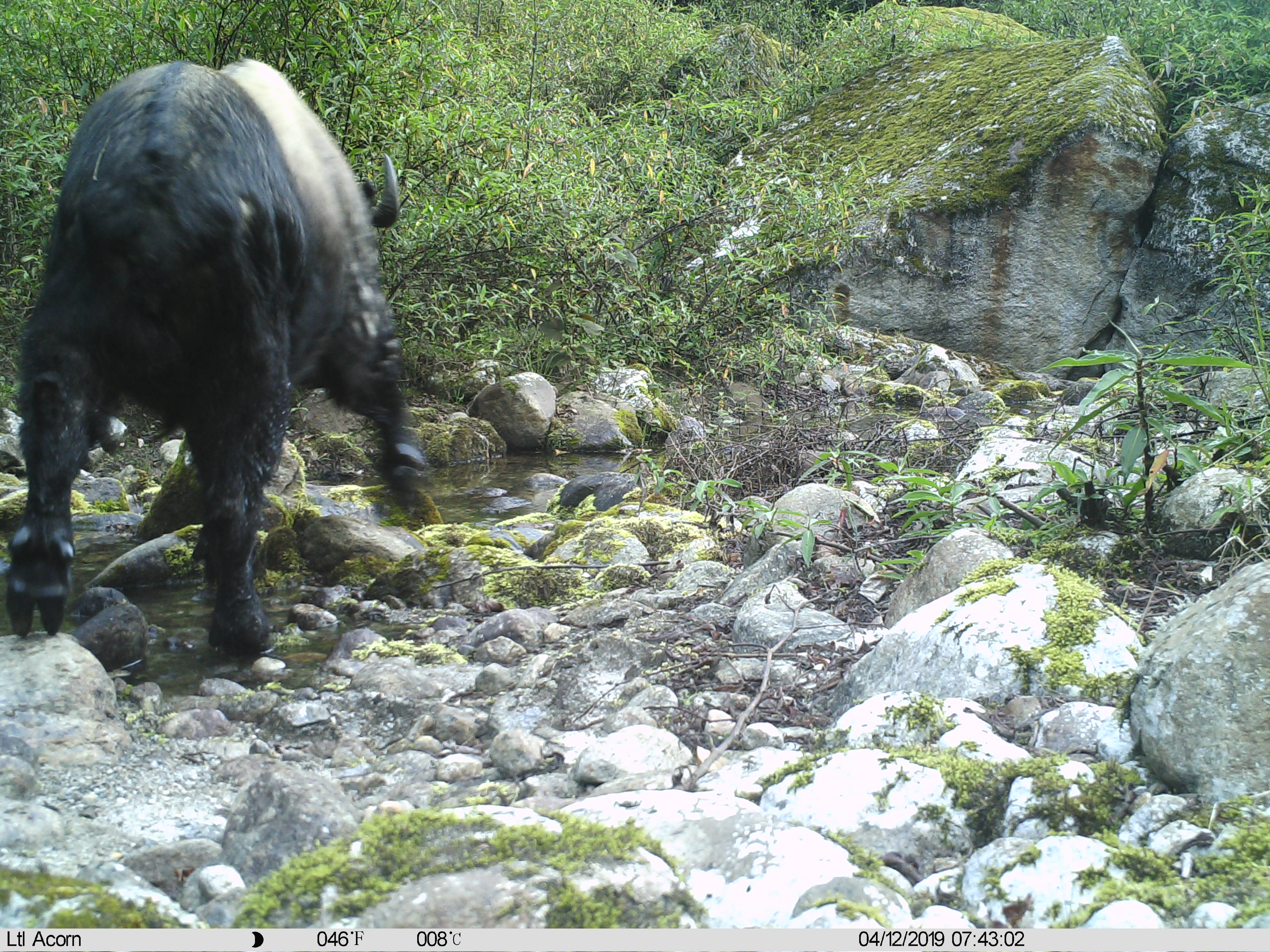

Supplement: Supplementary file 1 [file animals-14-02426-s001.zip › Budorcas taxicolor taxicolor-Part of the photos/IMAG0675.JPG]

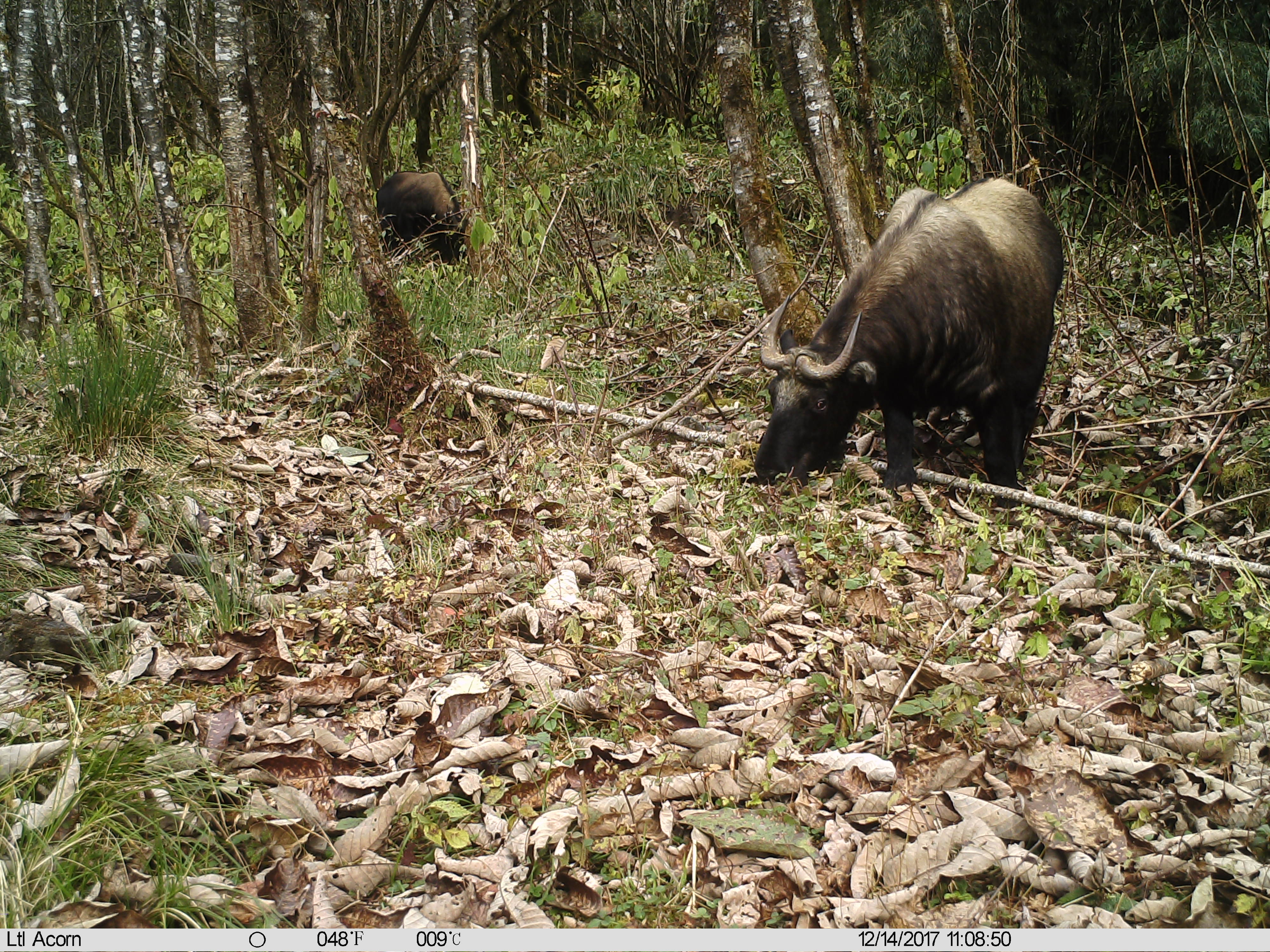

Supplement: Supplementary file 1 [file animals-14-02426-s001.zip › Budorcas taxicolor taxicolor-Part of the photos/IMAG0753 (2).JPG]

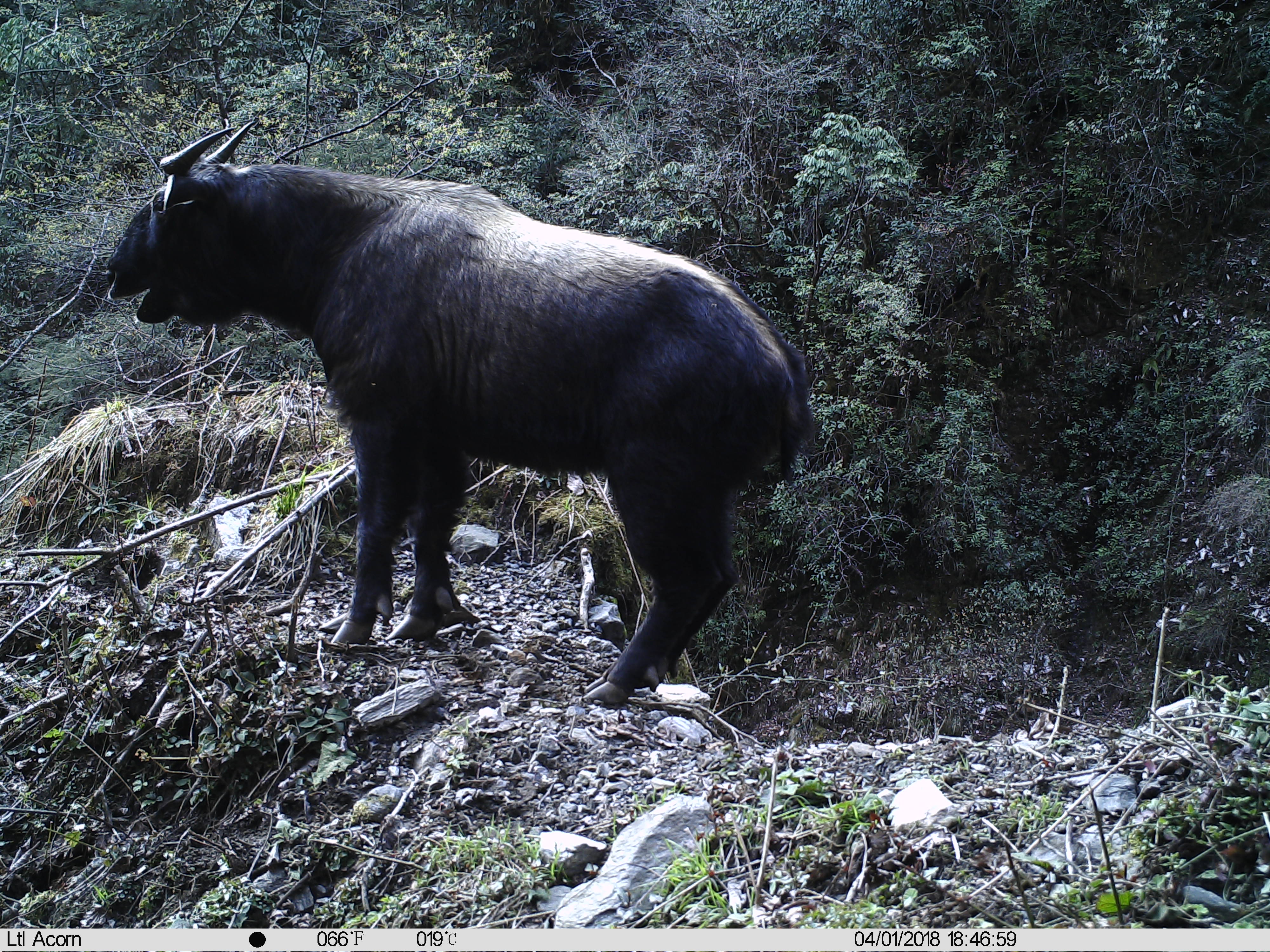

Supplement: Supplementary file 1 [file animals-14-02426-s001.zip › Budorcas taxicolor taxicolor-Part of the photos/IMAG0783.JPG]

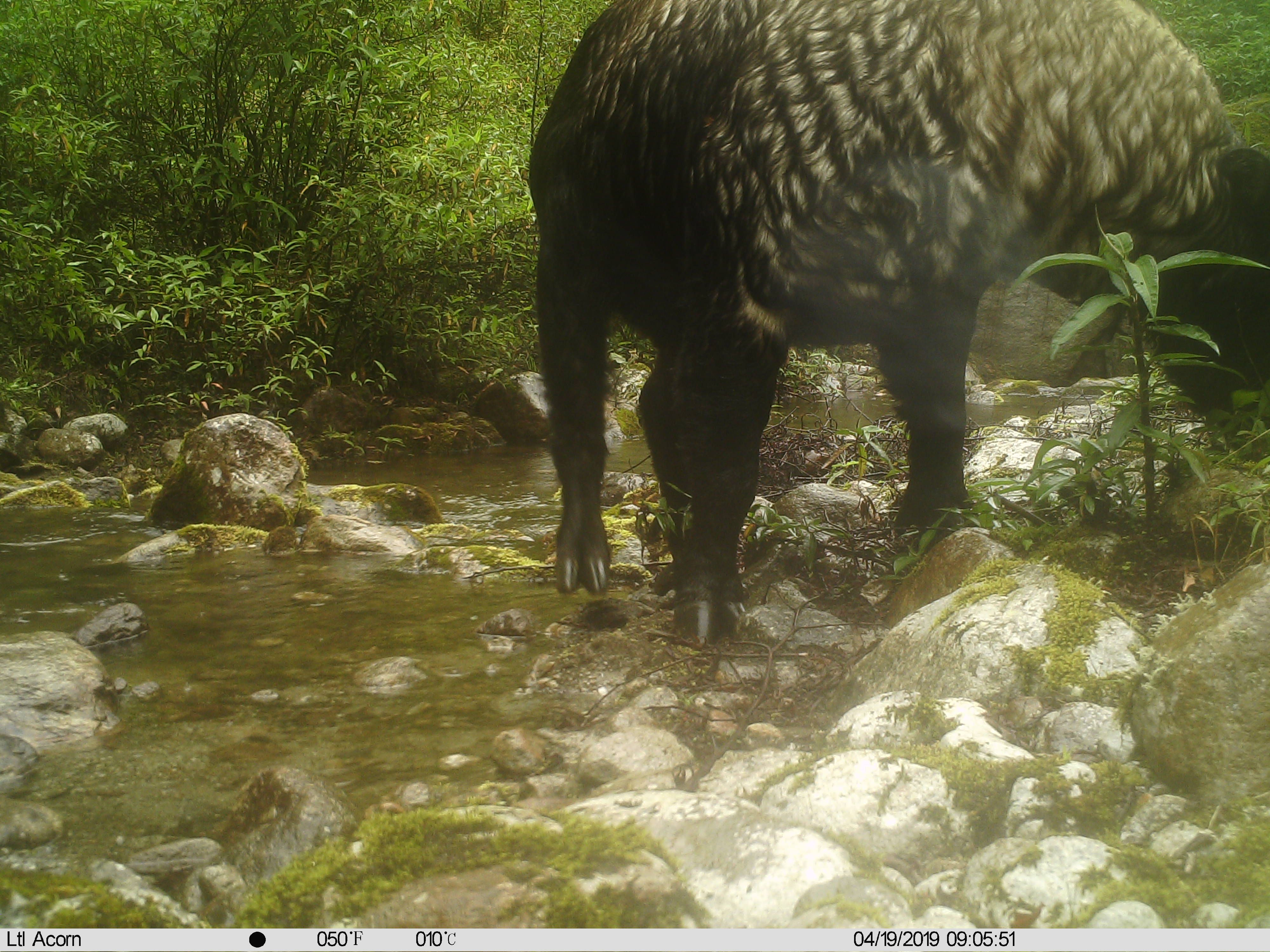

Supplement: Supplementary file 1 [file animals-14-02426-s001.zip › Budorcas taxicolor taxicolor-Part of the photos/IMAG0822.JPG]

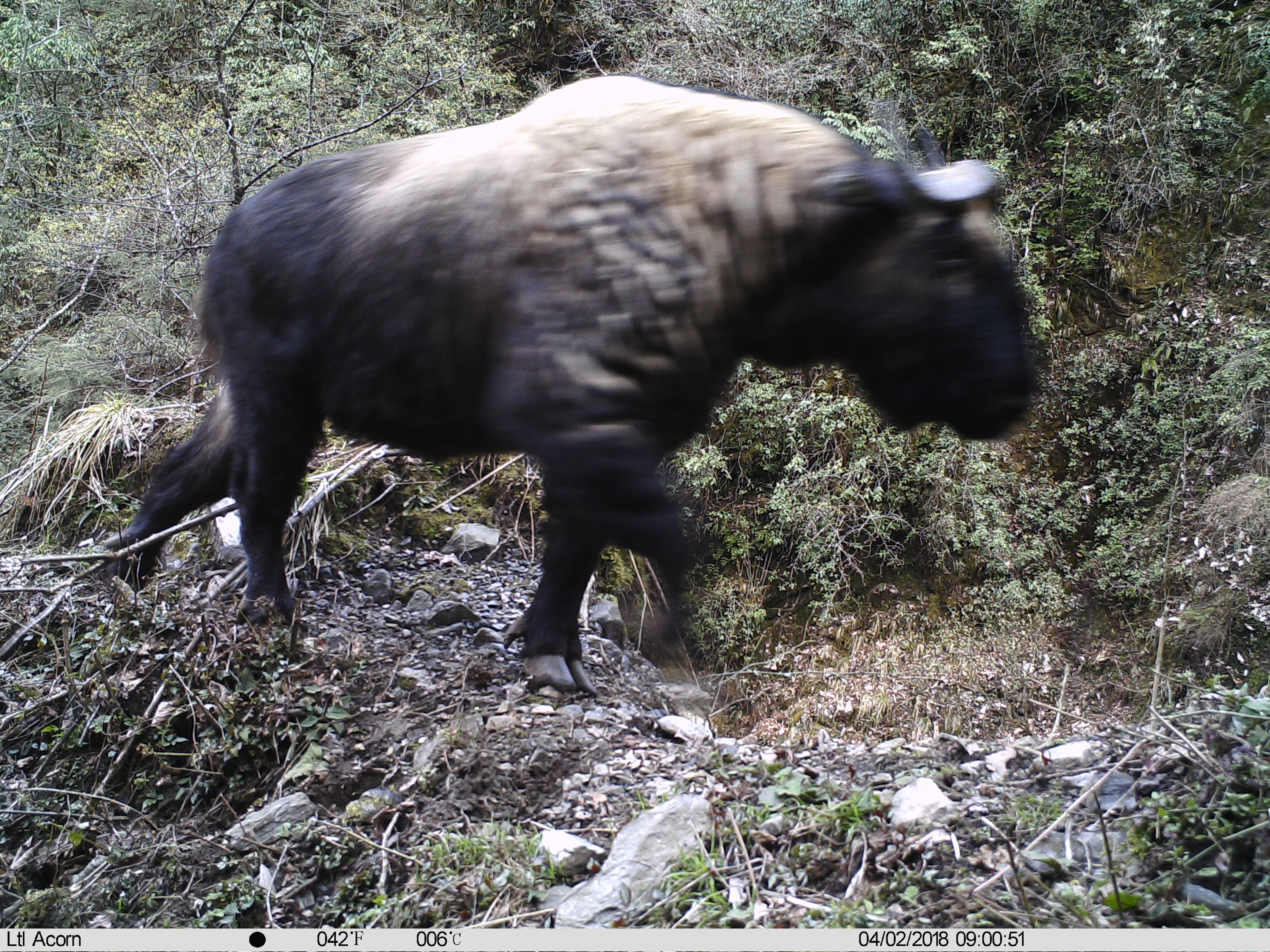

Supplement: Supplementary file 1 [file animals-14-02426-s001.zip › Budorcas taxicolor taxicolor-Part of the photos/IMAG0834 (2).JPG]

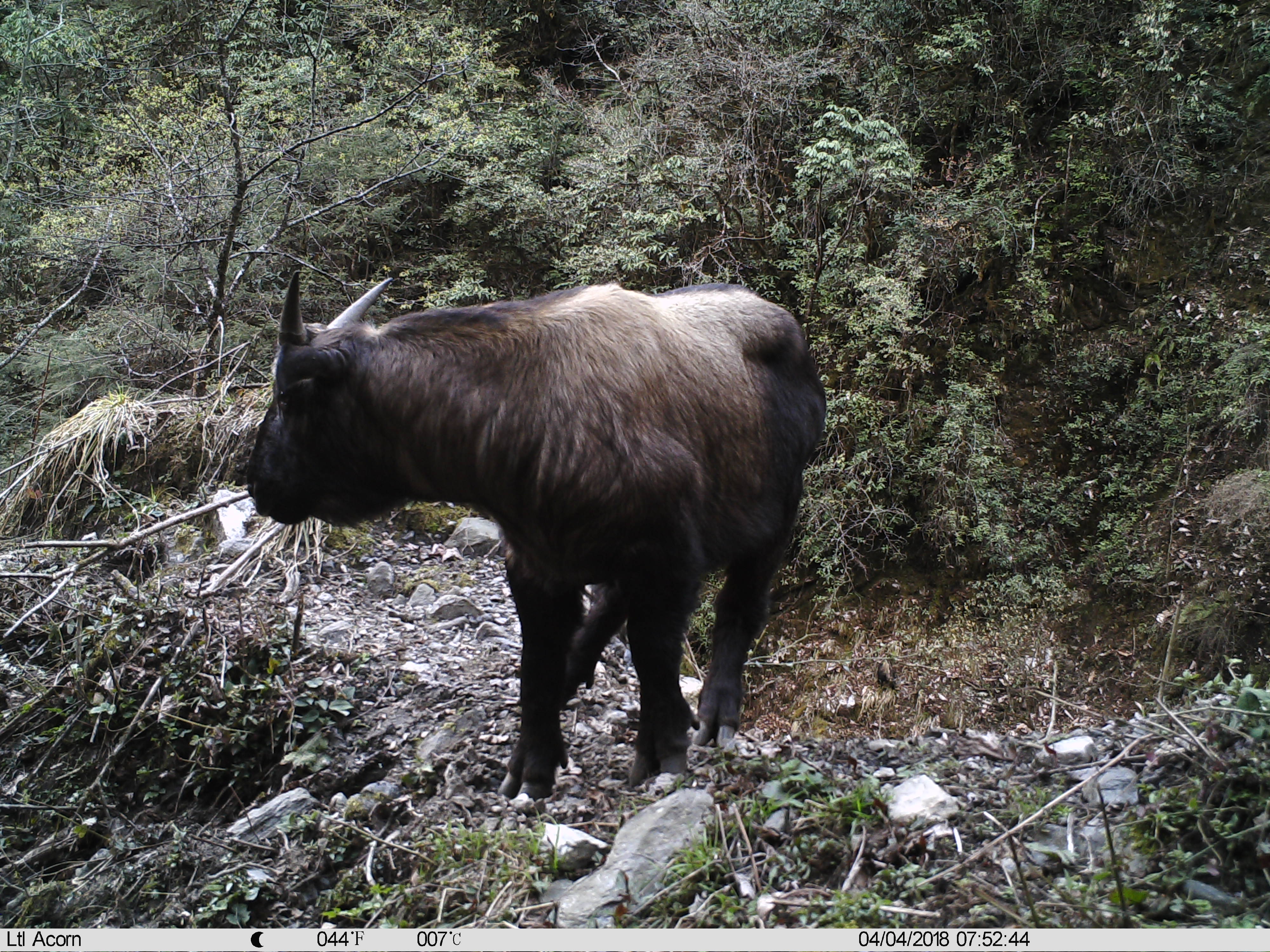

Supplement: Supplementary file 1 [file animals-14-02426-s001.zip › Budorcas taxicolor taxicolor-Part of the photos/IMAG0862 (2).JPG]

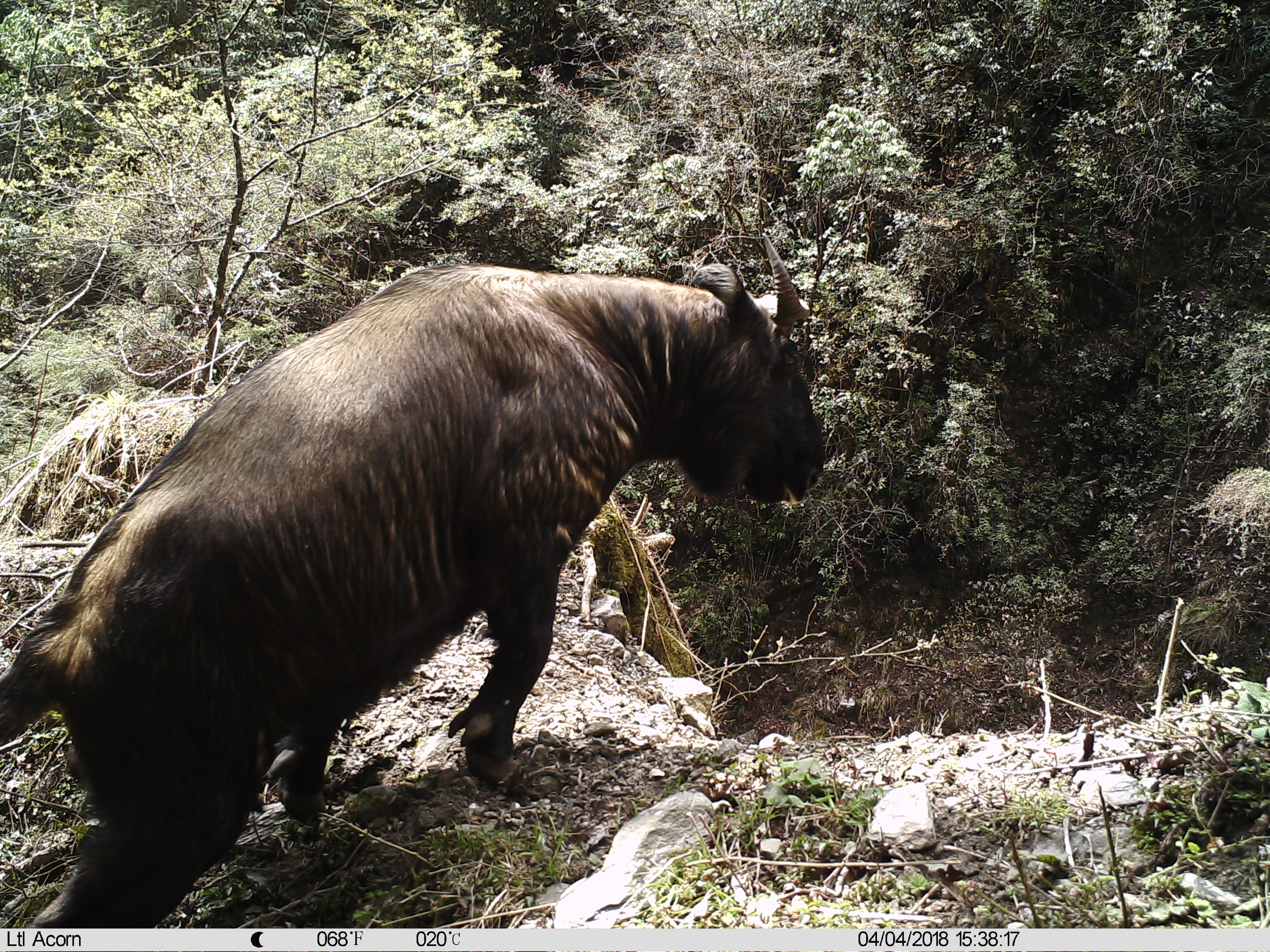

Supplement: Supplementary file 1 [file animals-14-02426-s001.zip › Budorcas taxicolor taxicolor-Part of the photos/IMAG0922.JPG]

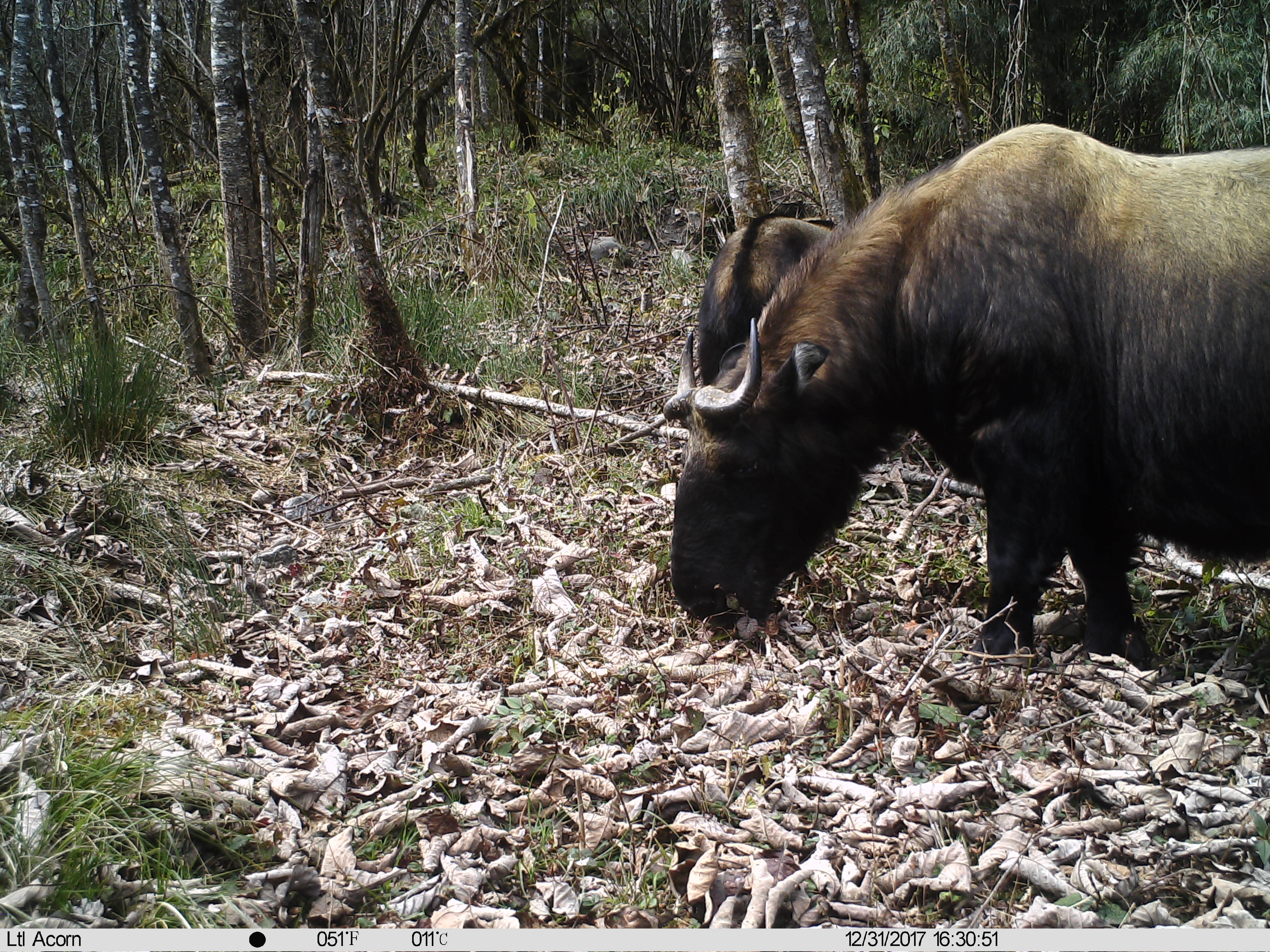

Supplement: Supplementary file 1 [file animals-14-02426-s001.zip › Budorcas taxicolor taxicolor-Part of the photos/IMAG1006 (2).JPG]

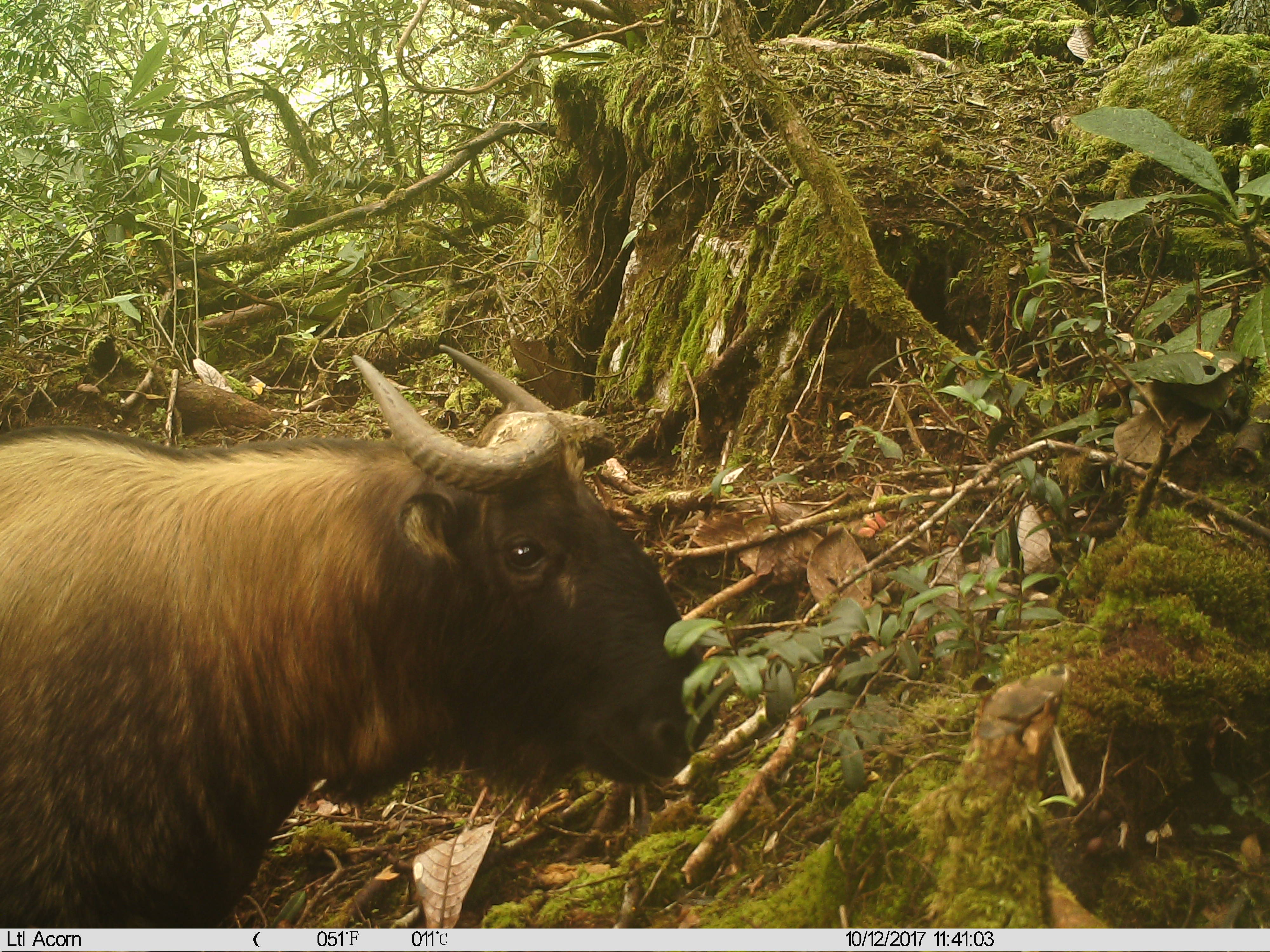

Supplement: Supplementary file 1 [file animals-14-02426-s001.zip › Budorcas taxicolor taxicolor-Part of the photos/IMAG1025.JPG]

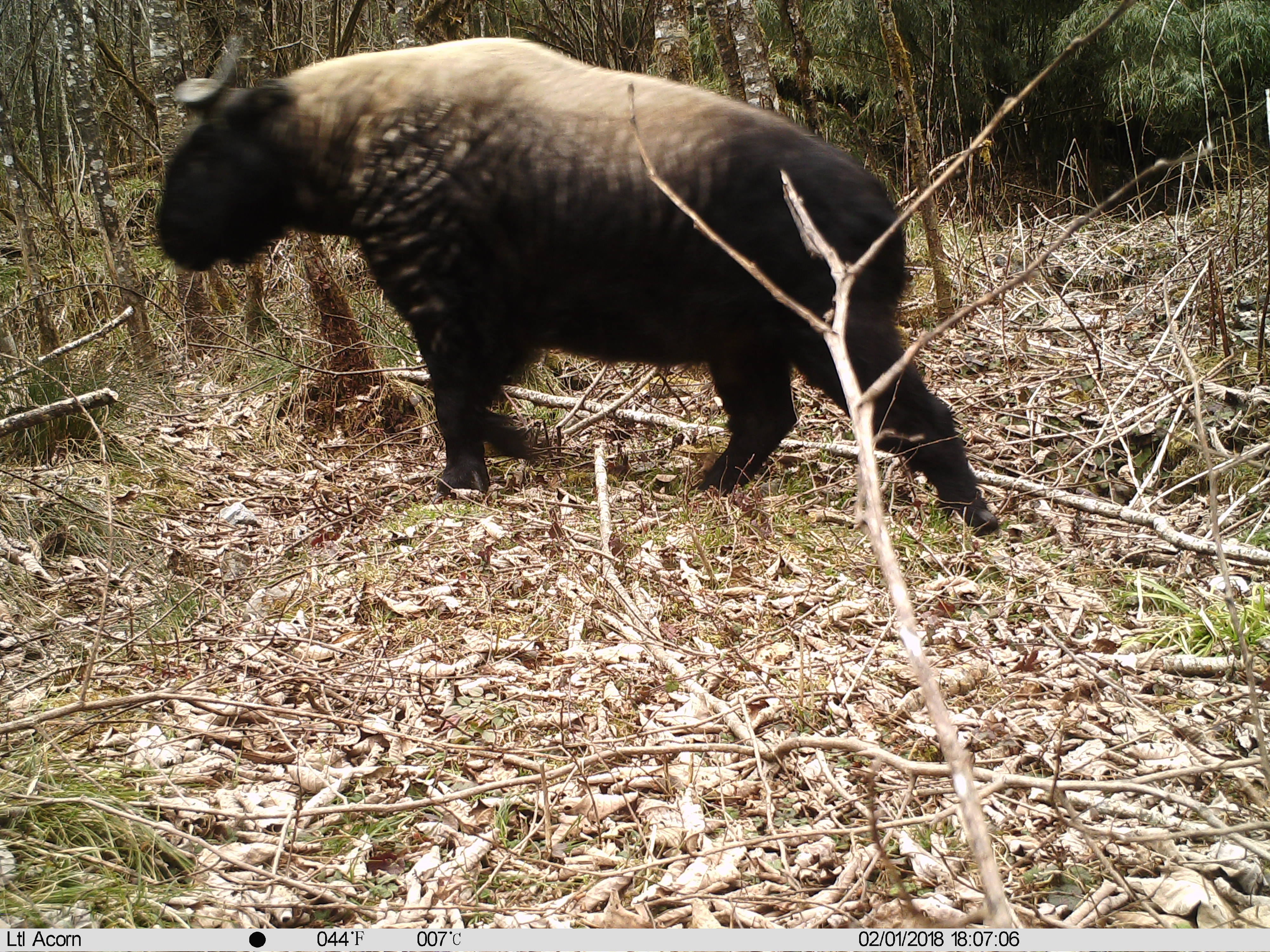

Supplement: Supplementary file 1 [file animals-14-02426-s001.zip › Budorcas taxicolor taxicolor-Part of the photos/IMAG1442.JPG]

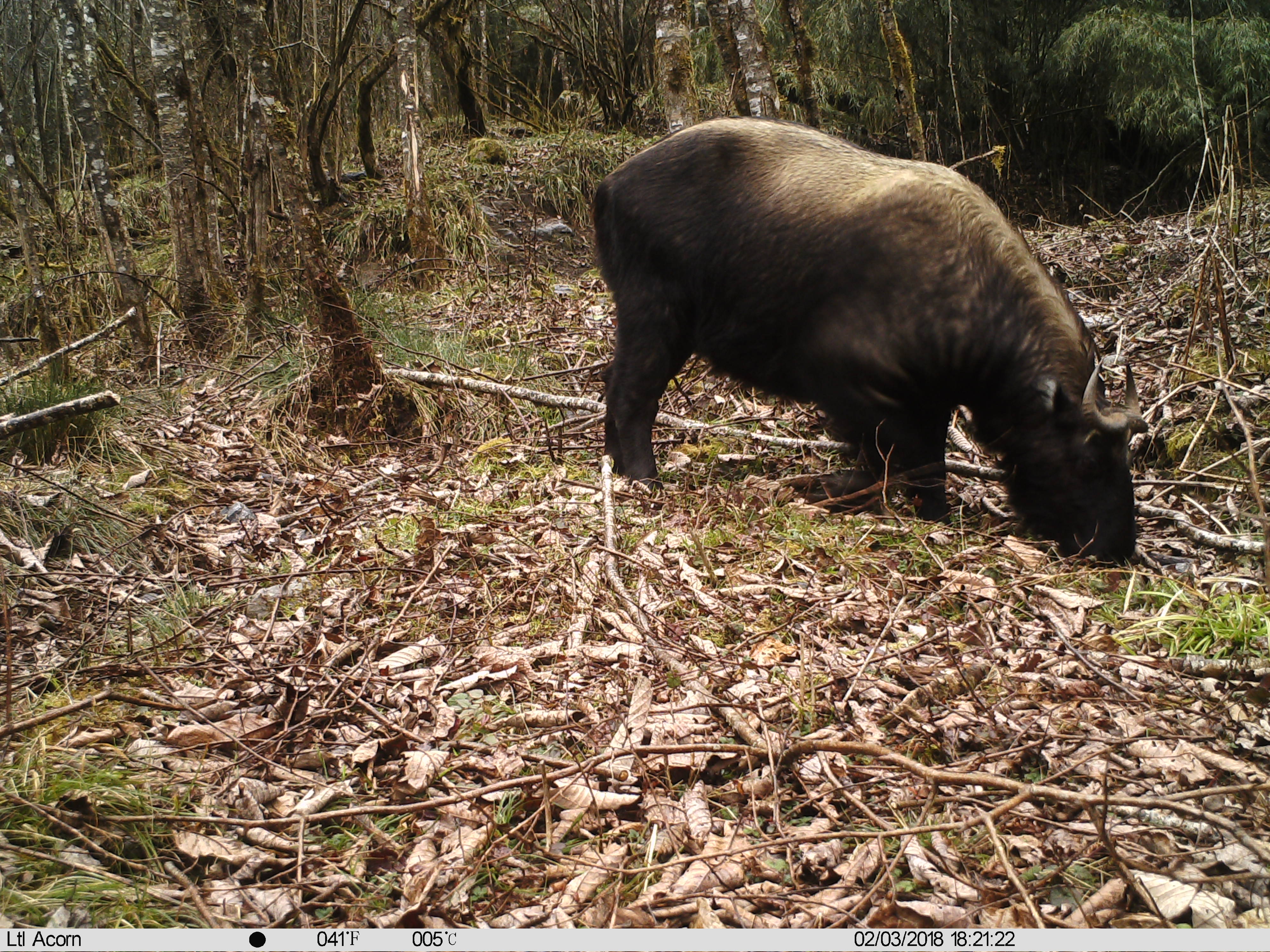

Supplement: Supplementary file 1 [file animals-14-02426-s001.zip › Budorcas taxicolor taxicolor-Part of the photos/IMAG1499.JPG]

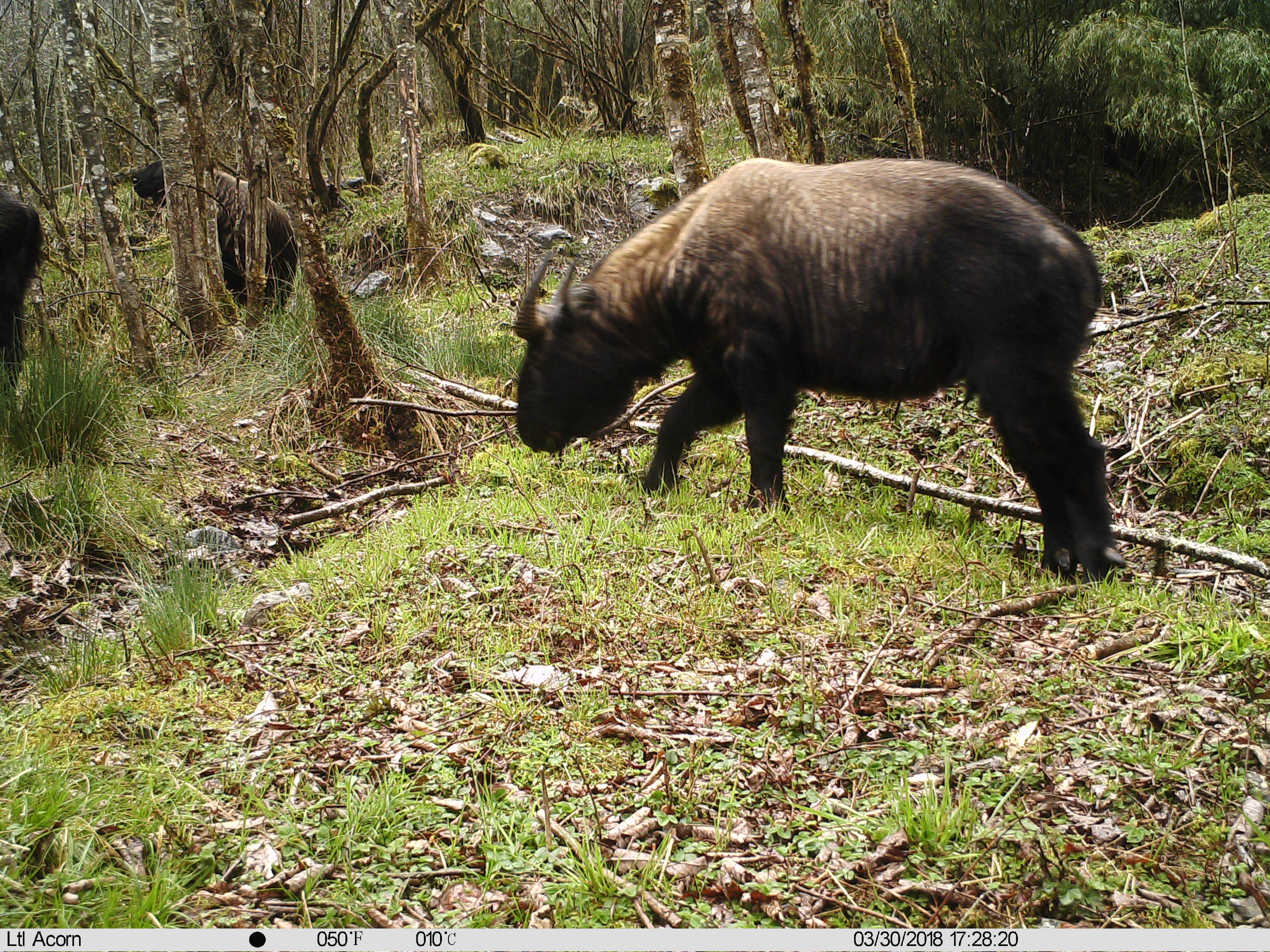

Supplement: Supplementary file 1 [file animals-14-02426-s001.zip › Budorcas taxicolor taxicolor-Part of the photos/IMAG1930.JPG]

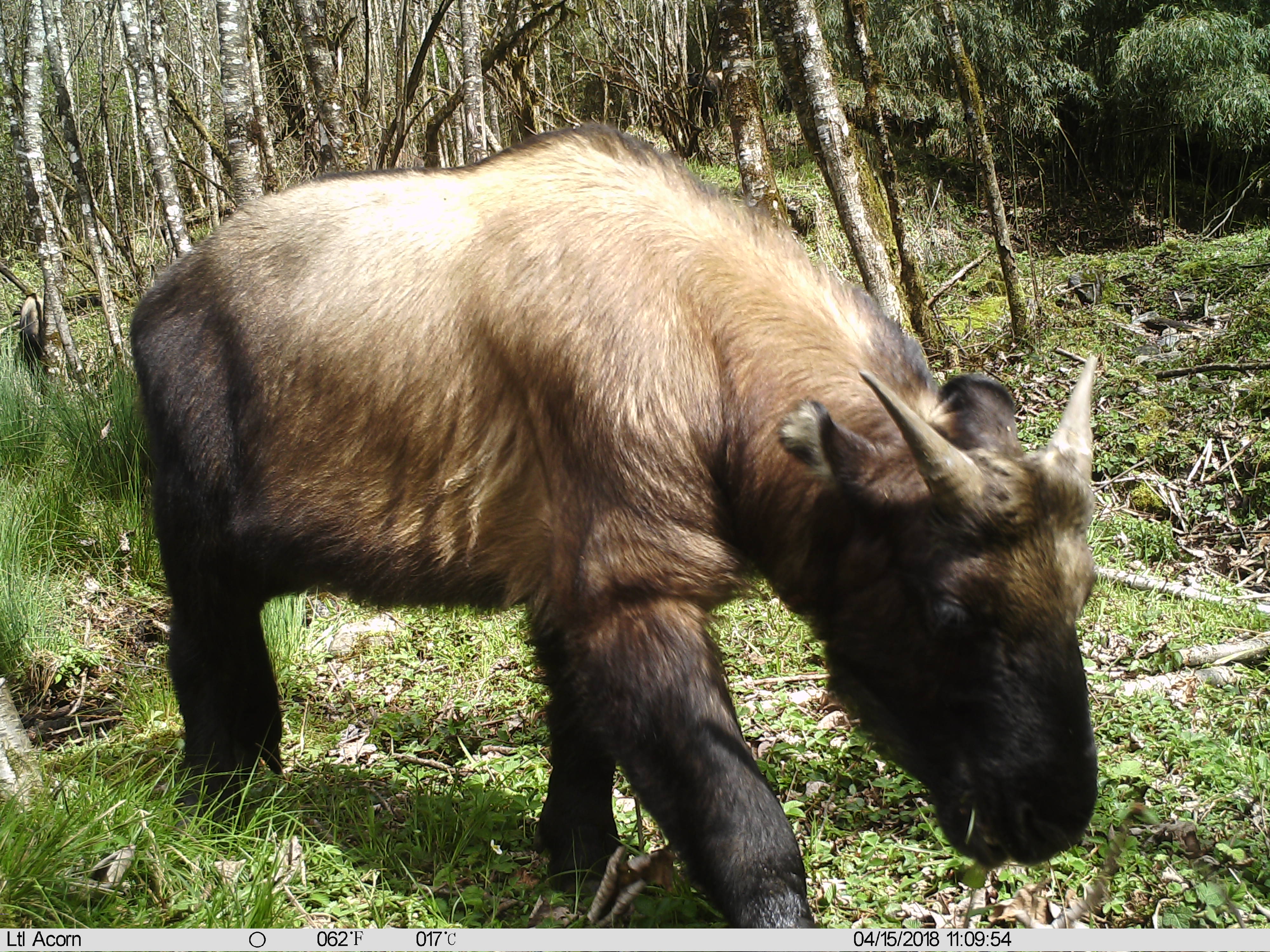

Supplement: Supplementary file 1 [file animals-14-02426-s001.zip › Budorcas taxicolor taxicolor-Part of the photos/IMAG2233.JPG]

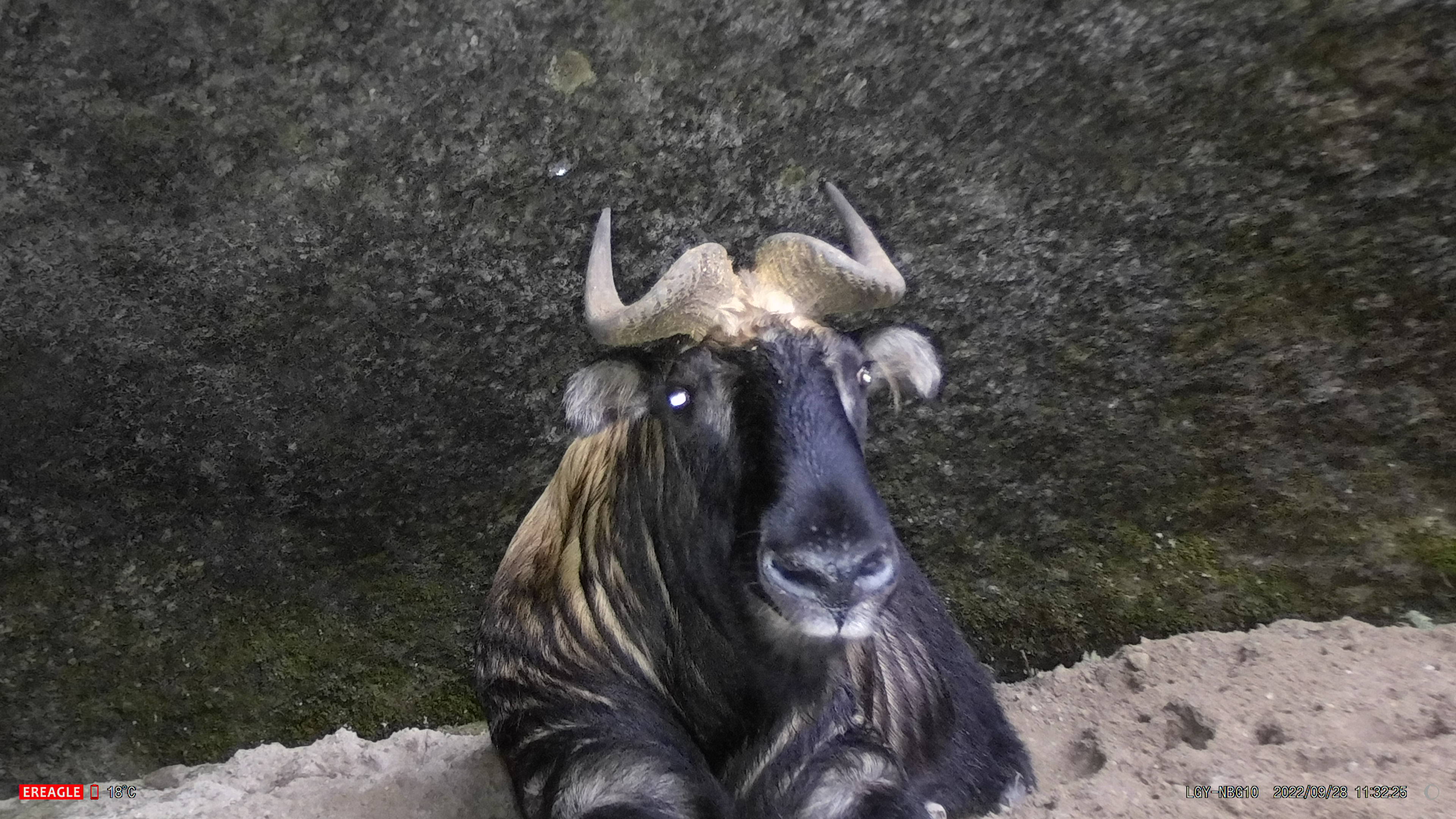

Supplement: Supplementary file 1 [file animals-14-02426-s001.zip › Budorcas taxicolor whitei-Part of the photos/ECSP2469.JPG]

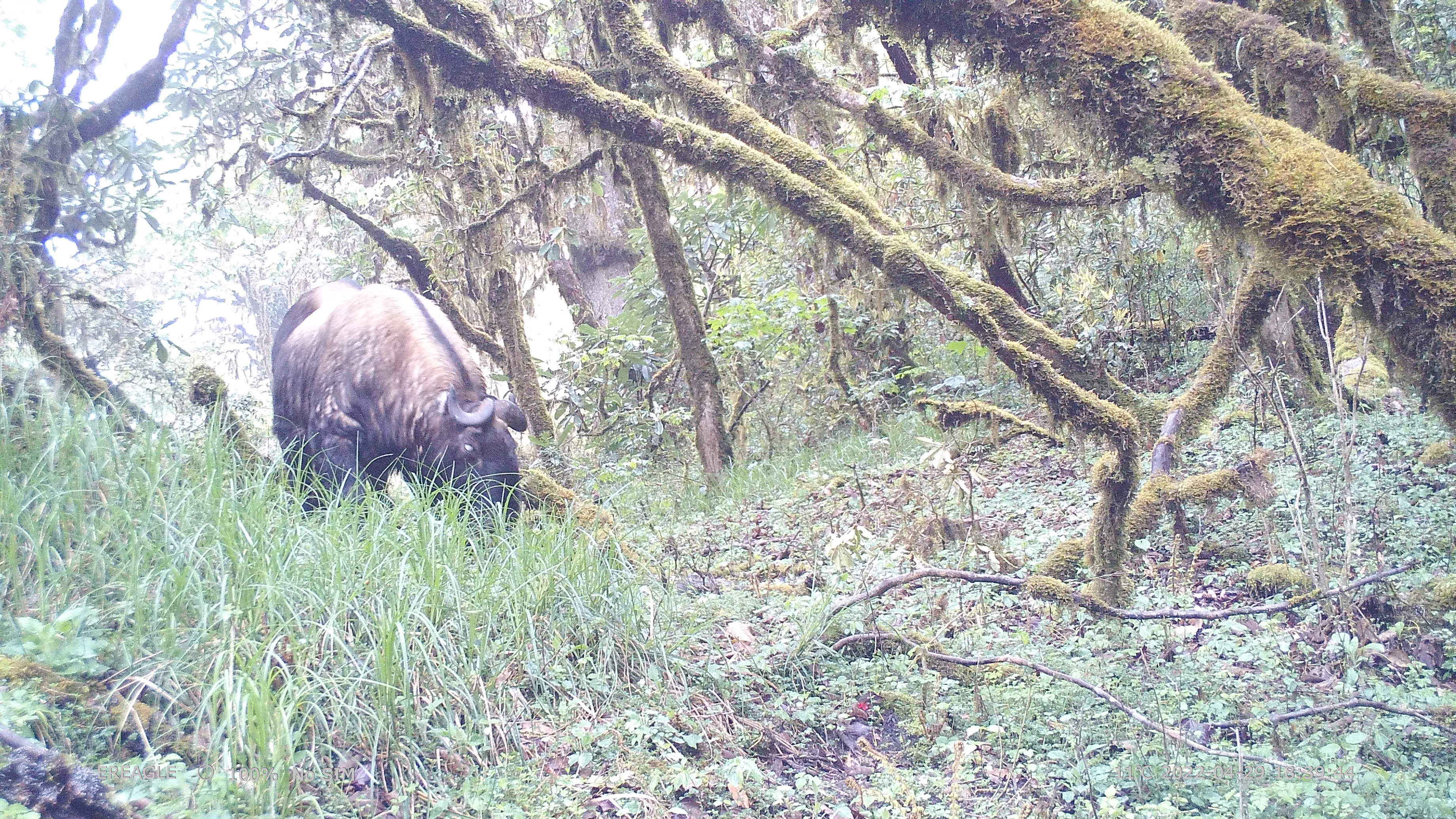

Supplement: Supplementary file 1 [file animals-14-02426-s001.zip › Budorcas taxicolor whitei-Part of the photos/Ere 0084.JPG]

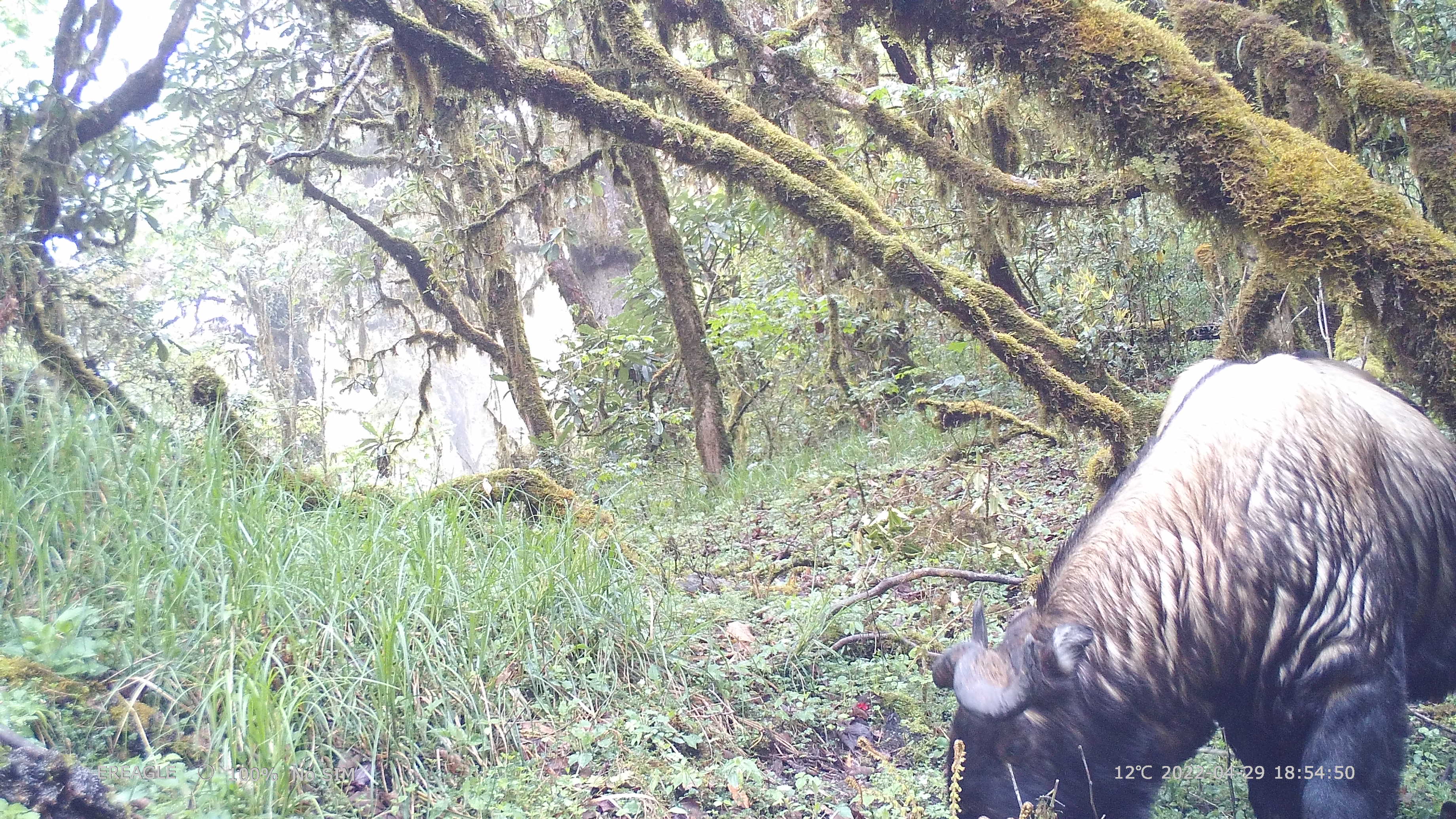

Supplement: Supplementary file 1 [file animals-14-02426-s001.zip › Budorcas taxicolor whitei-Part of the photos/Ere 0102 (2).JPG]

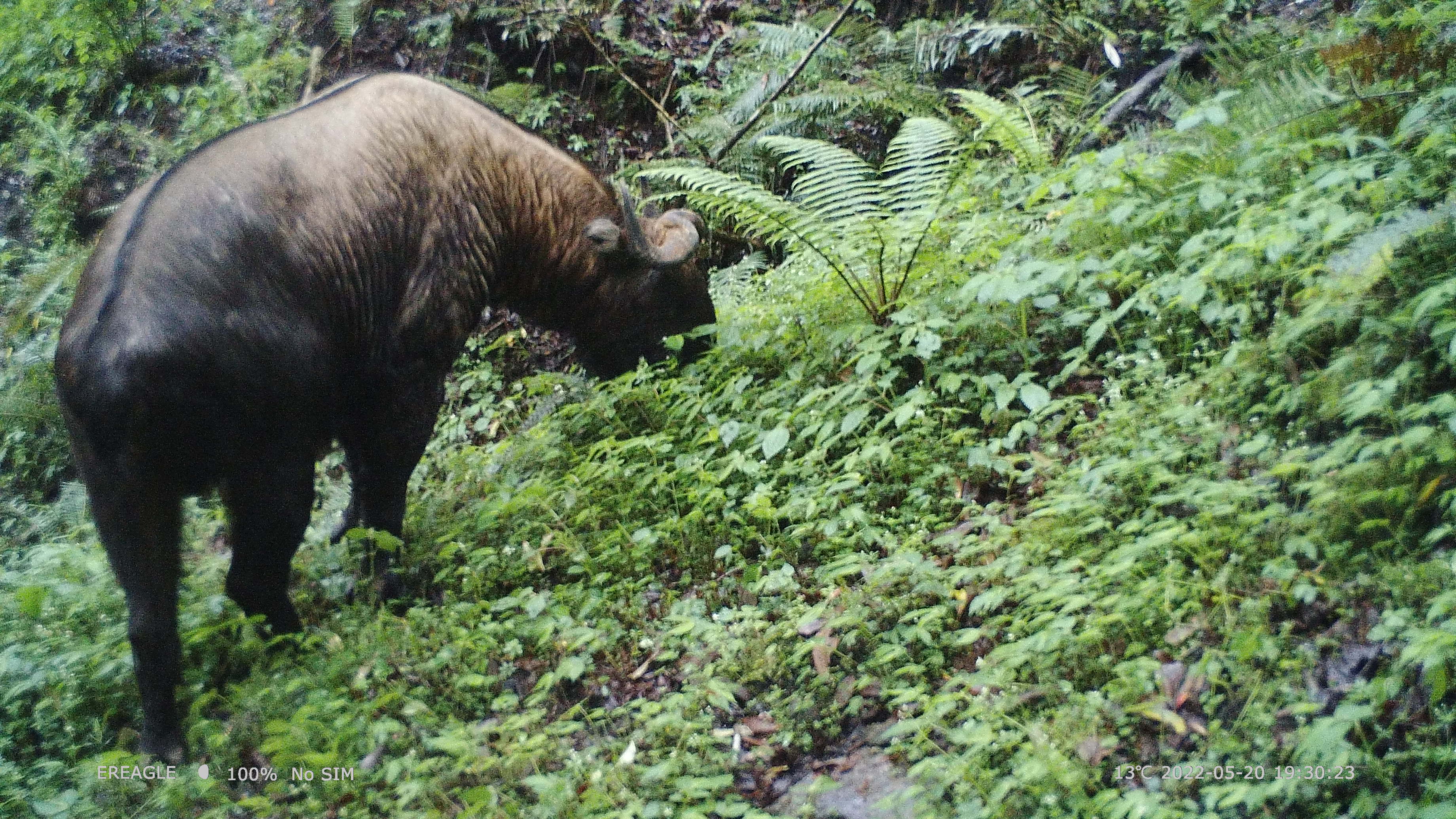

Supplement: Supplementary file 1 [file animals-14-02426-s001.zip › Budorcas taxicolor whitei-Part of the photos/Ere 0120.JPG]

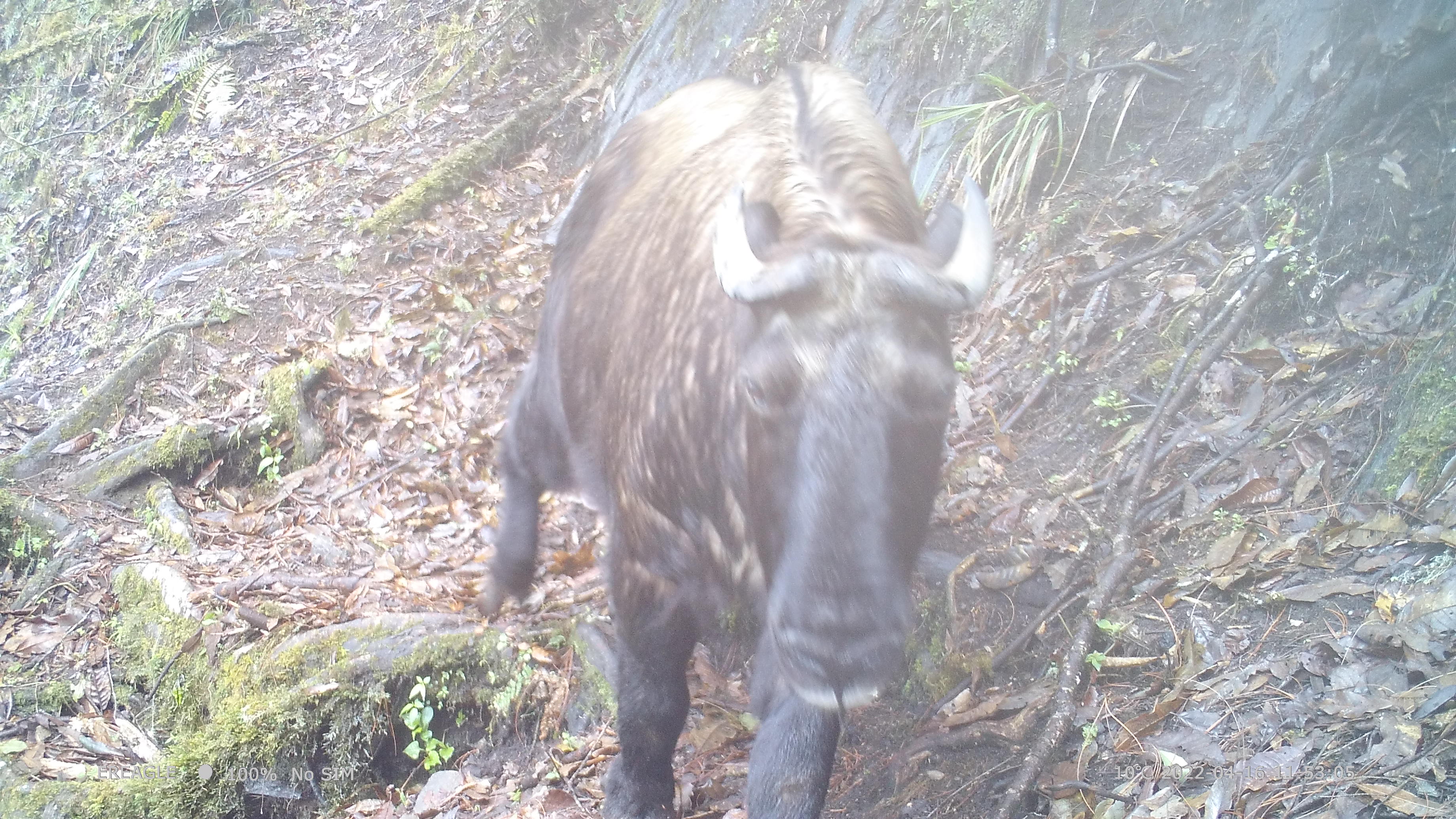

Supplement: Supplementary file 1 [file animals-14-02426-s001.zip › Budorcas taxicolor whitei-Part of the photos/Ere 0125 (4).JPG]

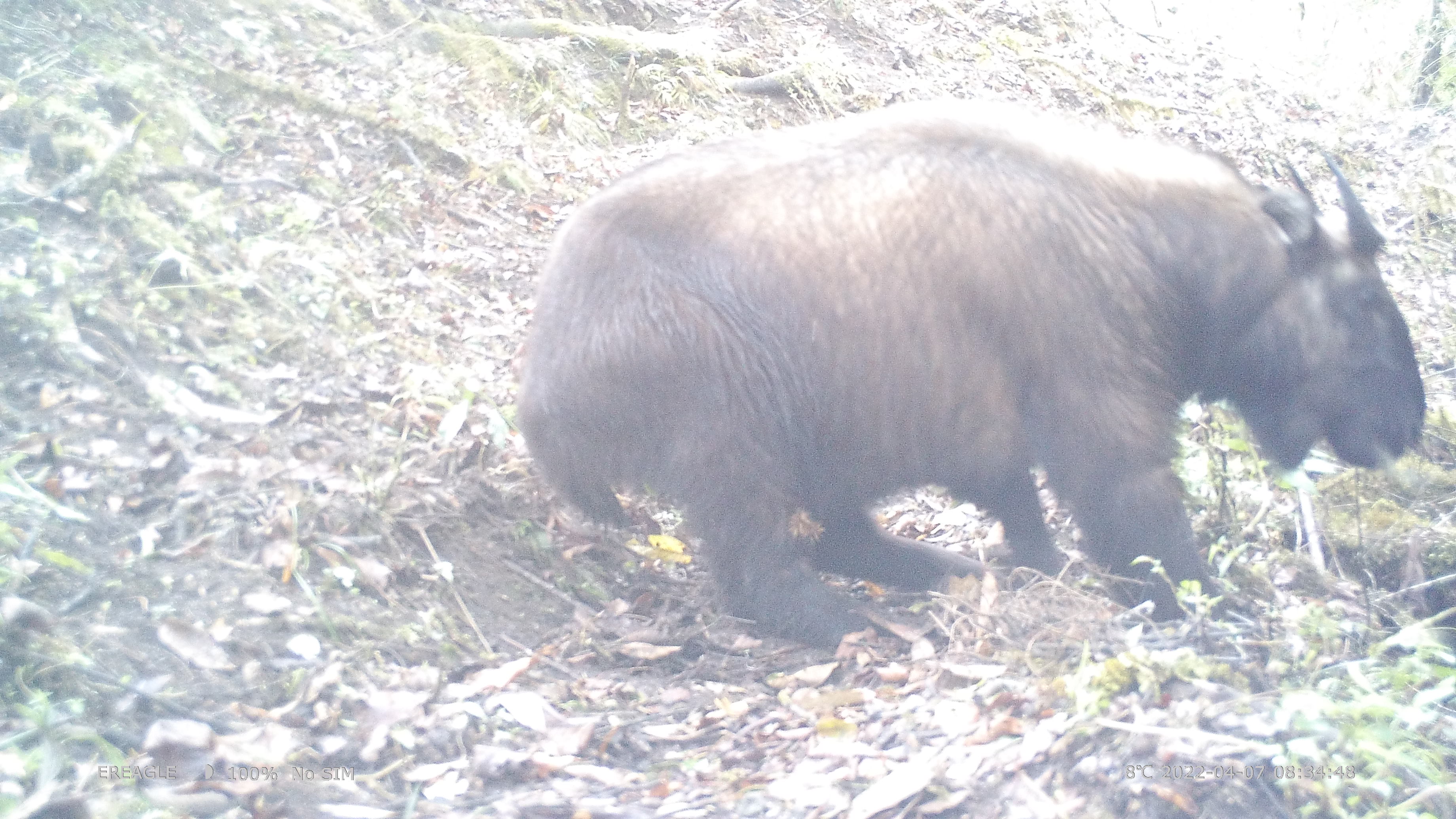

Supplement: Supplementary file 1 [file animals-14-02426-s001.zip › Budorcas taxicolor whitei-Part of the photos/Ere 0134.JPG]

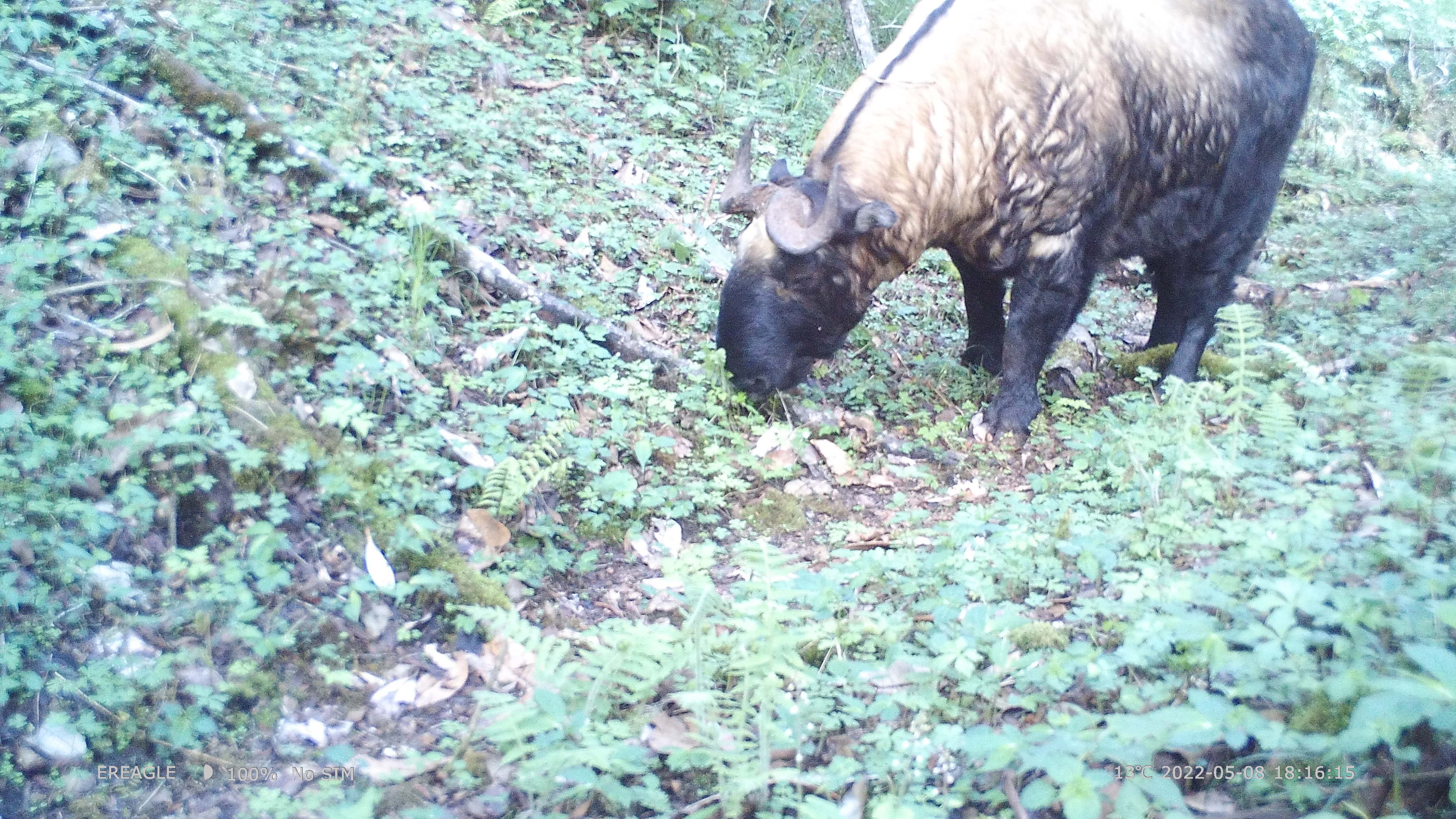

Supplement: Supplementary file 1 [file animals-14-02426-s001.zip › Budorcas taxicolor whitei-Part of the photos/Ere 0170 (6).JPG]

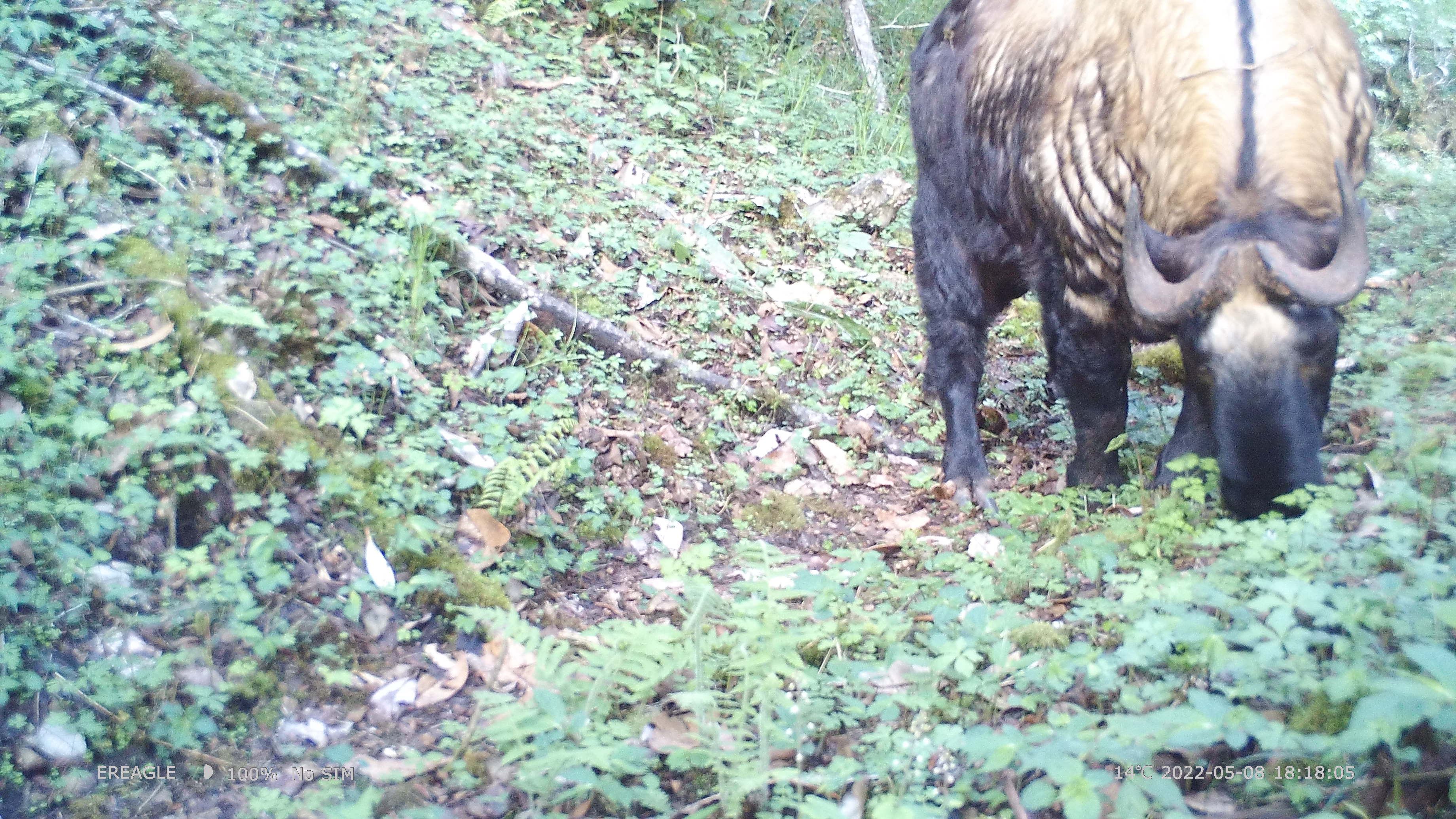

Supplement: Supplementary file 1 [file animals-14-02426-s001.zip › Budorcas taxicolor whitei-Part of the photos/Ere 0173 (7).JPG]

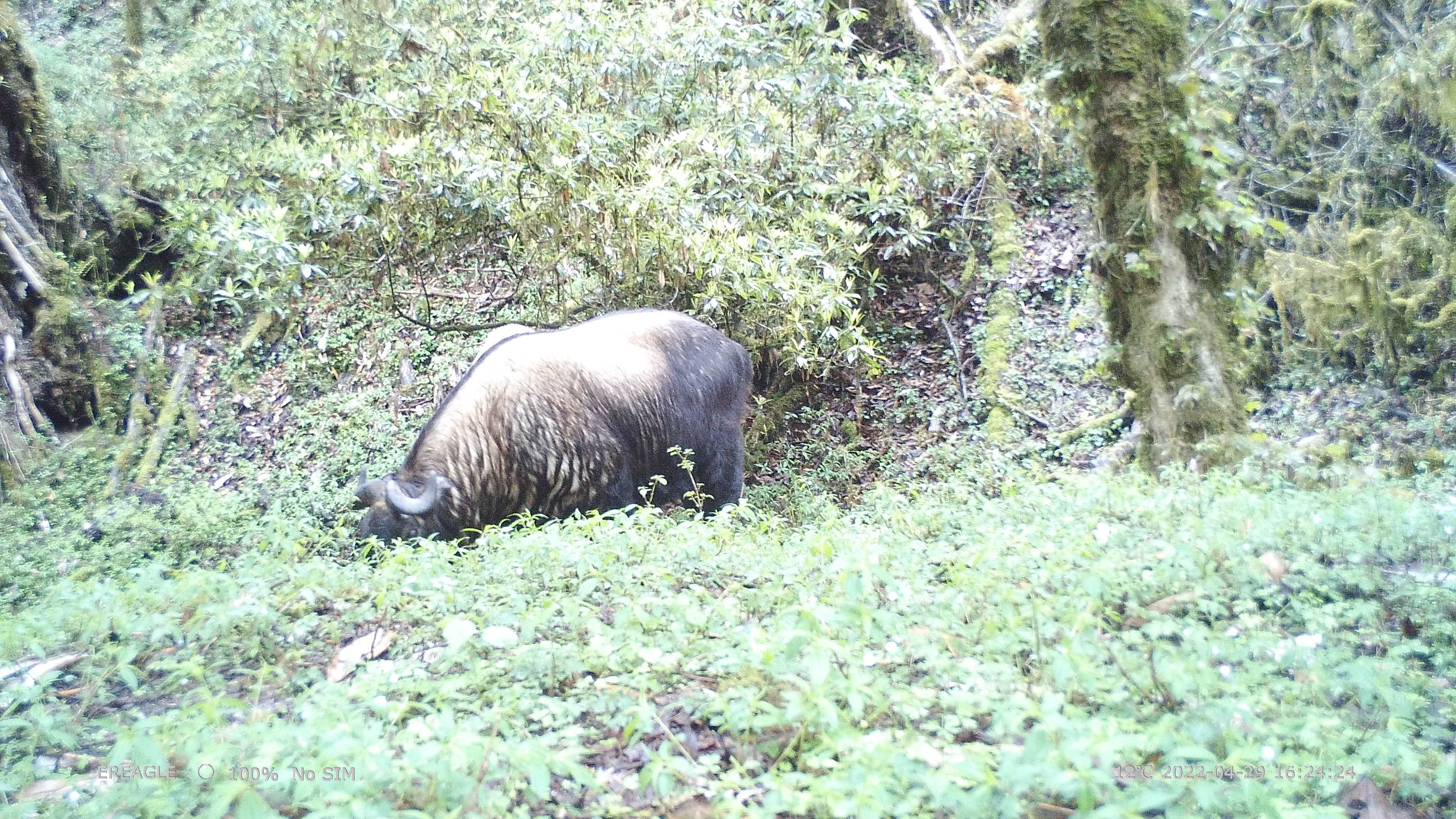

Supplement: Supplementary file 1 [file animals-14-02426-s001.zip › Budorcas taxicolor whitei-Part of the photos/Ere 0184 (2).JPG]

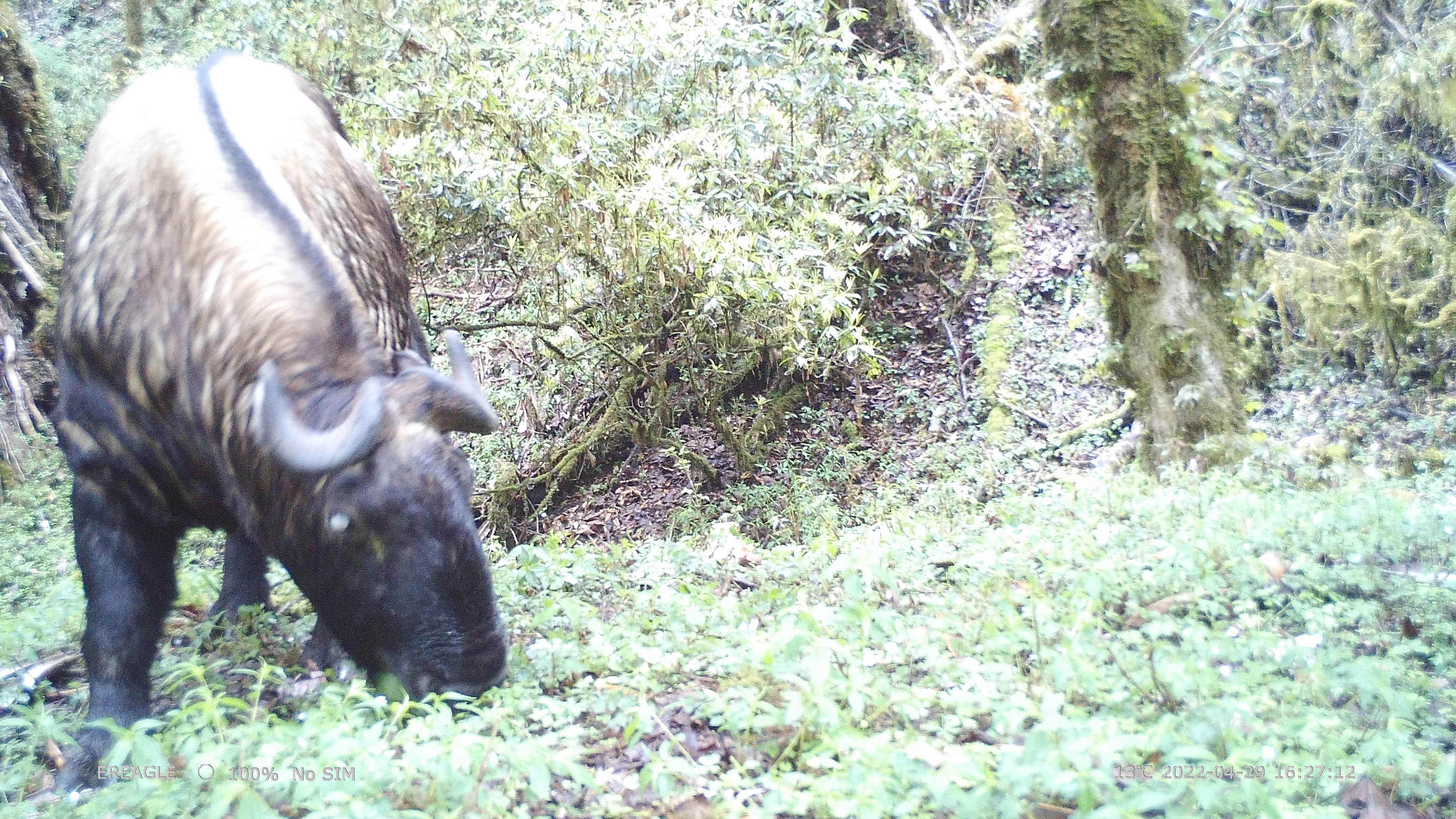

Supplement: Supplementary file 1 [file animals-14-02426-s001.zip › Budorcas taxicolor whitei-Part of the photos/Ere 0192 (2).JPG]

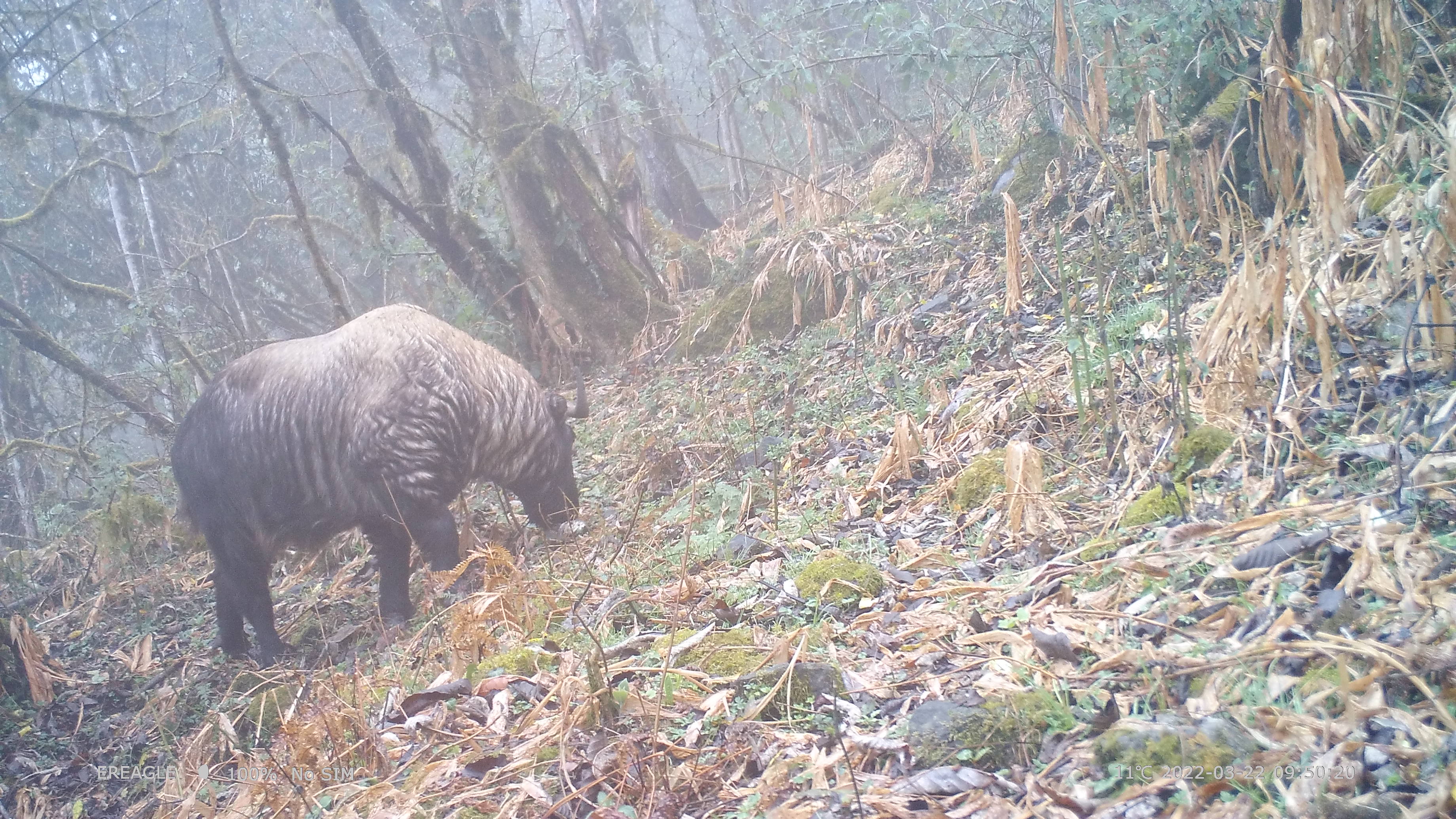

Supplement: Supplementary file 1 [file animals-14-02426-s001.zip › Budorcas taxicolor whitei-Part of the photos/Ere 0193 (2).JPG]

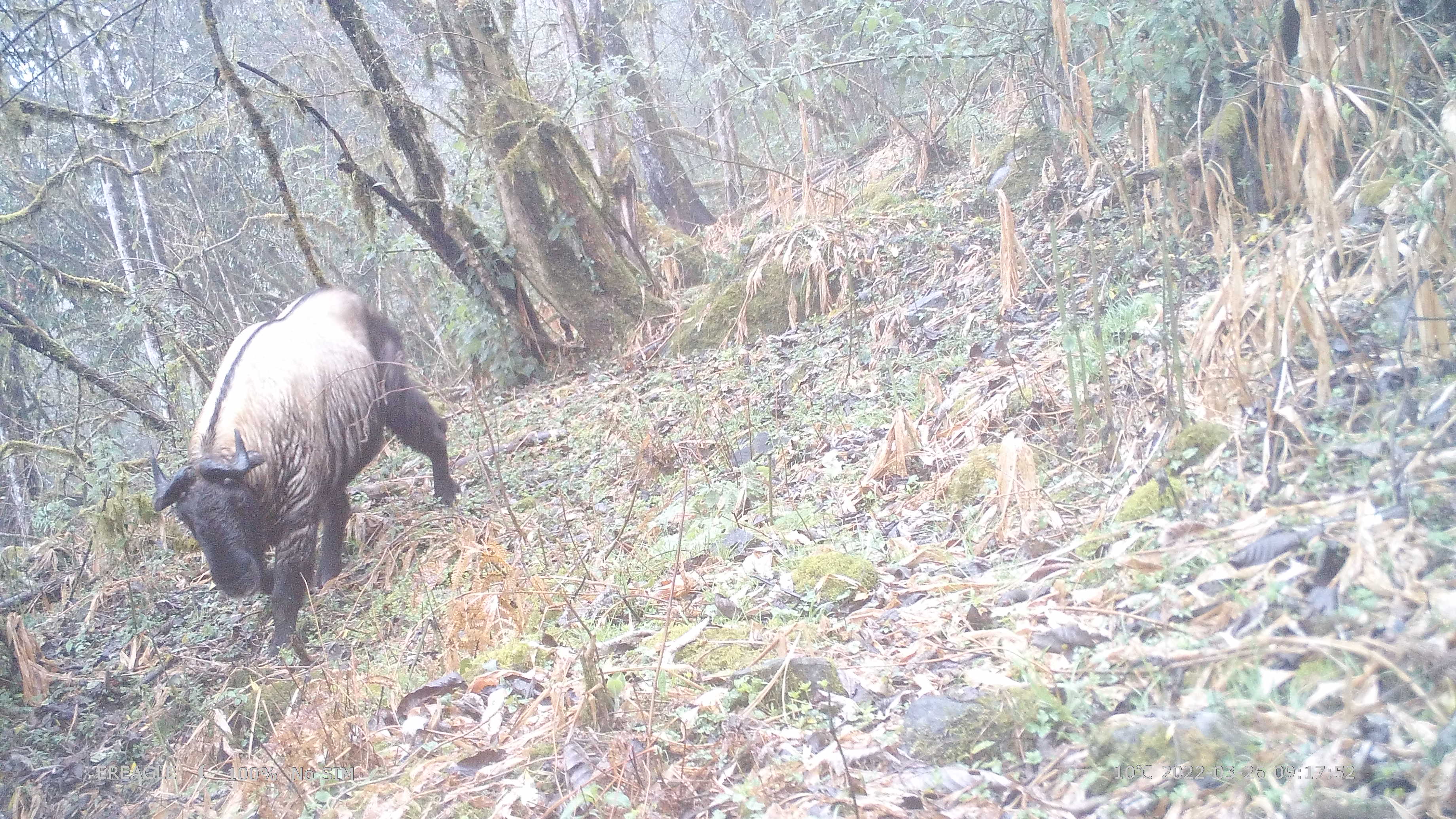

Supplement: Supplementary file 1 [file animals-14-02426-s001.zip › Budorcas taxicolor whitei-Part of the photos/Ere 0207 (2).JPG]

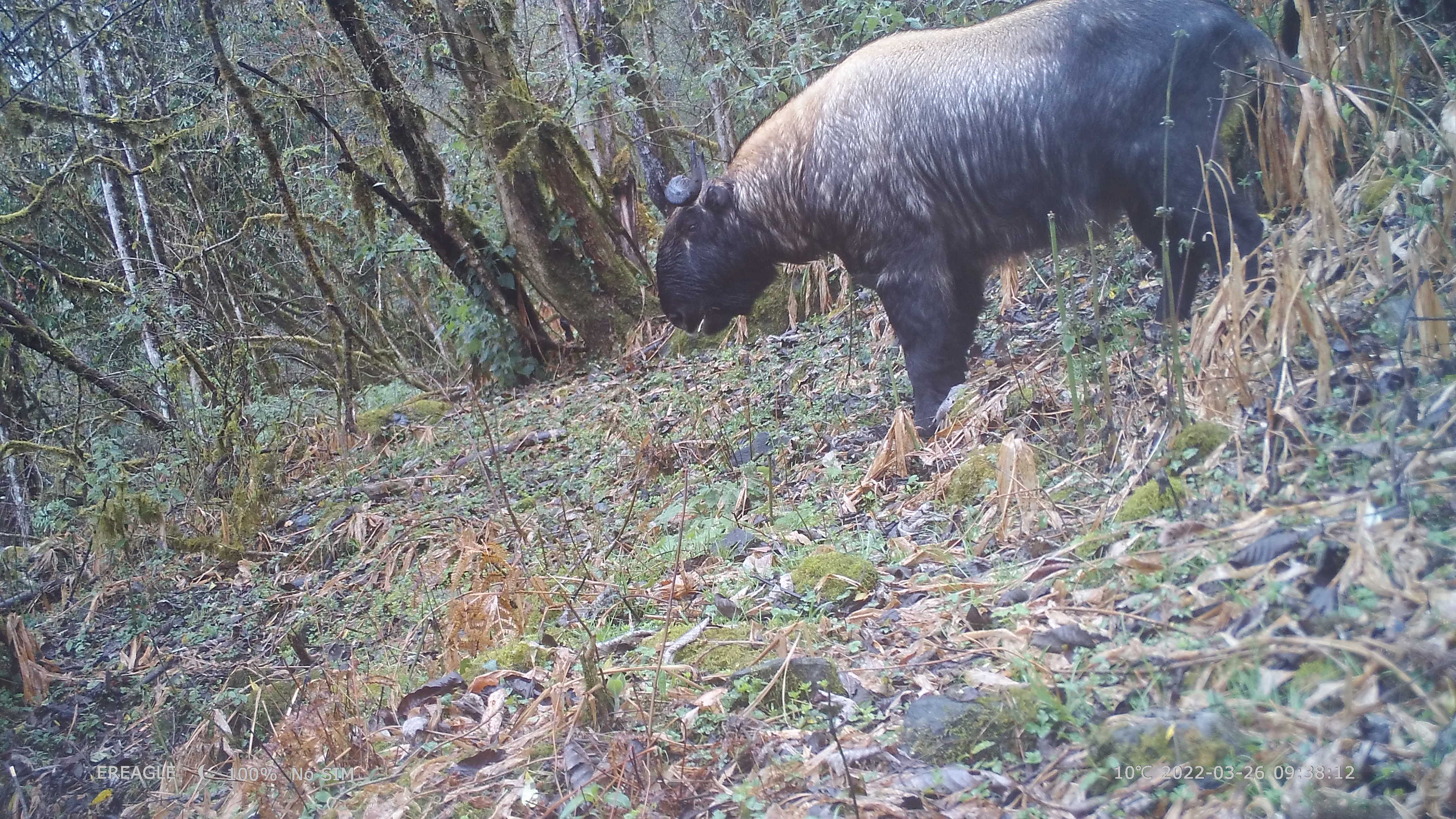

Supplement: Supplementary file 1 [file animals-14-02426-s001.zip › Budorcas taxicolor whitei-Part of the photos/Ere 0210 (2).JPG]

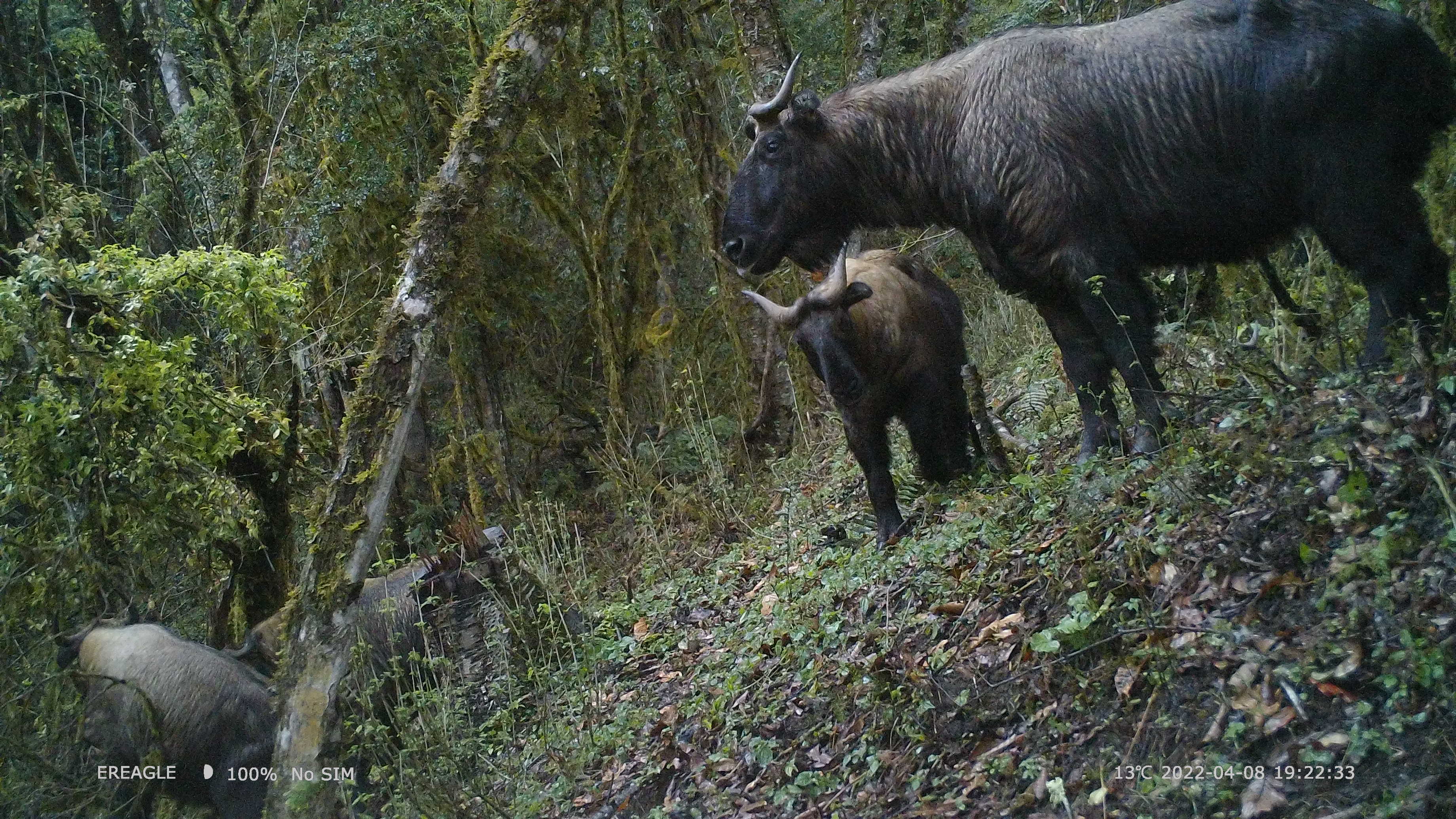

Supplement: Supplementary file 1 [file animals-14-02426-s001.zip › Budorcas taxicolor whitei-Part of the photos/Ere 0223 (3).JPG]

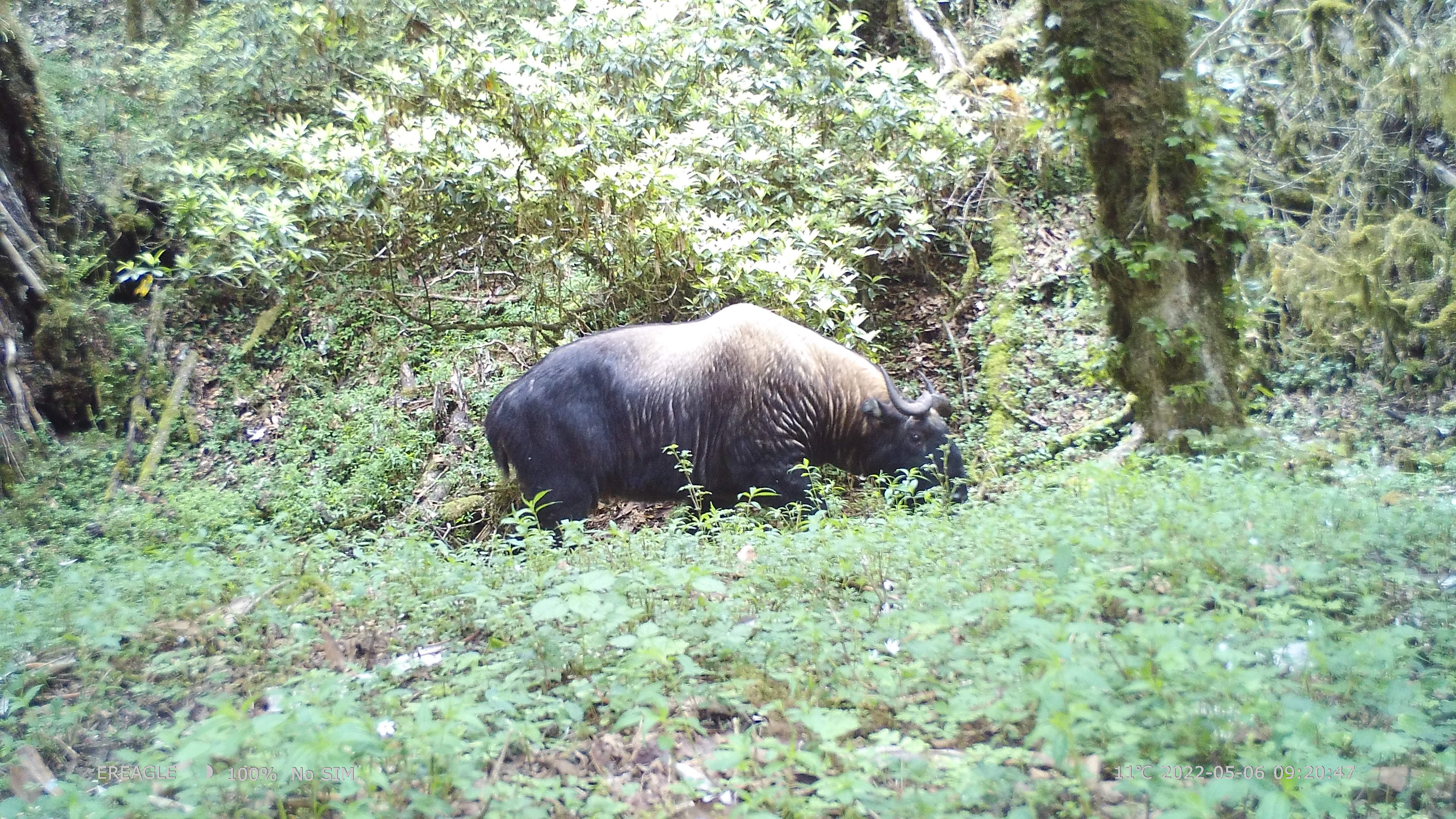

Supplement: Supplementary file 1 [file animals-14-02426-s001.zip › Budorcas taxicolor whitei-Part of the photos/Ere 0244 (2).JPG]

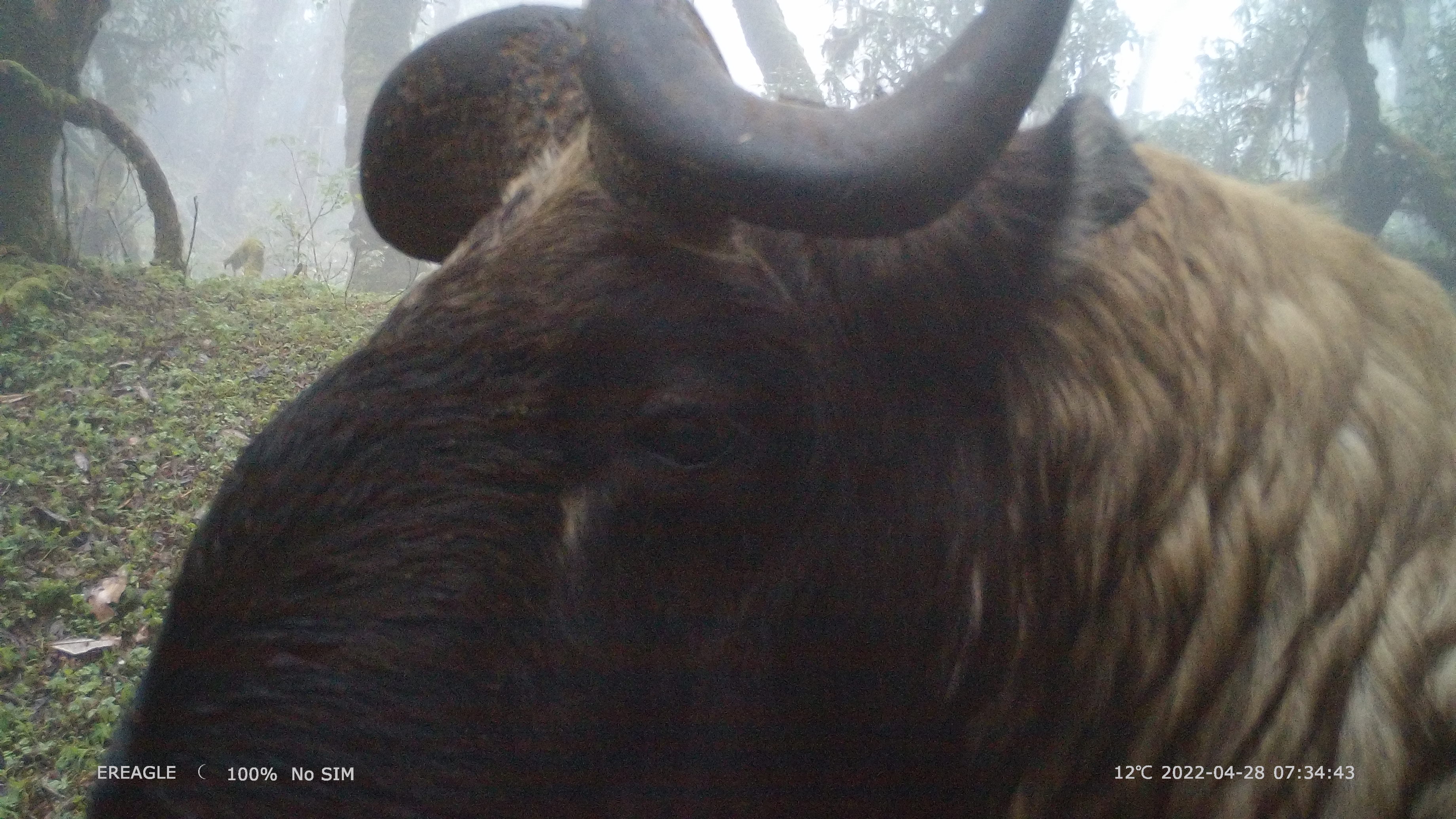

Supplement: Supplementary file 1 [file animals-14-02426-s001.zip › Budorcas taxicolor whitei-Part of the photos/Ere 0329 (2).JPG]

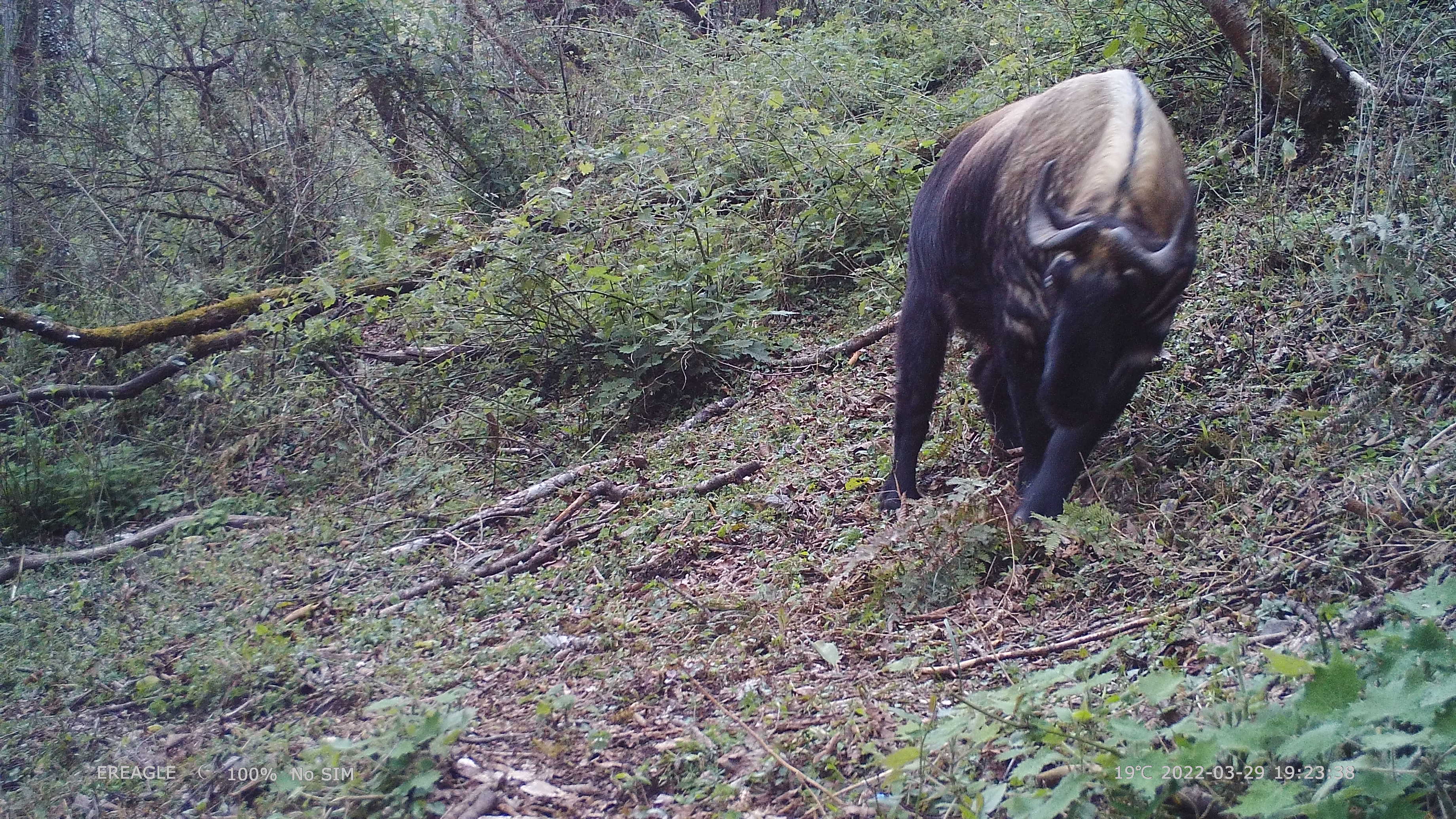

Supplement: Supplementary file 1 [file animals-14-02426-s001.zip › Budorcas taxicolor whitei-Part of the photos/Ere 0370 (3).JPG]

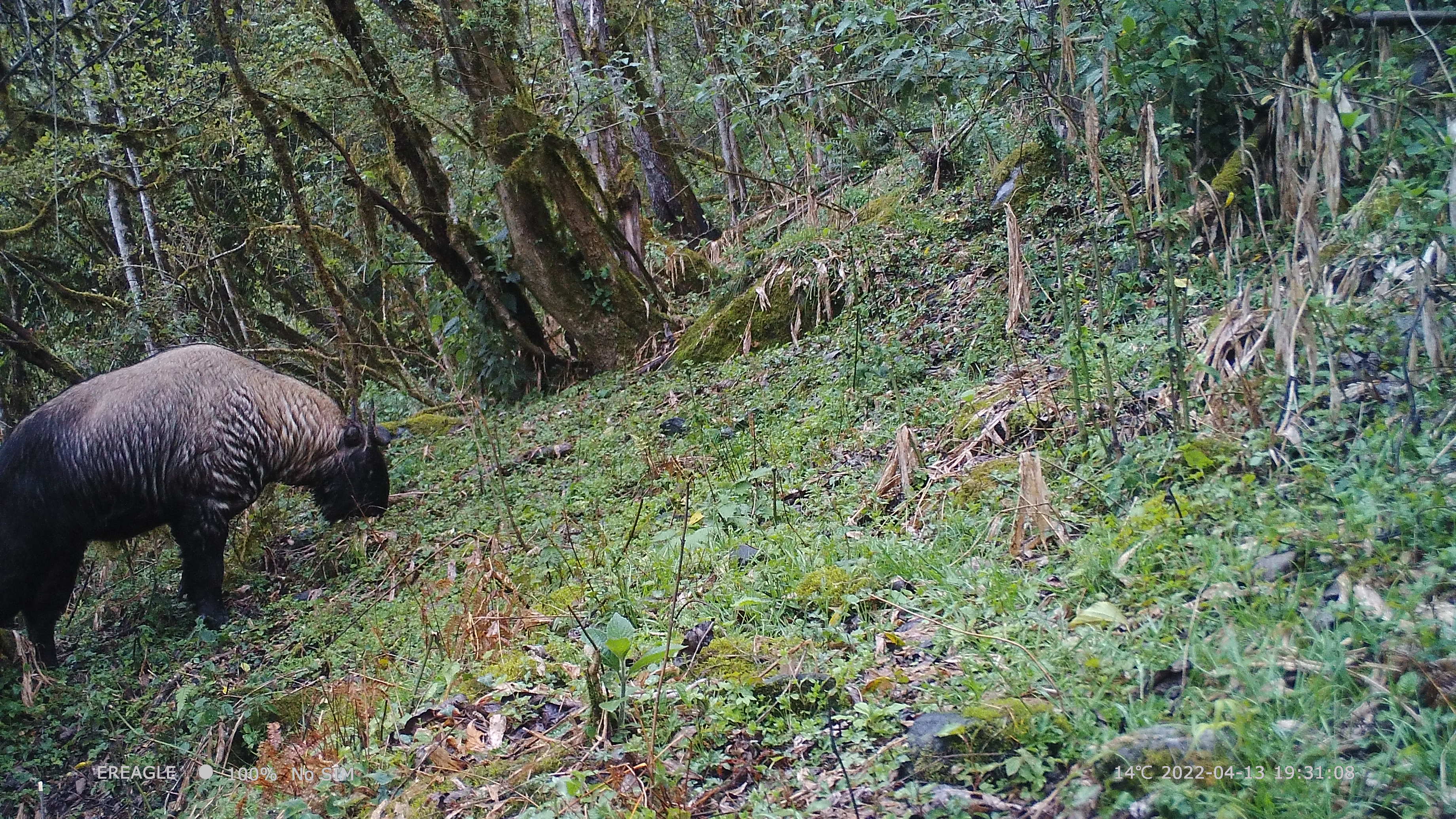

Supplement: Supplementary file 1 [file animals-14-02426-s001.zip › Budorcas taxicolor whitei-Part of the photos/Ere 0417.JPG]

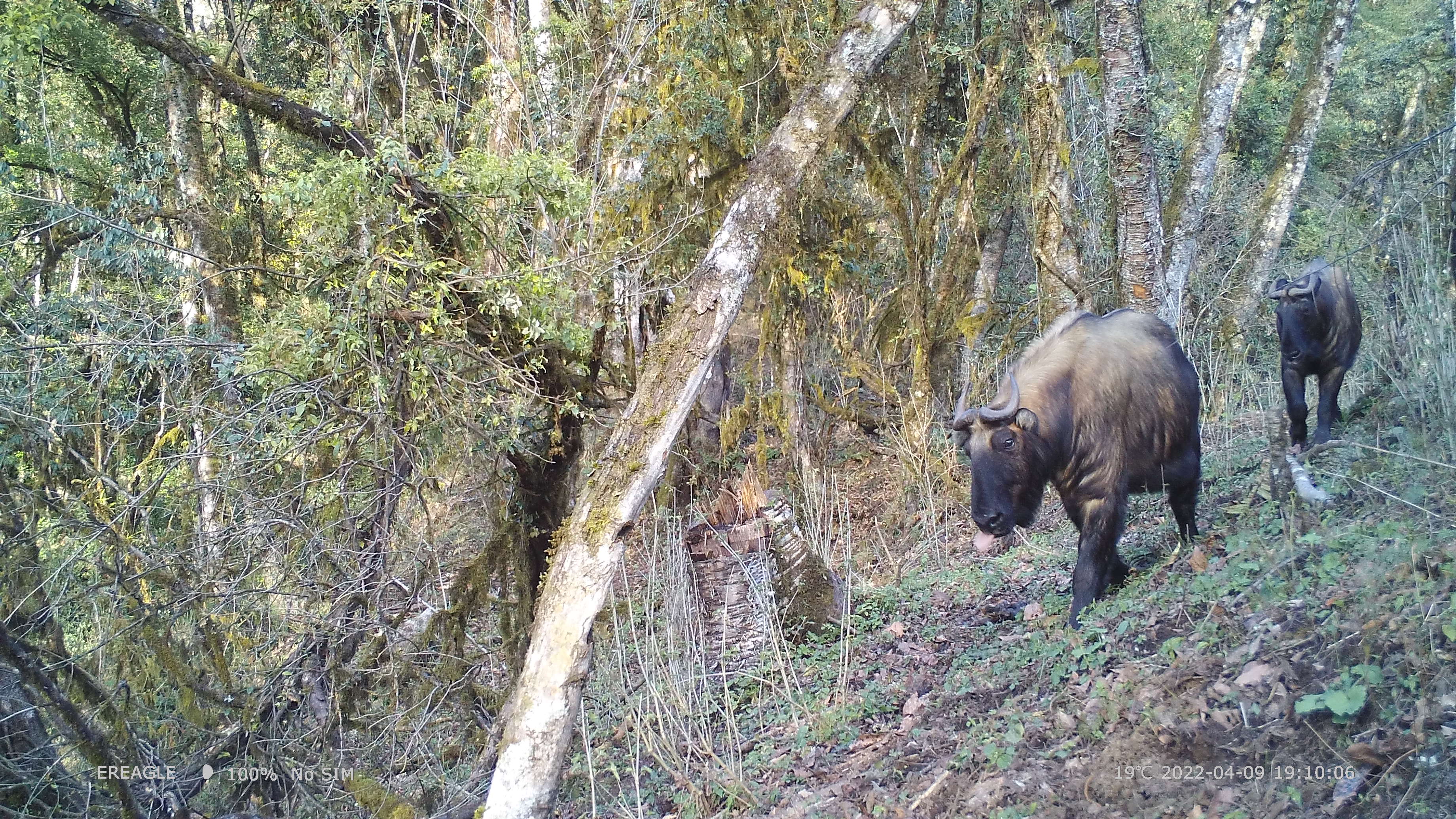

Supplement: Supplementary file 1 [file animals-14-02426-s001.zip › Budorcas taxicolor whitei-Part of the photos/Ere 0429 (2).JPG]

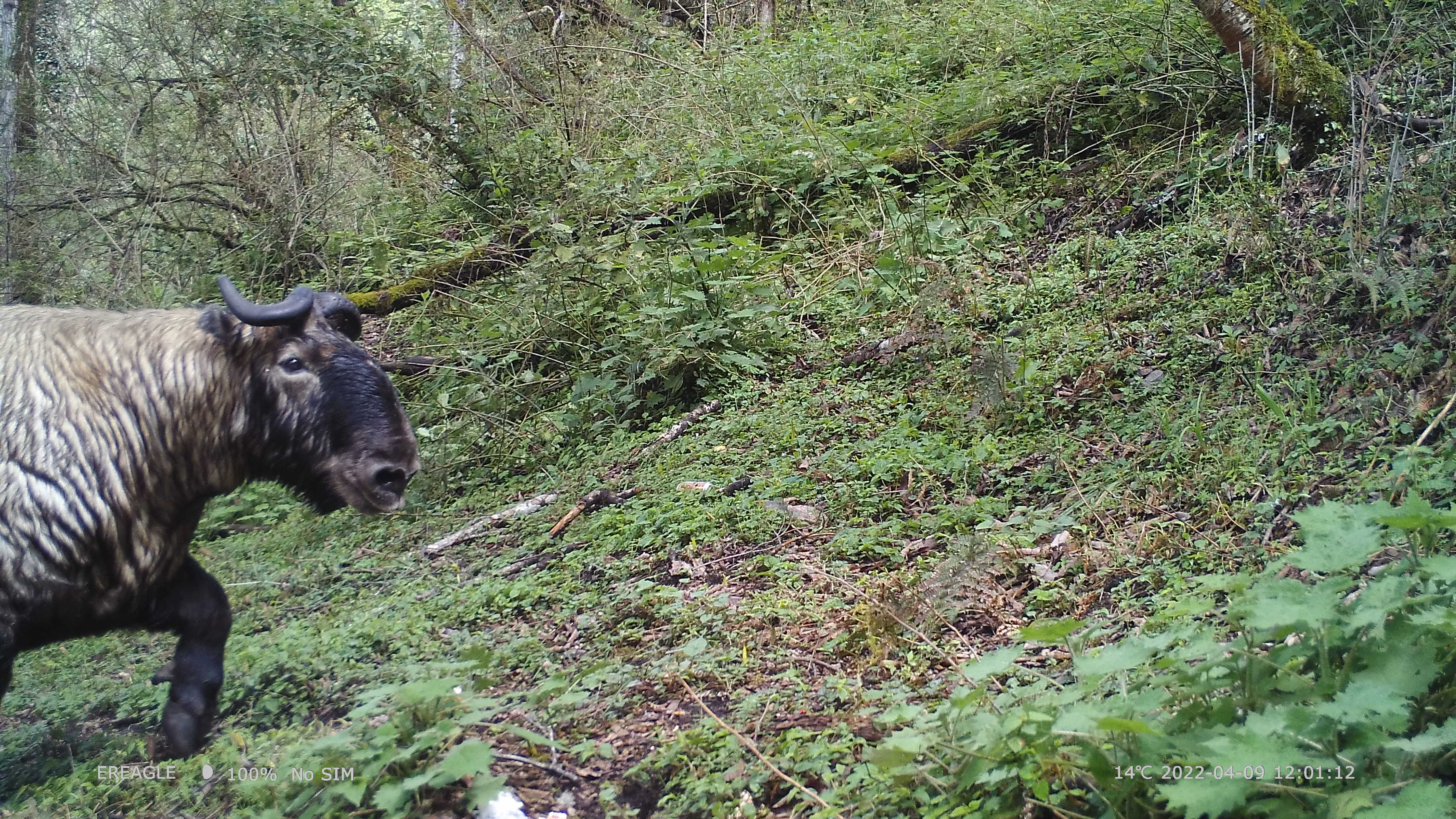

Supplement: Supplementary file 1 [file animals-14-02426-s001.zip › Budorcas taxicolor whitei-Part of the photos/Ere 0490.JPG]

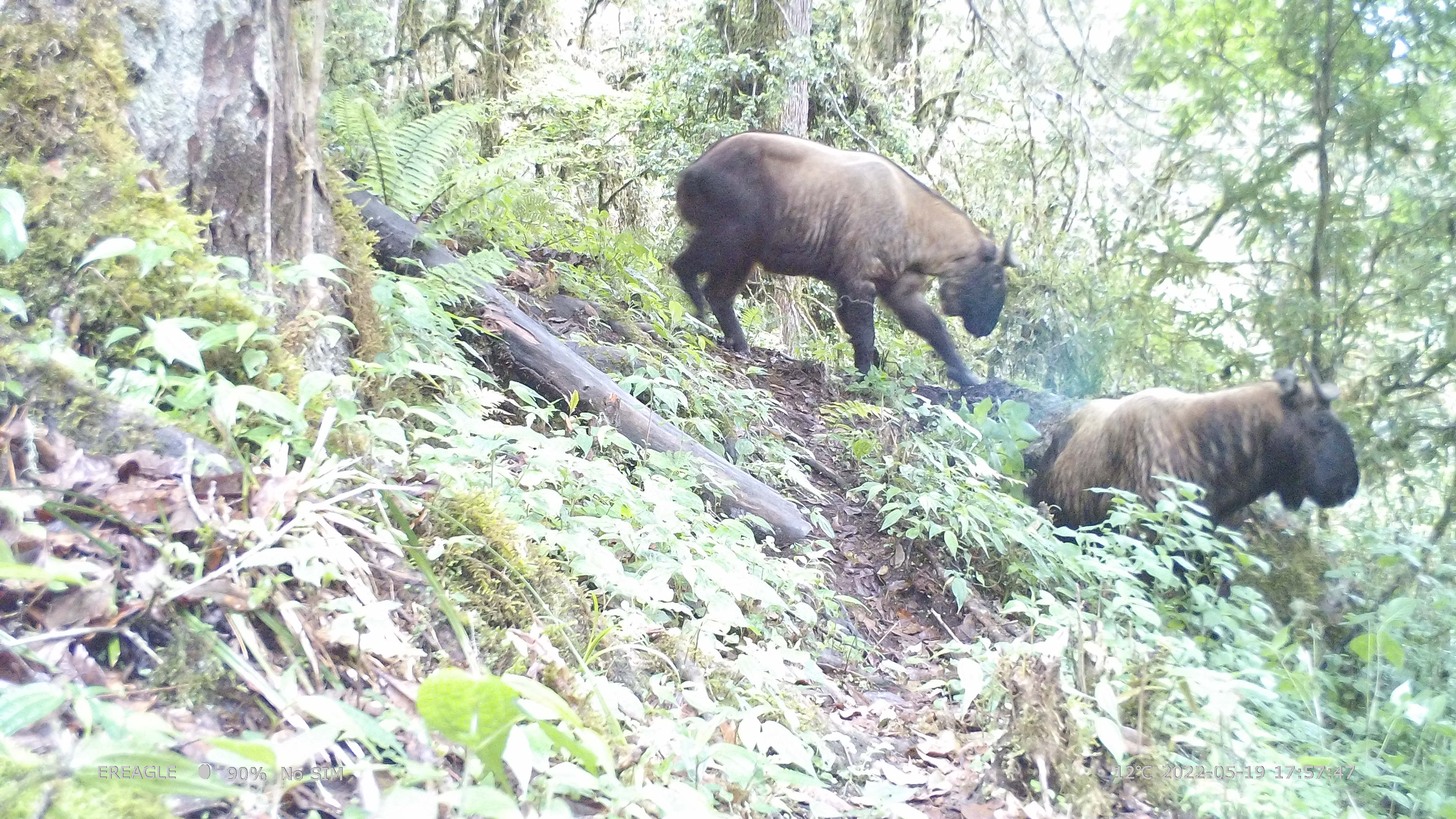

Supplement: Supplementary file 1 [file animals-14-02426-s001.zip › Budorcas taxicolor whitei-Part of the photos/Ere 0509.JPG]

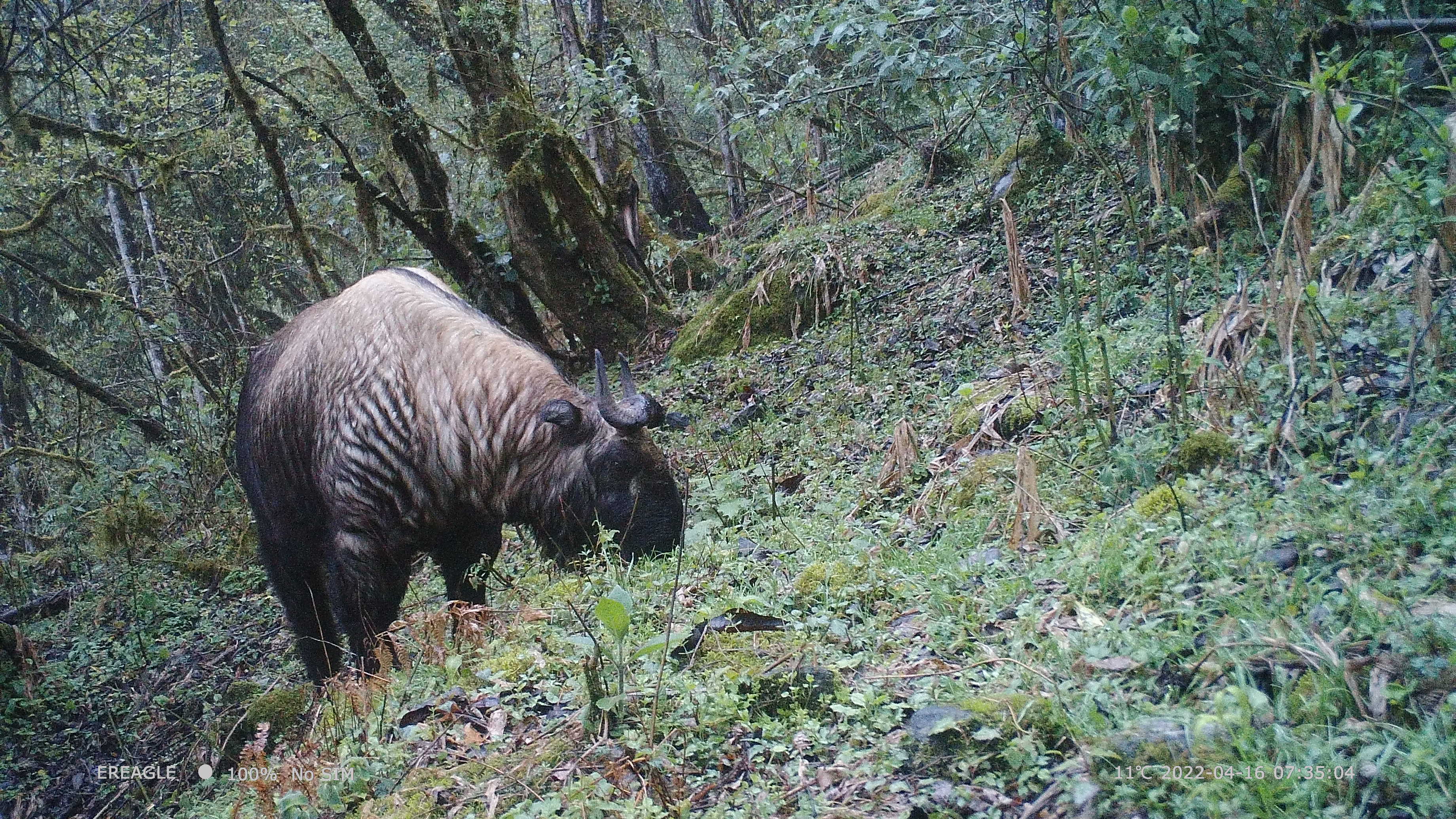

Supplement: Supplementary file 1 [file animals-14-02426-s001.zip › Budorcas taxicolor whitei-Part of the photos/Ere 0535.JPG]

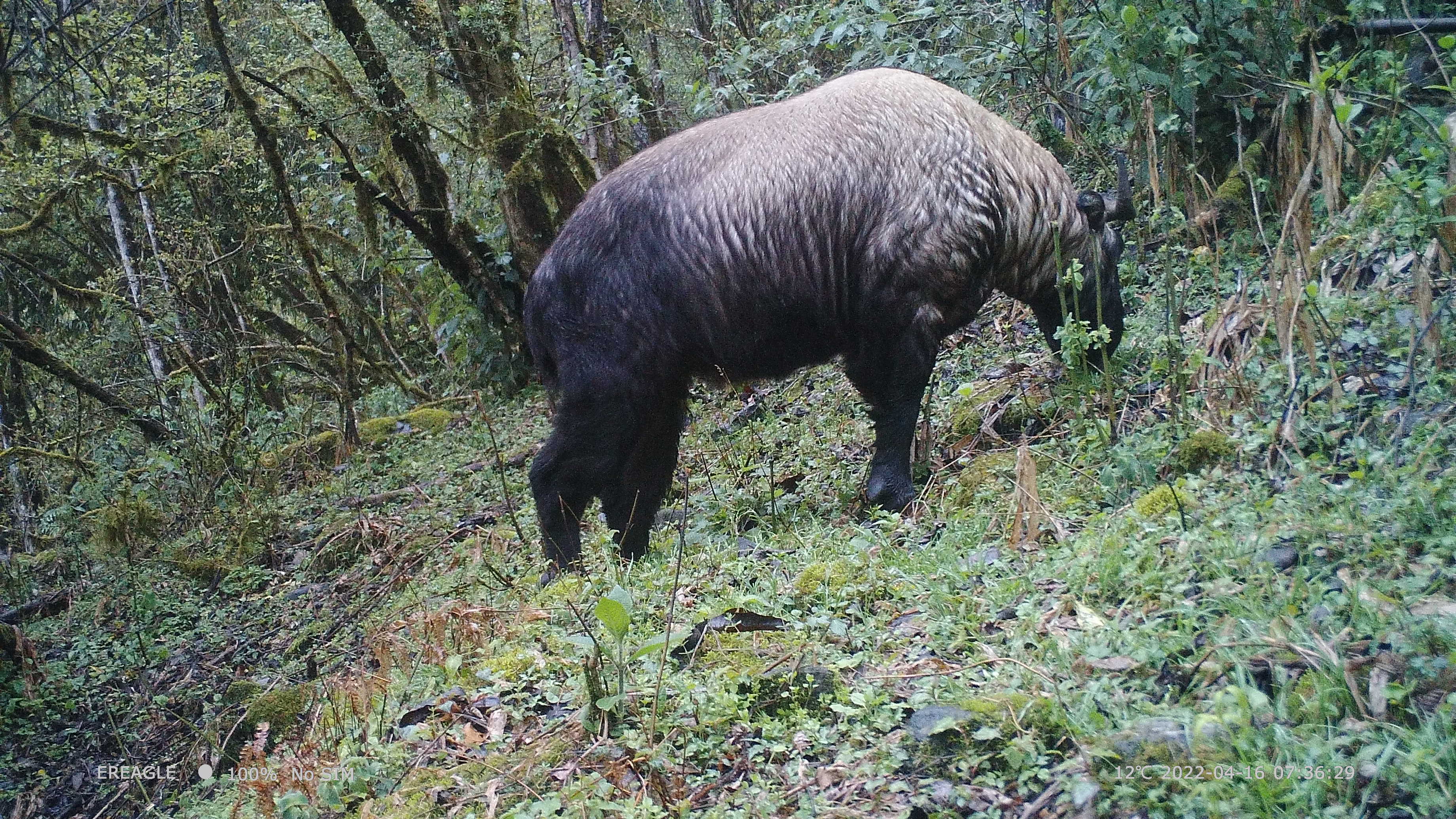

Supplement: Supplementary file 1 [file animals-14-02426-s001.zip › Budorcas taxicolor whitei-Part of the photos/Ere 0539.JPG]

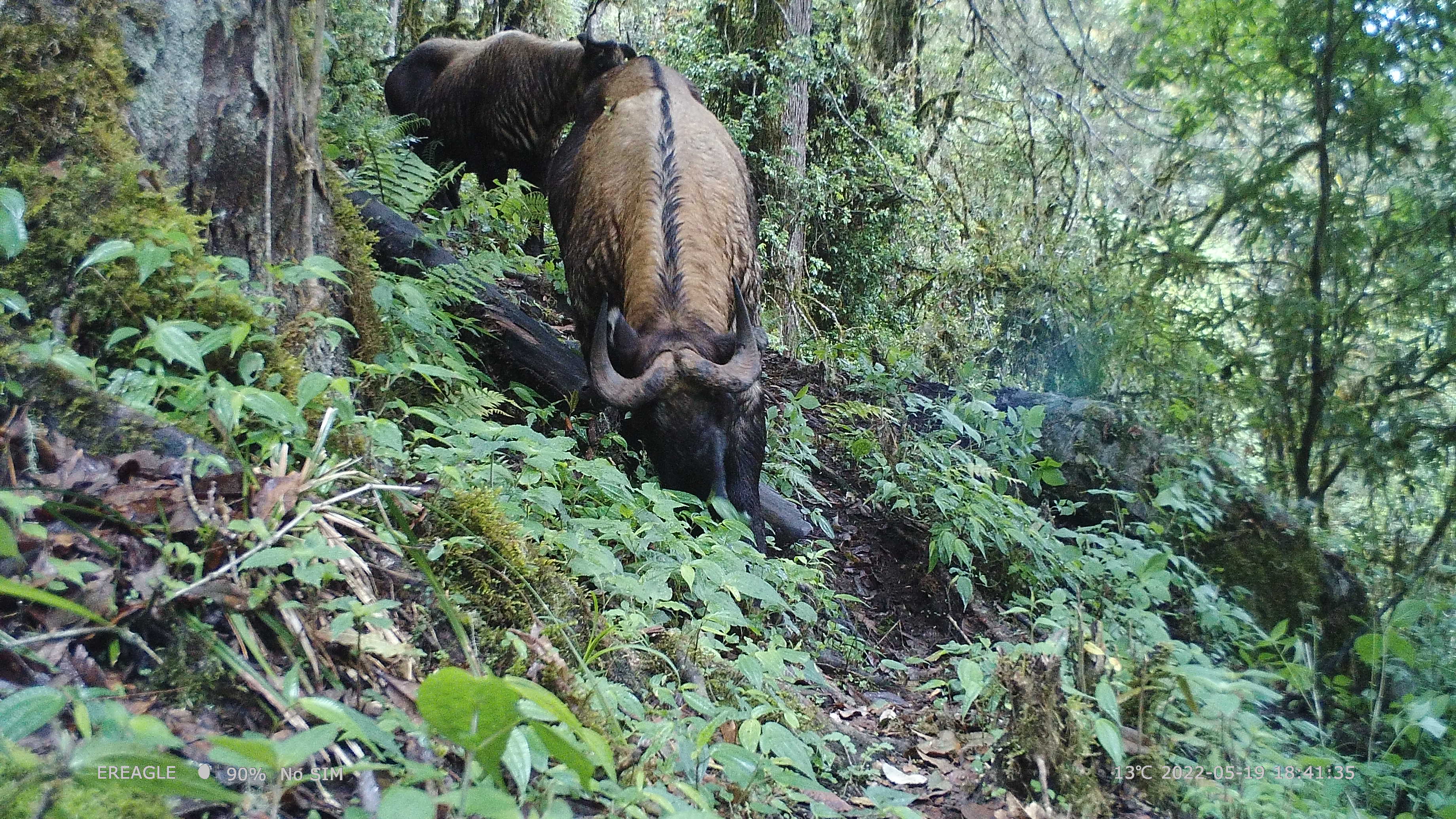

Supplement: Supplementary file 1 [file animals-14-02426-s001.zip › Budorcas taxicolor whitei-Part of the photos/Ere 0541.JPG]

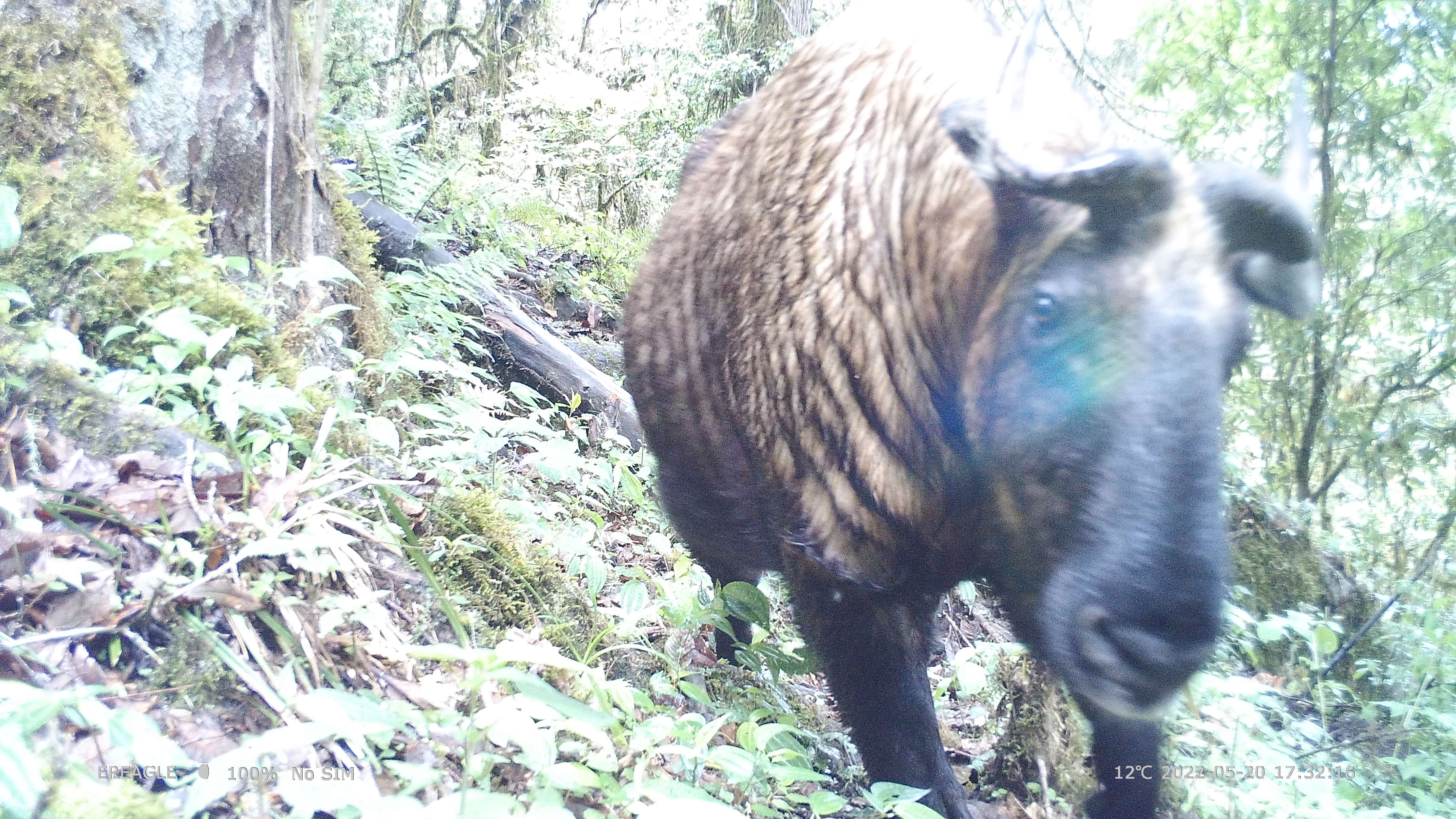

Supplement: Supplementary file 1 [file animals-14-02426-s001.zip › Budorcas taxicolor whitei-Part of the photos/Ere 0558.JPG]

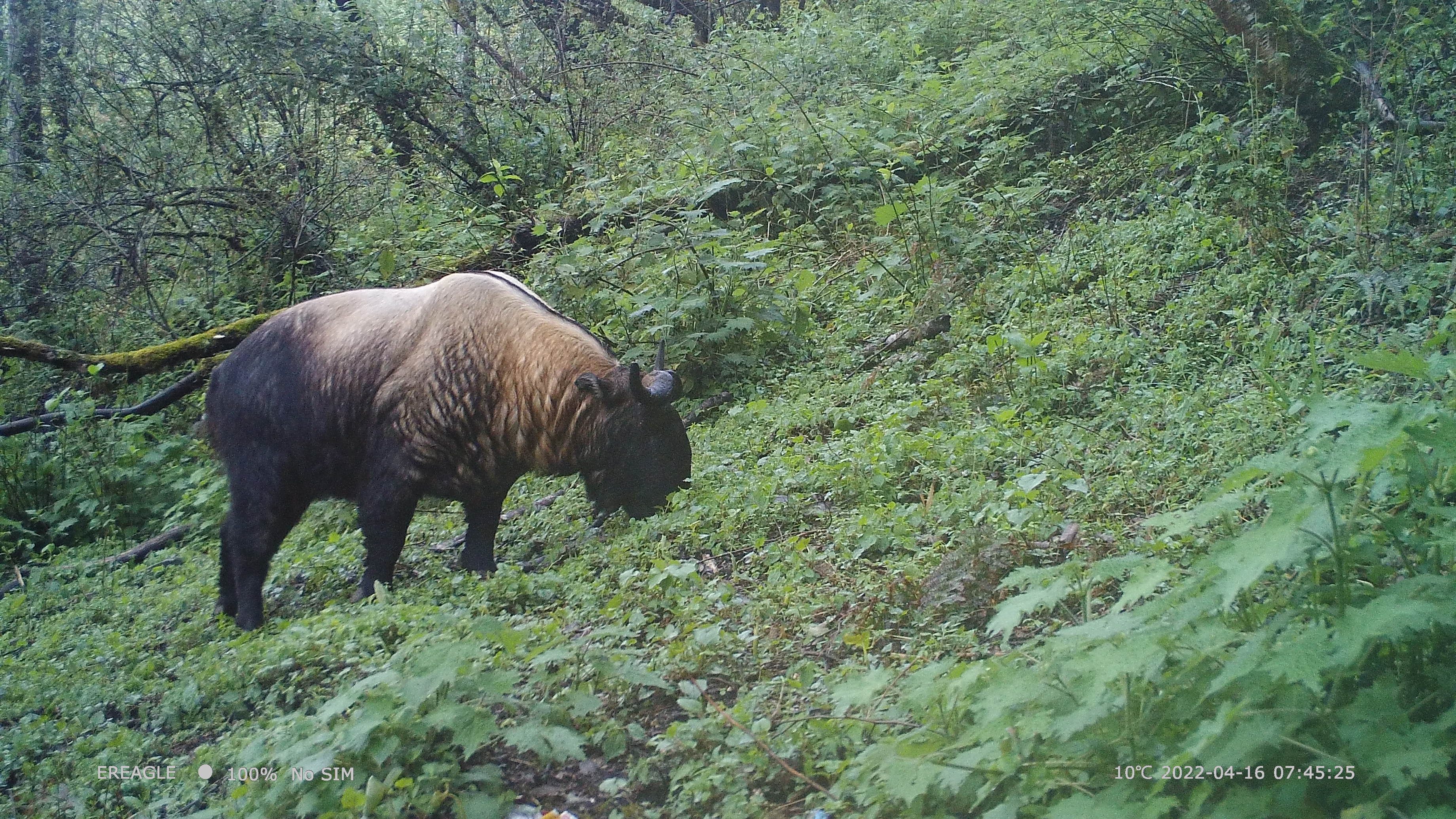

Supplement: Supplementary file 1 [file animals-14-02426-s001.zip › Budorcas taxicolor whitei-Part of the photos/Ere 0581 (3).JPG]

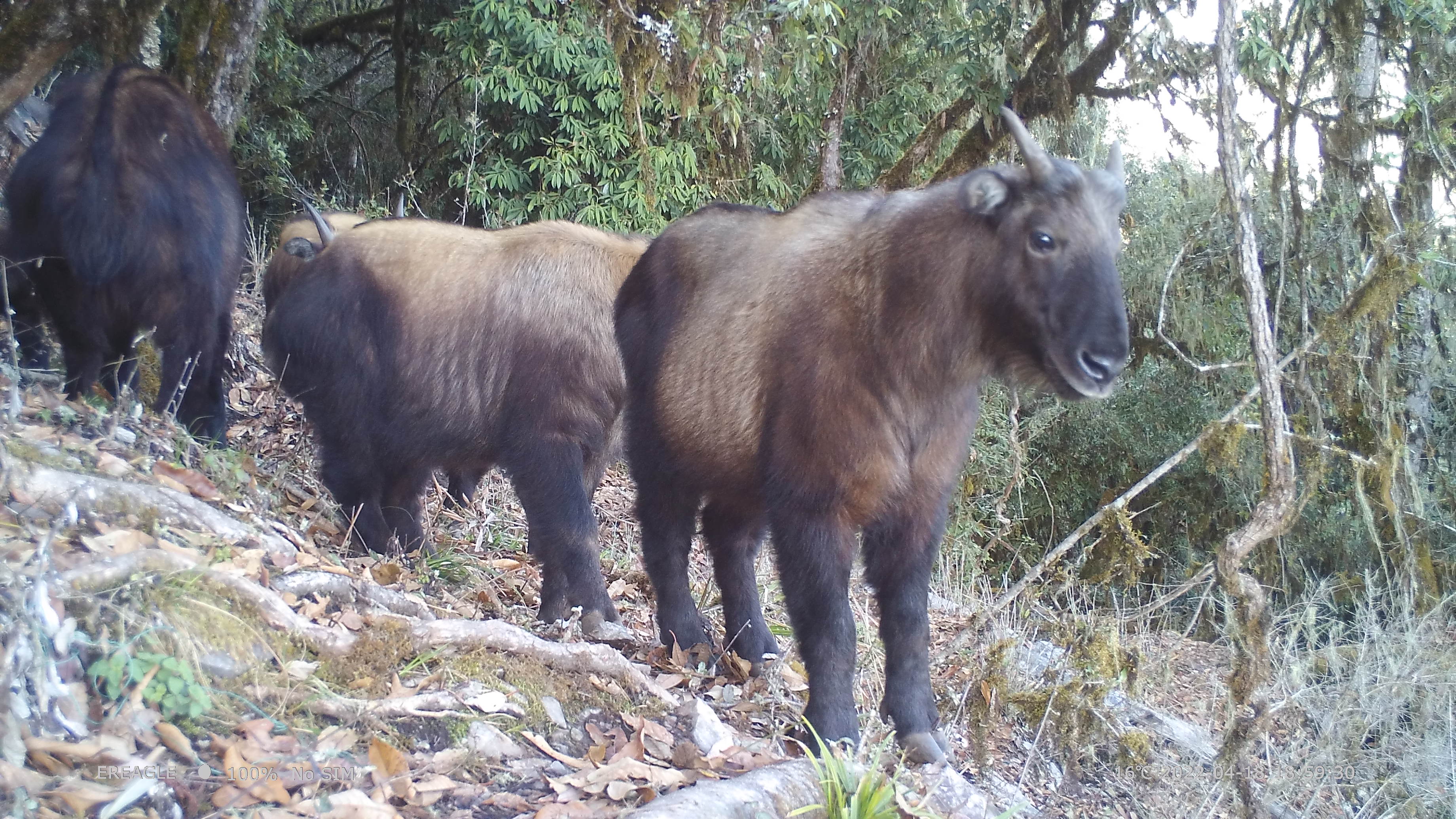

Supplement: Supplementary file 1 [file animals-14-02426-s001.zip › Budorcas taxicolor whitei-Part of the photos/Ere 0608.JPG]

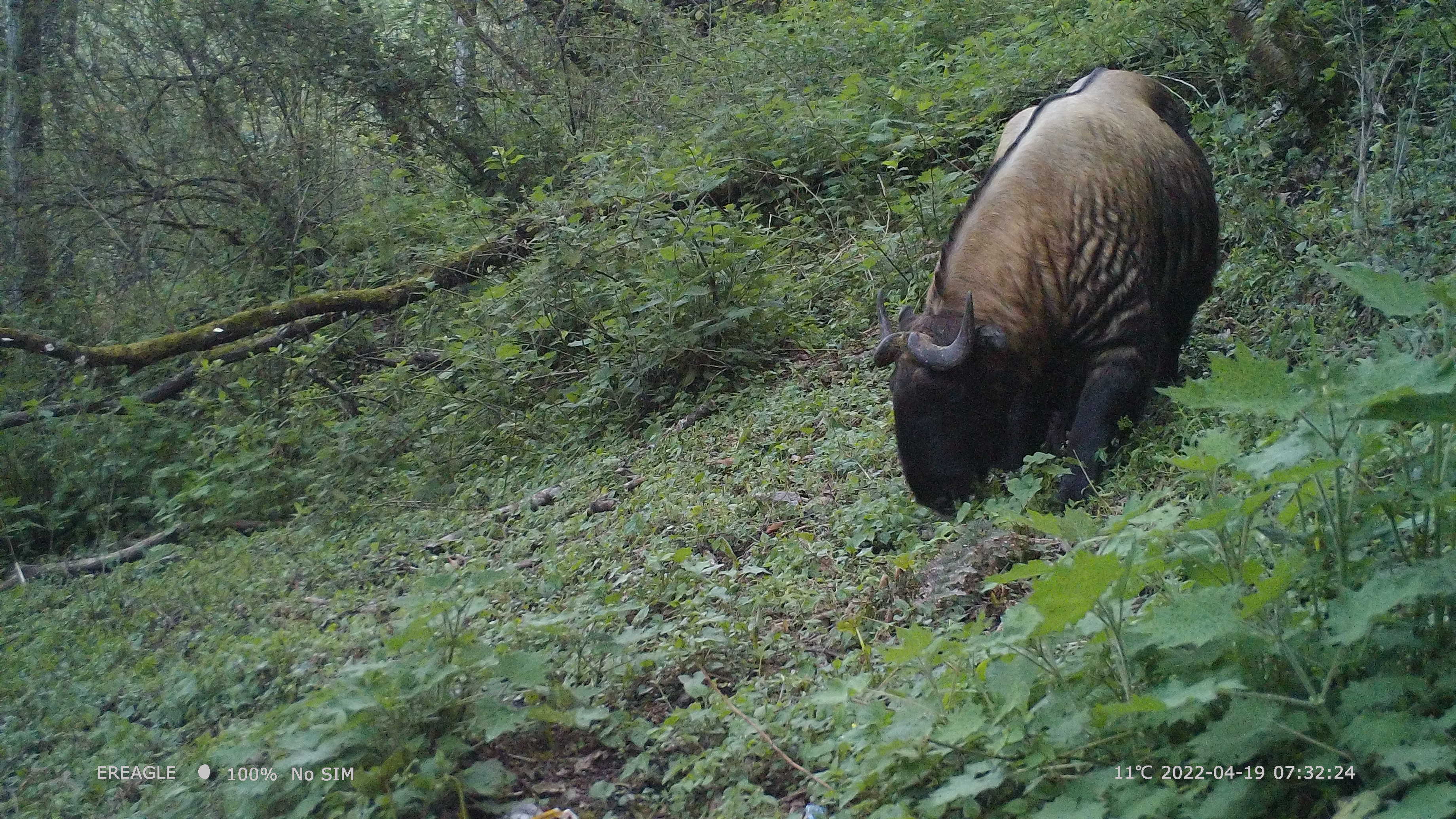

Supplement: Supplementary file 1 [file animals-14-02426-s001.zip › Budorcas taxicolor whitei-Part of the photos/Ere 0618 (3).JPG]

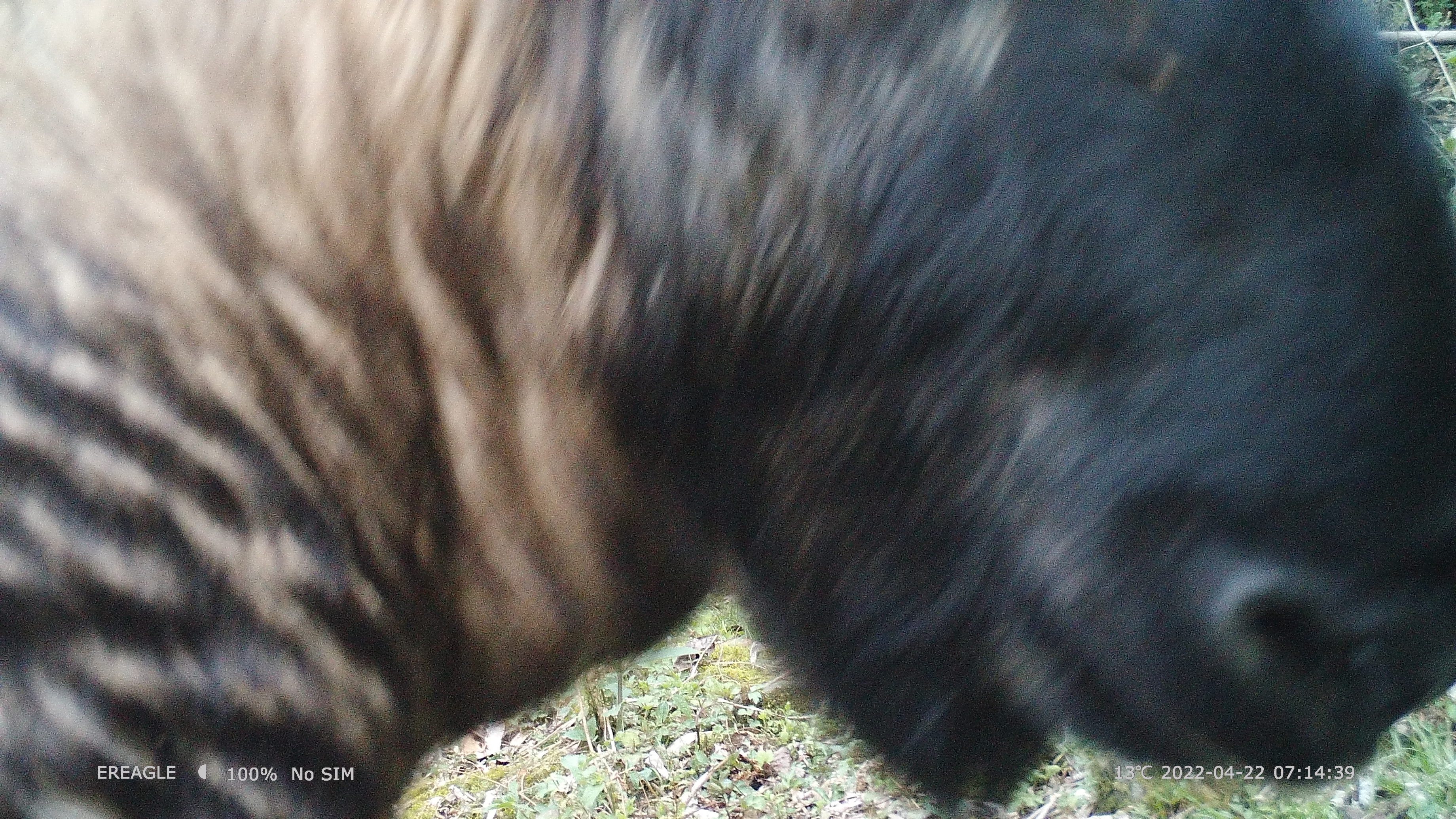

Supplement: Supplementary file 1 [file animals-14-02426-s001.zip › Budorcas taxicolor whitei-Part of the photos/Ere 0621.JPG]

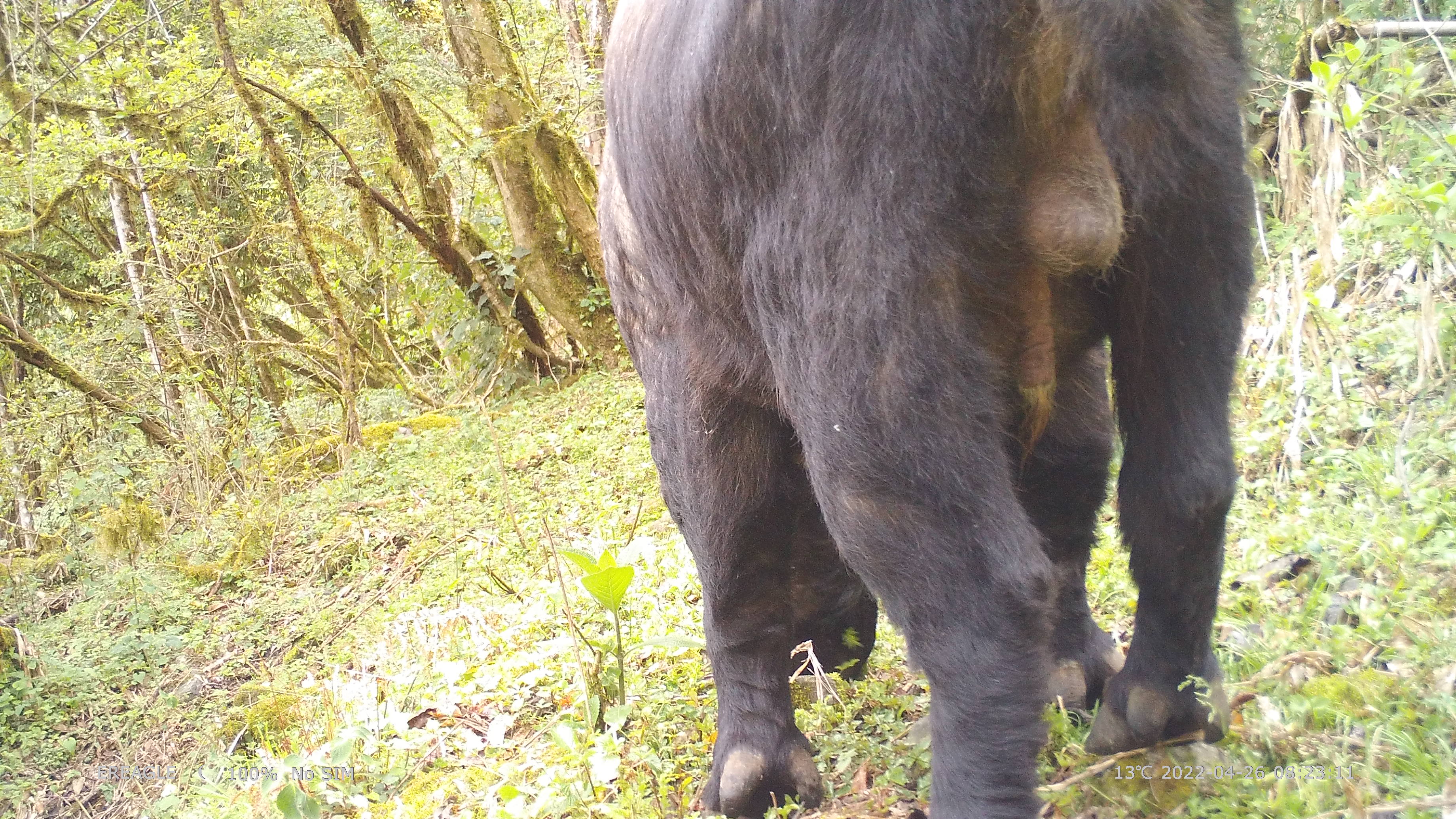

Supplement: Supplementary file 1 [file animals-14-02426-s001.zip › Budorcas taxicolor whitei-Part of the photos/Ere 0669.JPG]

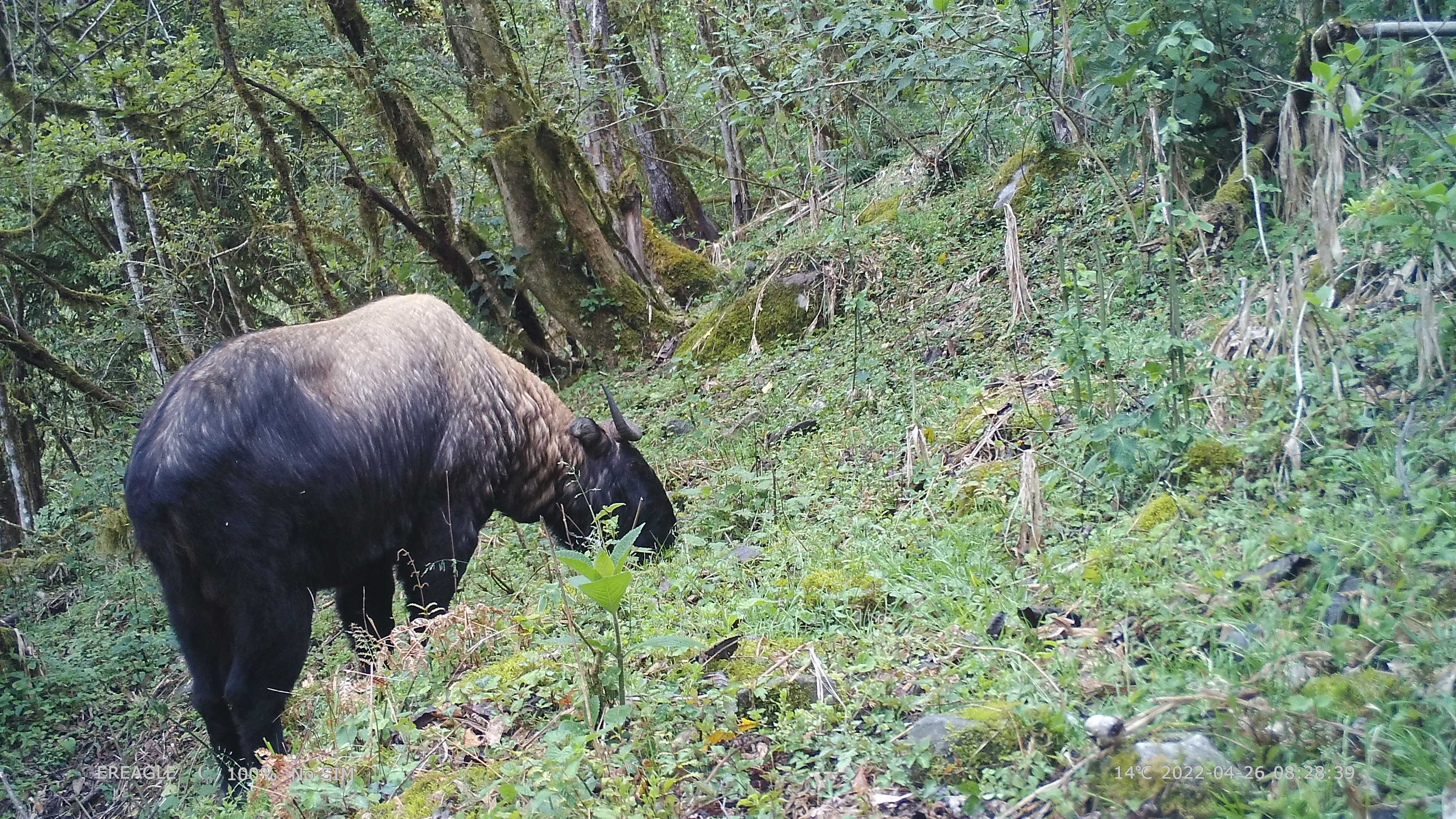

Supplement: Supplementary file 1 [file animals-14-02426-s001.zip › Budorcas taxicolor whitei-Part of the photos/Ere 0682.JPG]

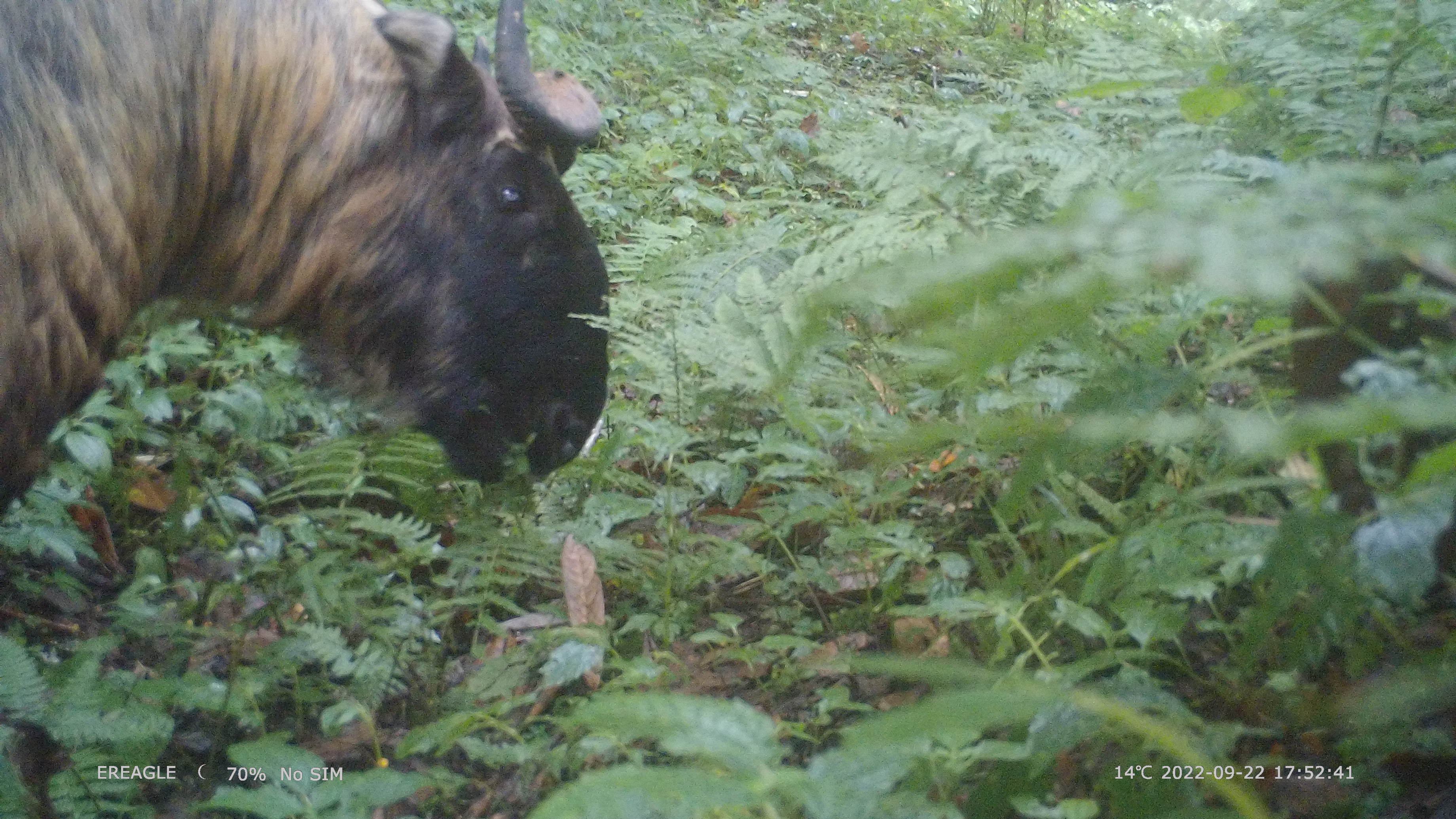

Supplement: Supplementary file 1 [file animals-14-02426-s001.zip › Budorcas taxicolor whitei-Part of the photos/Ere 0689 (3).JPG]

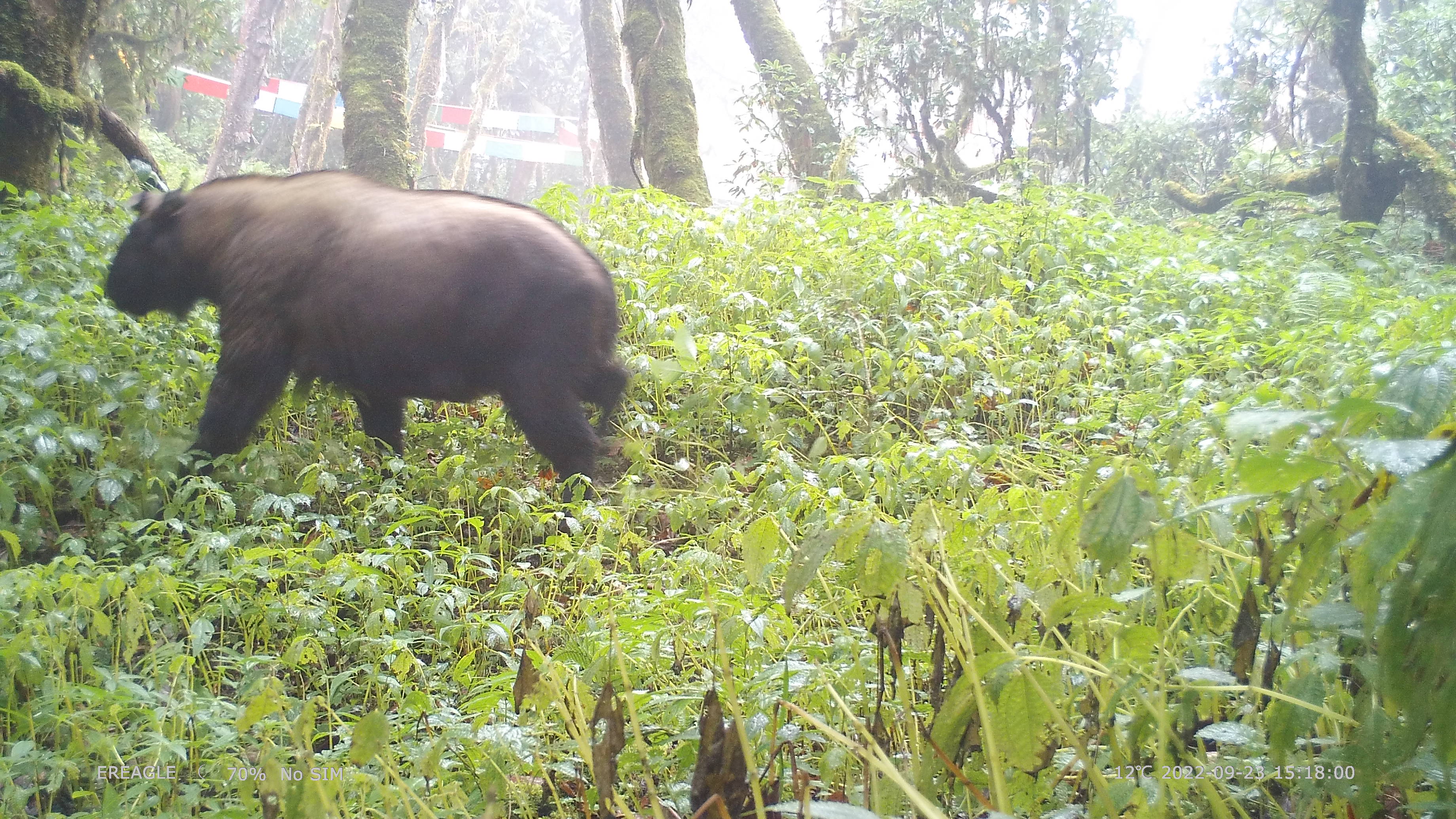

Supplement: Supplementary file 1 [file animals-14-02426-s001.zip › Budorcas taxicolor whitei-Part of the photos/Ere 0812.JPG]

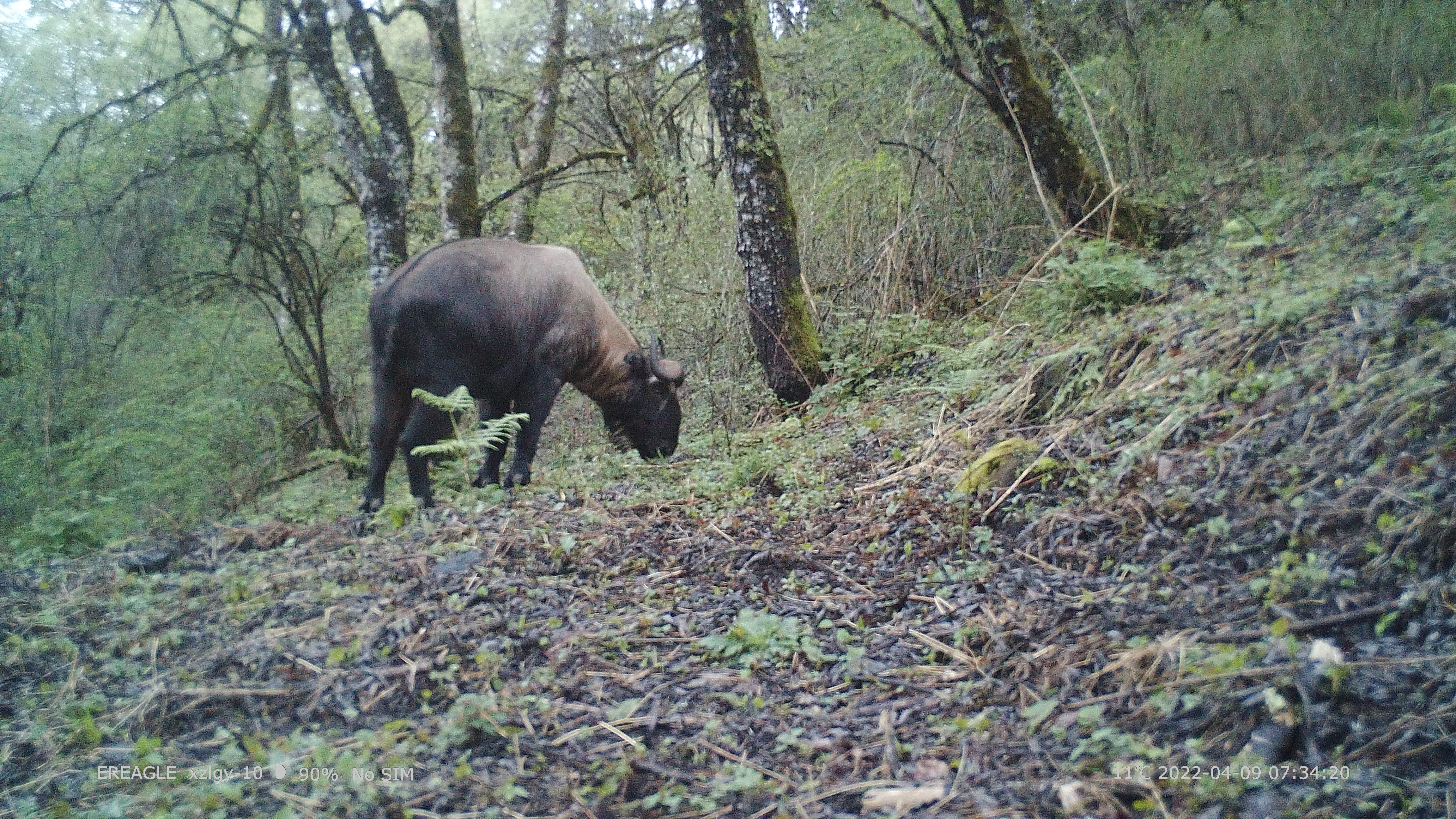

Supplement: Supplementary file 1 [file animals-14-02426-s001.zip › Budorcas taxicolor whitei-Part of the photos/Ere 0857.JPG]

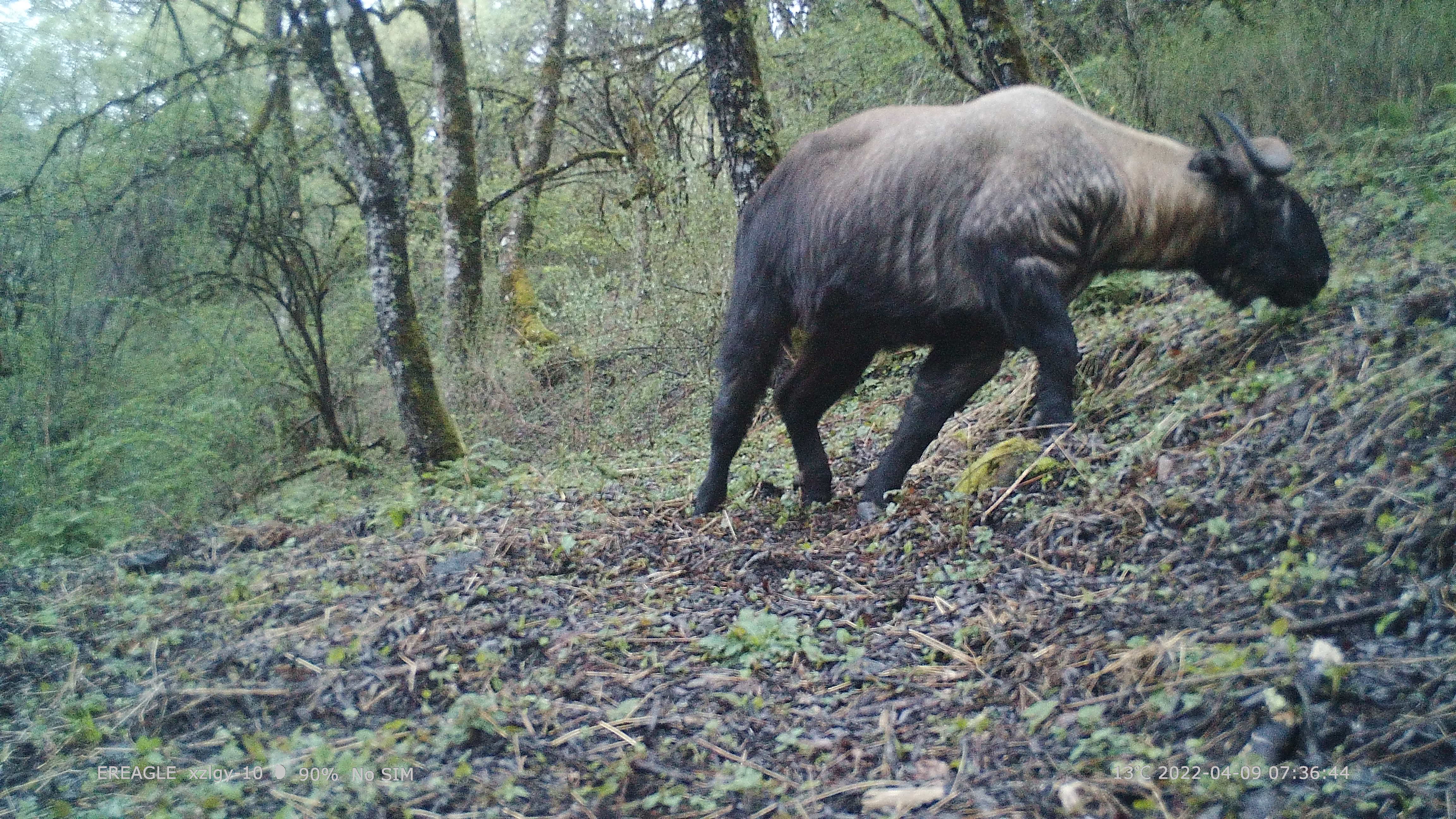

Supplement: Supplementary file 1 [file animals-14-02426-s001.zip › Budorcas taxicolor whitei-Part of the photos/Ere 0870.JPG]

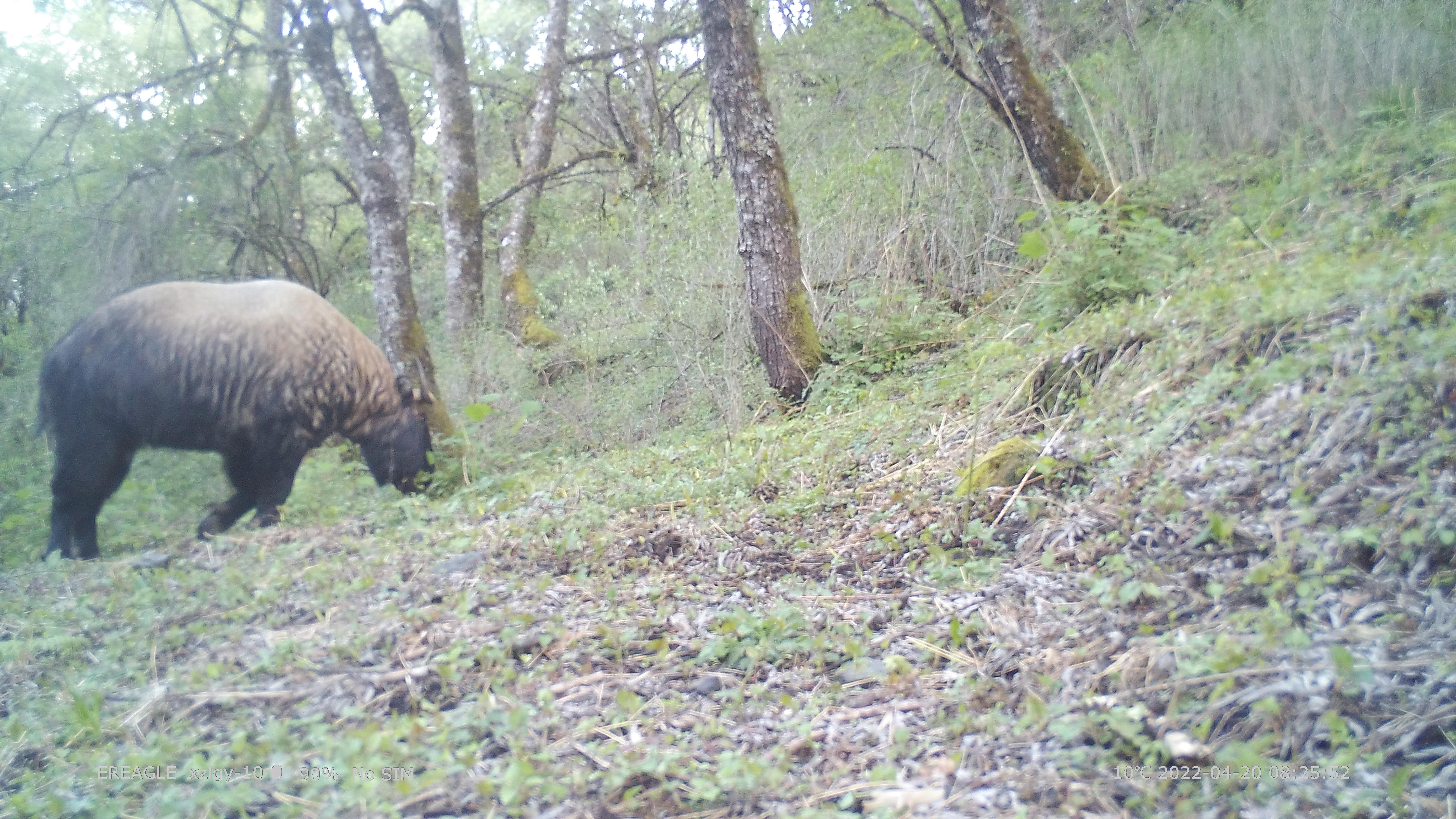

Supplement: Supplementary file 1 [file animals-14-02426-s001.zip › Budorcas taxicolor whitei-Part of the photos/Ere 1082 (2).JPG]

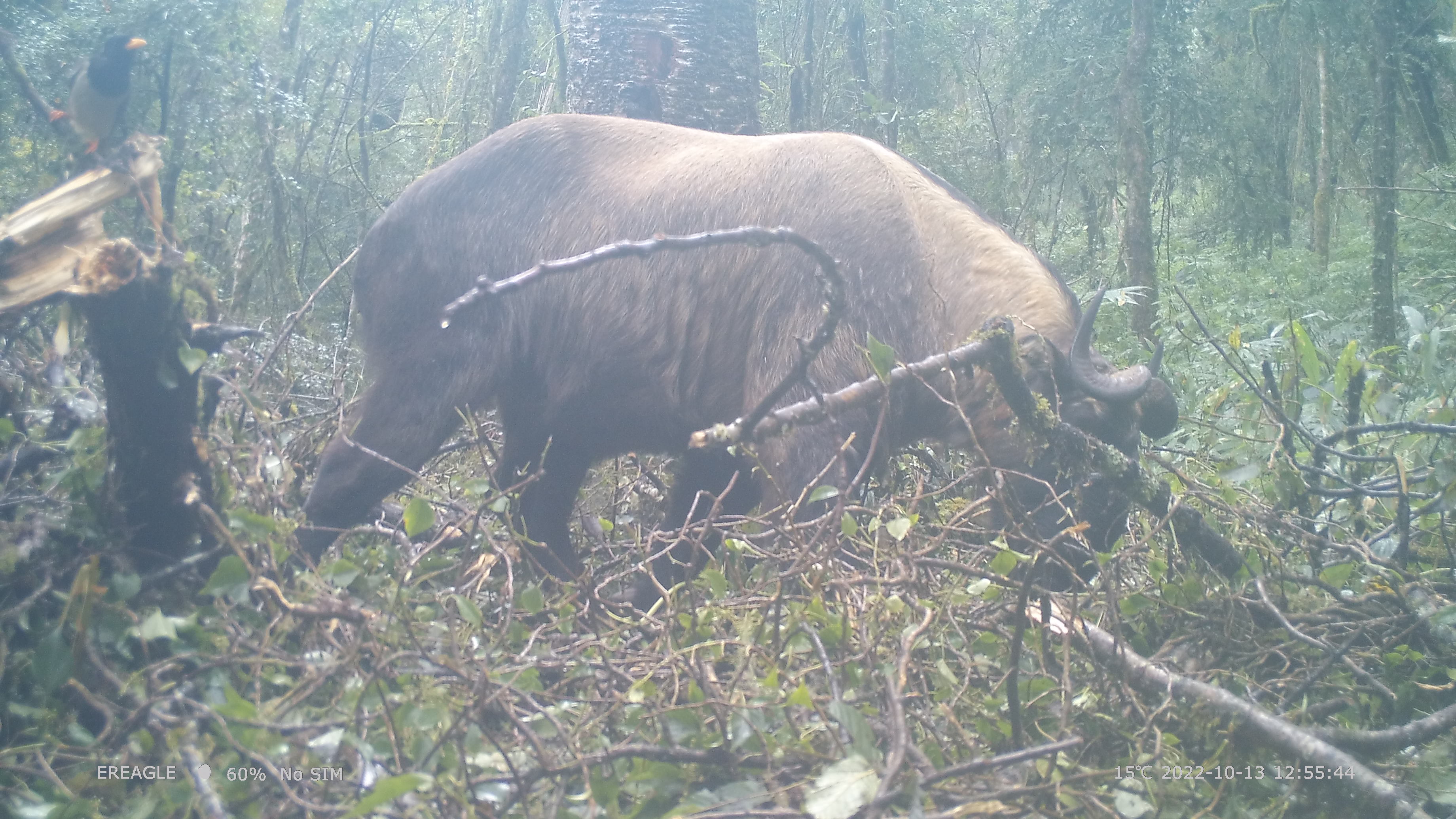

Supplement: Supplementary file 1 [file animals-14-02426-s001.zip › Budorcas taxicolor whitei-Part of the photos/Ere 1160 (2).JPG]

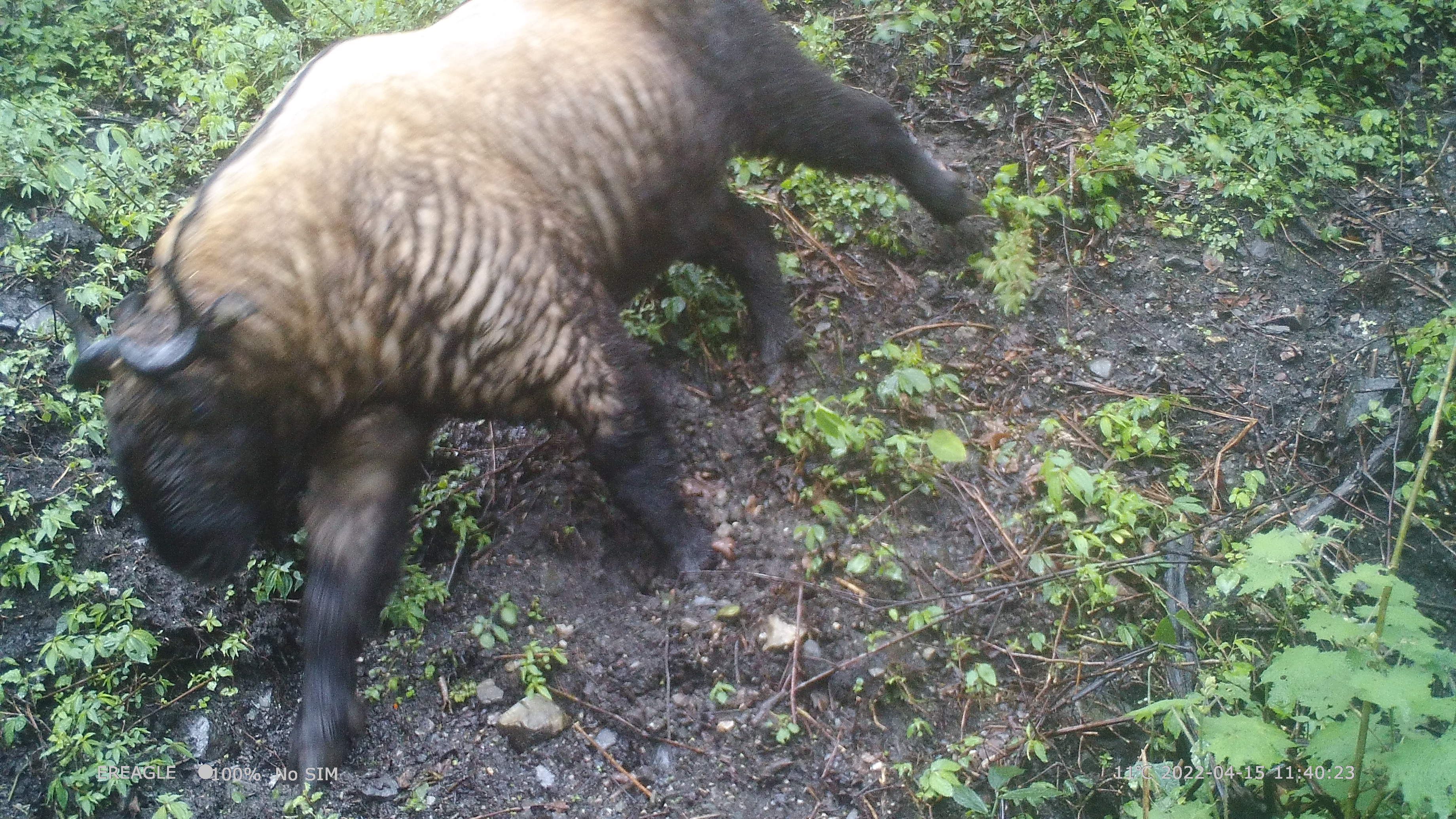

Supplement: Supplementary file 1 [file animals-14-02426-s001.zip › Budorcas taxicolor whitei-Part of the photos/Ere 1361 (2).JPG]

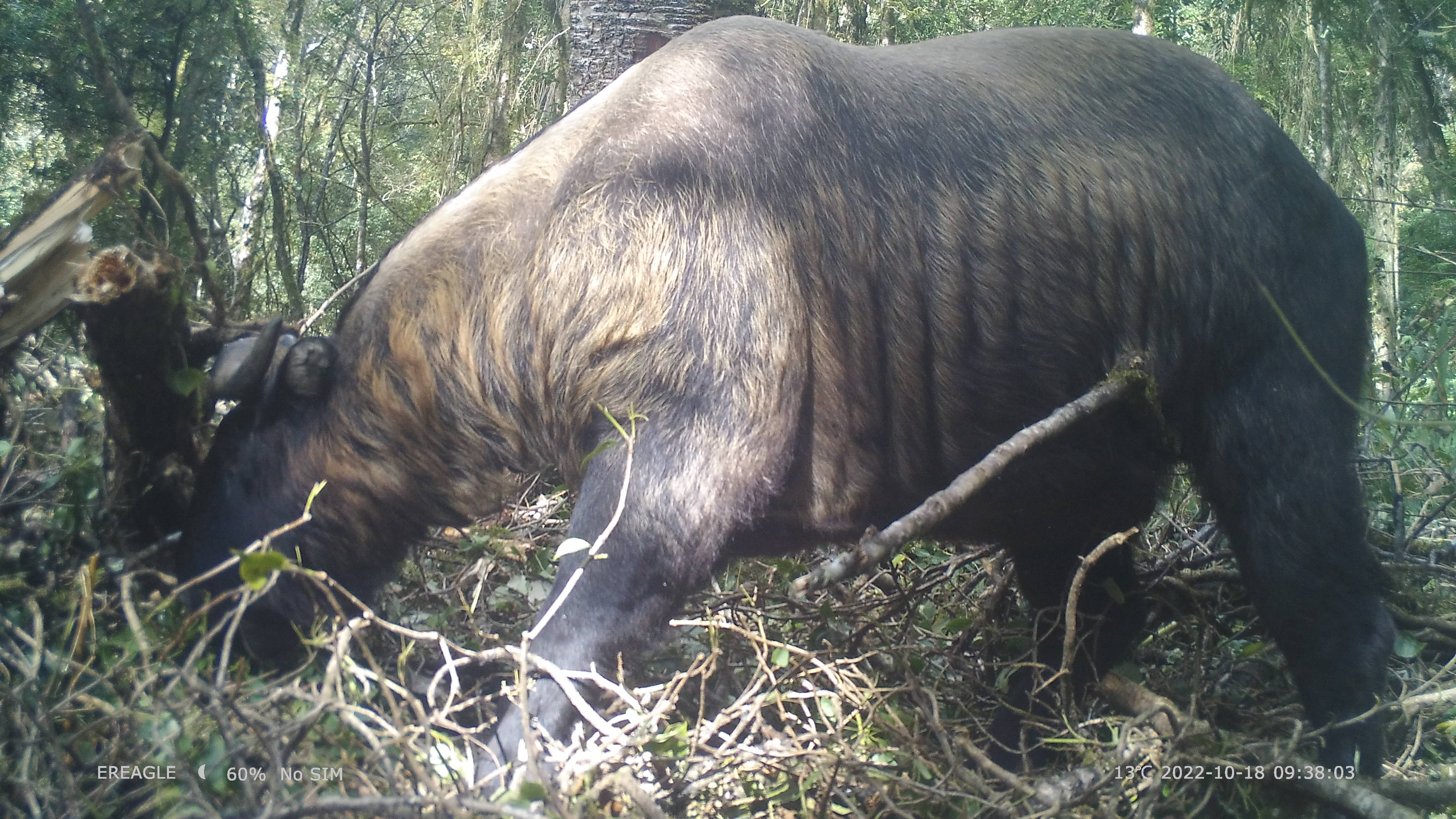

Supplement: Supplementary file 1 [file animals-14-02426-s001.zip › Budorcas taxicolor whitei-Part of the photos/Ere 1372.JPG]

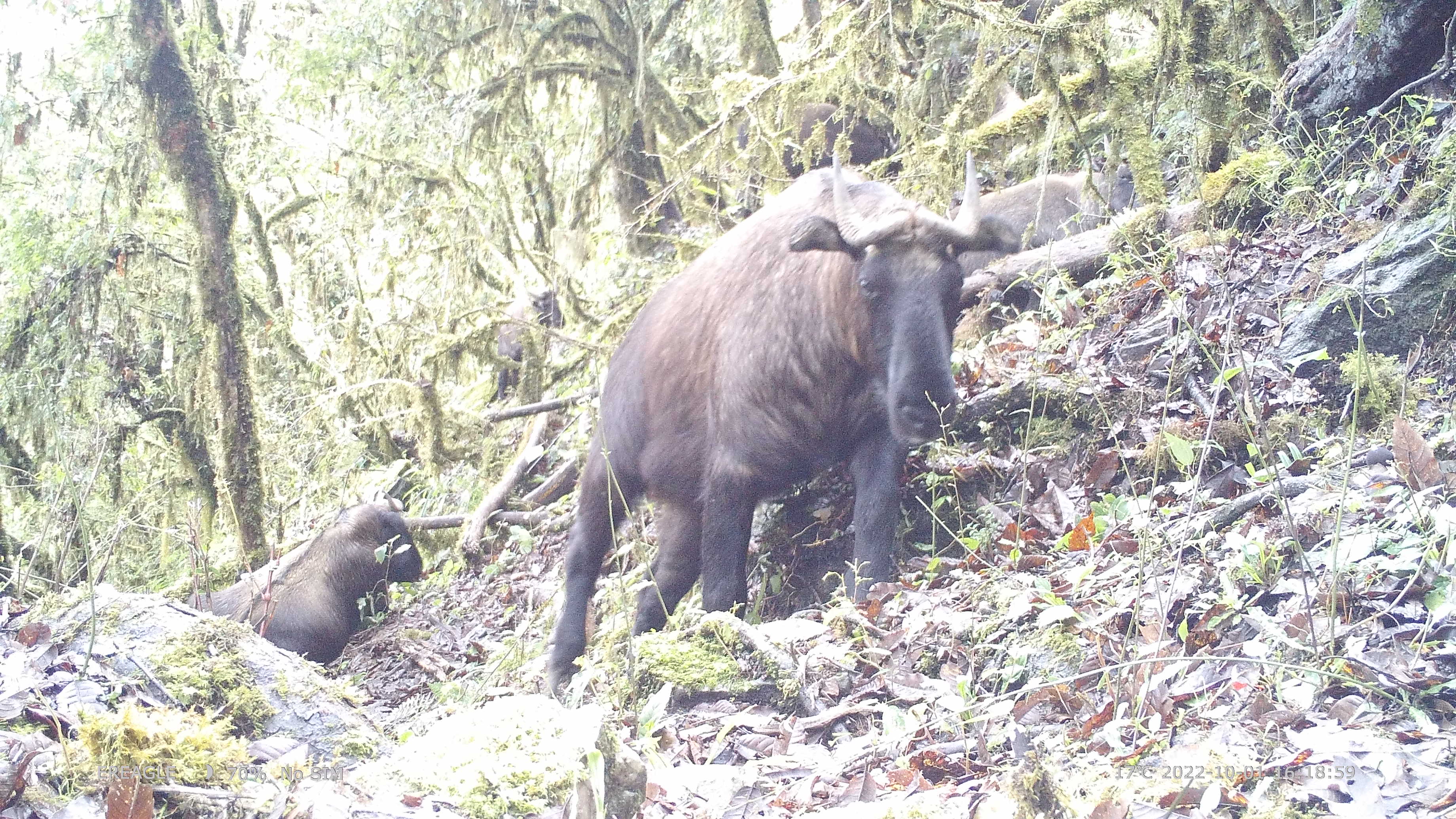

Supplement: Supplementary file 1 [file animals-14-02426-s001.zip › Budorcas taxicolor whitei-Part of the photos/Ere 1495.JPG]

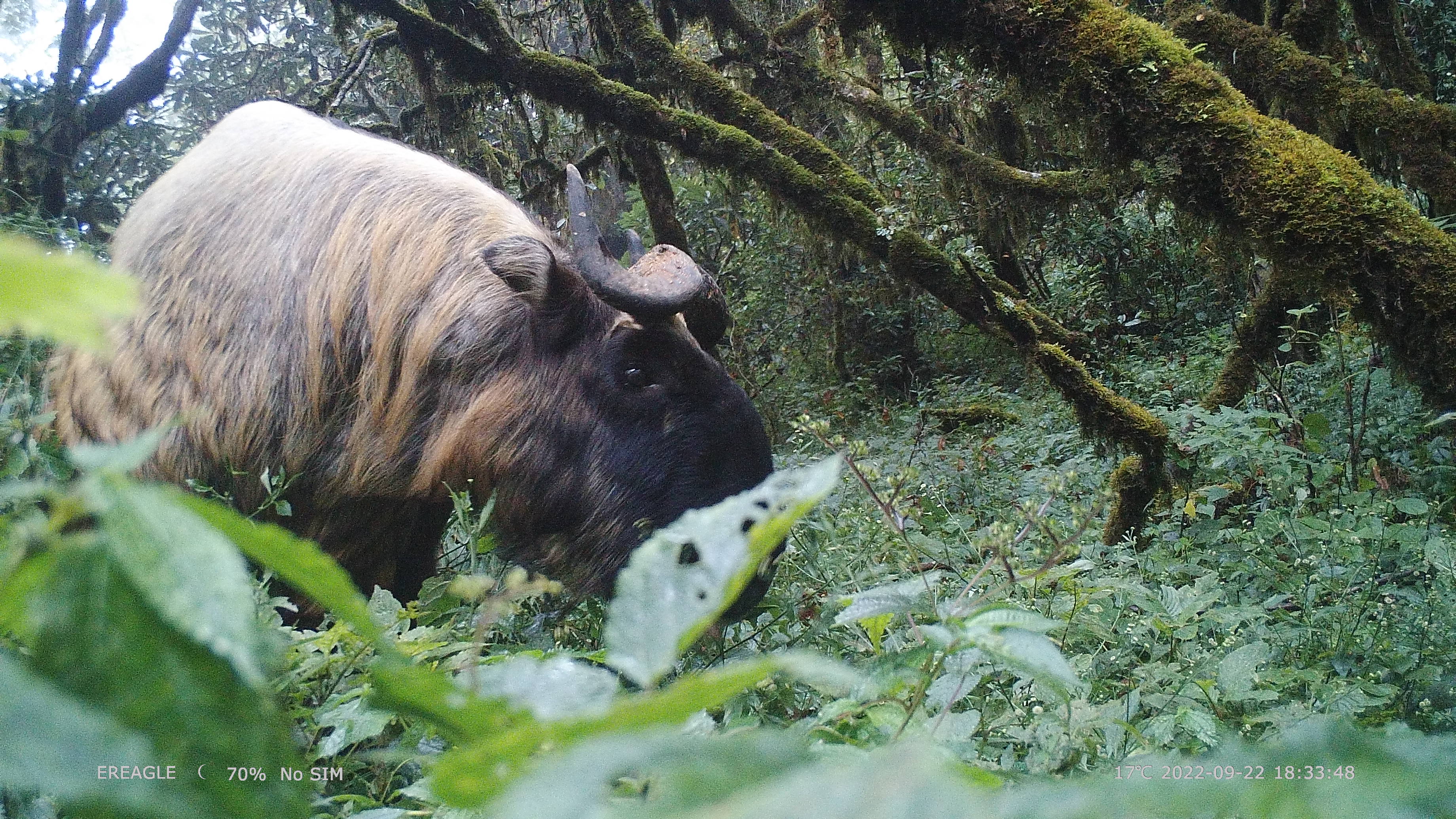

Supplement: Supplementary file 1 [file animals-14-02426-s001.zip › Budorcas taxicolor whitei-Part of the photos/Ere 2007.JPG]

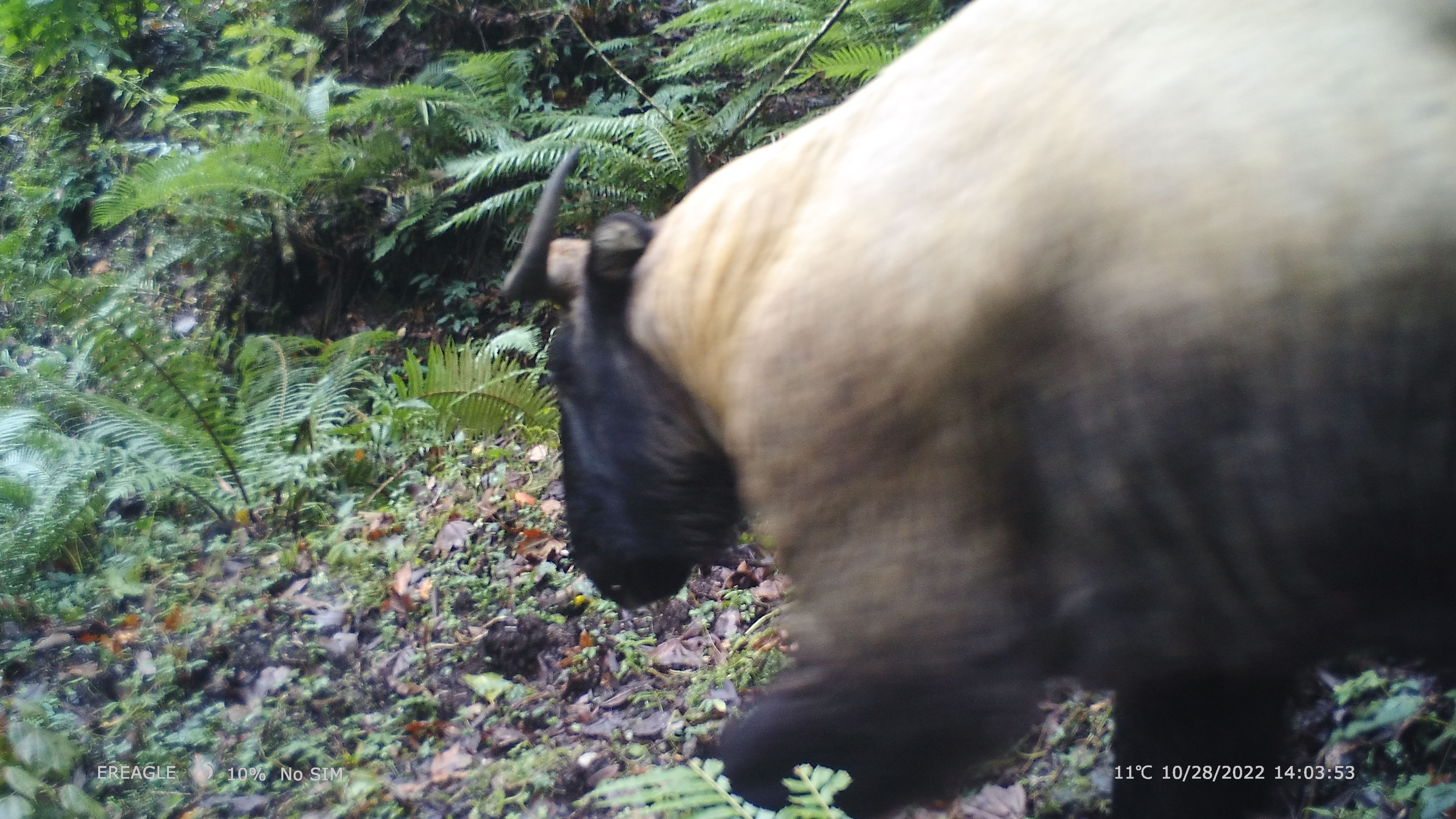

Supplement: Supplementary file 1 [file animals-14-02426-s001.zip › Budorcas taxicolor whitei-Part of the photos/Ere 2884.JPG]
